# Effectiveness of immune checkpoint inhibitors and other treatment modalities in patients with advanced mucosal melanomas: a systematic review and individual patient data meta-analysis

**DOI:** 10.1016/j.eclinm.2024.102870

**Published:** 2024-10-04

**Authors:** Andrea York Tiang Teo, Chun En Yau, Chen Ee Low, Jarett Vanz-Brian Pereira, Julia Yu Xin Ng, Tse Kiat Soong, Jack Yu Tung Lo, Valerie Shiwen Yang

**Affiliations:** a Yong Loo Lin School of Medicine, National University of Singapore, Singapore, 117597, Singapore; b Singapore General Hospital, Singapore, 169608, Singapore; c Internal Medicine, Singapore Health Services, Singapore, 168753, Singapore; d Changi General Hospital, Singapore, 529889, Singapore; e MOH Holdings, Singapore, 099253, Singapore; f Department of Neurosurgery, National Neuroscience Institute, Singapore, 308433, Singapore; g Division of Medical Oncology, National Cancer Centre Singapore, Singapore, 169610, Singapore; h Translational Precision Oncology Laboratory, Institute of Molecular and Cell Biology (IMCB), Agency for Science, Technology and Research (A∗STAR), Singapore, 138673, Singapore; i Oncology Academic Clinical Program, Duke-NUS Medical School, Singapore, 169857, Singapore

**Keywords:** Mucosal melanoma, Immune checkpoint inhibitors, Anti-PD1, Anti-CTLA4, KIT inhibitors, VEGF inhibitors, Radiotherapy

## Abstract

**Background:**

Mucosal melanomas (MM) are an aggressive subtype of melanoma. Given the rarity of this disease, the conduct of clinical trials is challenging and has been limited. Current treatment options have been extrapolated from the more common cutaneous melanoma even though MM is distinct in pathogenesis, etiology and prognosis. This is the first meta-analysis to comprehensively assess the efficacy of immune checkpoint inhibitors (anti-PD1 and anti-CTLA4) and other treatment modalities (targeted therapy such as KIT inhibitors and VEGF inhibitors, as well as radiotherapy) on survival outcomes in MM to develop clinical guidelines for evidence-based management.

**Methods:**

The protocol was prospectively registered on PROSPERO (PROSPERO ID: CRD42023411195). PubMed, Embase, Cochrane Central Register of Controlled Trials (CENTRAL), Web of Science and Google Scholar were searched from inception until 25 July 2024, for all cohort and observational studies. Eligible studies included those with five or more participants with locally advanced or metastatic MM treated with anti-PD1, anti-CTLA4, VEGF inhibitors and/or KIT inhibitors. Titles and abstracts of potential articles were screened and full texts of all potentially eligible studies were retrieved and reviewed by two independent reviewers. Individual patient data (IPD) from published Kaplan–Meier curves were reconstructed using a graphical reconstruction method and pooled as a one-stage meta-analysis. A sensitivity analysis using a two-stage meta-analysis approach was conducted. Extracted outcomes included overall survival (OS) and progression-free survival (PFS). For each treatment arm, median survival time and 12-month survival proportion were estimated. Data from double-arm trials was pooled to estimate hazard ratios (HRs), ratios of restricted mean time lost (RMTL) and restricted mean survival time (RMST).

**Findings:**

From a total of 7402 studies, 35 eligible studies comprising a total of 2833 participants were included. Combined anti-PD1 and anti-CTLA4 therapy had the highest 12-month OS and 12-month PFS at 71.8% (95% CI: 67.6%, 76.2%, n = 476) and 35.1% (95% CI: 30.5%, 40.4%, n = 401) respectively, followed by anti-PD1 therapy alone (OS: 64.0% (95% CI: 61.4%, 66.7%, n = 1399); PFS: was 28.3% (95% CI: 25.8%, 31.2%, n = 1142), anti-PD1 and VEGF inhibitor combination therapy (OS: 57.1% (95% CI: 51.0%, 63.9%)), KIT inhibitors (OS: 48.2% (95% CI: 37.6%, 61.8%); PFS: 8.3% (95% CI: 3.7%, 18.7%)) and anti-CTLA4 therapy alone (OS: 33.3% (95% CI: 28.4%, 39.1%); PFS: 9.8% (95% CI: 5.9%, 16.5%)). In the double-arm studies, combination therapy with anti-PD1 and anti-CTLA4 had similar OS and PFS with anti-PD1 alone (OS: HR 0.856 (95% CI: 0.704, 1.04); RMTL ratio 0.932 (95% CI: 0.832, 1.044, P = 0.225); RMST ratio 1.102 (95% CI: 0.948, 1.281, P = 0.204); PFS: HR 0.919 (95% CI: 0.788, 1.07); RMTL ratio 0.936 (95% CI: 0.866, 1.013, P = 0.100); RMST ratio 1.21 (95% CI: 0.979, 1.496, P = 0.078)), however, anti-PD1 therapy alone had significantly better PFS than anti-CTLA4 alone (HR 0.548 (95% CI: 0.376, 0.799); RMTL ratio 0.715 (95% CI: 0.606, 0.844, P < 0.001); RMST ratio 1.659 (95% CI: 1.316, 2.092, P < 0.001)). Anti-PD1 therapy with radiotherapy versus anti-PD1 alone showed no significant difference (OS: HR 0.854 (95% CI: 0.567, 1.29); RMTL ratio 0.855 (95% CI: 0.675, 1.083, P = 0.193); RMST ratio 1.194 (95% CI: 0.928, 1.536, P = 0.168; PFS: HR 0.994 (95% CI: 0.710, 1.39); RMTL ratio 1.006 (95% CI: 0.87, 1.162, P = 0.939); RMST ratio 0.984 (95% CI: 0.658, 1.472, P = 0.939)).

**Interpretation:**

For the systemic treatment of MM, anti-PD1 is the best monotherapy. While combining anti-PD1 with other treatment options such as anti-CTLA4, VEGF inhibitors or radiotherapy might achieve better outcomes, these improvements did not reach statistical significance when evaluated by HR, RMTL and RMST ratios.

**Funding:**

This work was supported by the National Medical Research Council Transition Award (TA20nov-0020), SingHealth Duke-NUS Oncology Academic Clinical Programme (08/FY2020/EX/67-A143 and 08/FY2021/EX/17-A47), the Khoo Pilot Collaborative Award (Duke-NUS-KP(Coll)/2022/0020A), the National Medical Research Council Clinician Scientist-Individual Research Grant-New Investigator Grant (CNIGnov-0025), the Terry Fox Grant (I1056) and the Khoo Bridge Funding Award (Duke-NUS-KBrFA/2024/0083I).

**Research in context**

**Evidence before this study**

At present, systemic treatment options in mucosal melanomas (MM) are extrapolated from cutaneous melanoma, as the rarity of the disease poses a challenge to developing evidence-based clinical guidelines for MM. Yet, it must be recognized that cutaneous melanomas and MM are two distinct entities, differing in pathogenesis, etiology and prognosis. In cutaneous melanomas, combination anti-PD1 and anti-CTLA4 therapy is now standard of care due to its benefit over monotherapy. It is therefore not uncommon for combination anti-PD1 and anti-CTLA4 therapy to be offered as a first-line systemic treatment in MM, even though its benefit over anti-PD1 monotherapy is unclear. The efficacy of other treatment modalities, such as VEGF inhibitors and KIT inhibitors as targeted therapy in MM, is also yet to be established. Existing studies have shown that VEGF expression is associated with poorer outcomes in patients with MM, although the efficacy of anti-PD1 combined with VEGF inhibitors is unclear. It has also been suggested that radiotherapy in combination with immunotherapy in MM may improve locoregional control, but its impact on overall survival (OS) is uncertain. MM has also been associated with a high incidence of activating mutations and/or amplifications in the KIT oncogene, but the efficacy of KIT inhibitors as a targeted therapy in MM not established.

**Added value of this study**

This systematic review included 35 eligible studies comprising a total of 2833 participants. In the double-arm studies, combination therapy with anti-PD1 and anti-CTLA4 had similar OS and progression-free survival (PFS) with anti-PD1 alone. Furthermore, anti-PD1 therapy alone had significantly better PFS than anti-CTLA4 alone. The efficacy of KIT inhibitors was also considerably lower than anti-PD1 monotherapy.

Combining anti-PD1 with other treatment options such as anti-CTLA4, VEGF inhibitors or radiotherapy demonstrated marginally better outcomes in terms of 12-month OS and PFS, but these improvements did not reach statistical significance when evaluated by hazard ratios, restricted mean time lost (RMTL) and restricted mean survival time (RMST) ratios.

**Implications of all the available evidence**

Overall, our data suggests that for the systemic treatment of MM, anti-PD1 is the best monotherapy. While combining anti-PD1 with other treatment options such as anti-CTLA4, VEGF inhibitors or radiotherapy might achieve better outcomes, these improvements did not reach statistical significance. When weighing between the increased risks of adverse side effects of combination therapy and its non-significant improvement in outcomes over anti-PD1 monotherapy, anti-PD1 monotherapy should be considered as the first-line treatment in advanced MM.

## Introduction

Mucosal melanomas (MM) are a rare and aggressive subtype of melanoma arising from mucosal surfaces, accounting for around 1.3% of all melanomas,^1^ with an annual incidence rate of 1.5 per million.^2^ MM are commonly found to arise from the respiratory, gastrointestinal and genitourinary tracts where melanocytes are present. They have not been found to have a racial predilection, although epidemiological studies have shown that they comprise a higher percentage of overall melanoma cases diagnosed in Asian, African and Hispanic populations.^1^^,^^3^ This could be due to the lower incidence of cutaneous melanomas in these populations, which remains the most common melanoma subtype across all ethnicities.

At present, surgery is the definitive intervention for resectable MM.^4^ However, this is often challenging due to the lentiginous growth pattern of MM and surgically inaccessible locations.^3^^,^^5^ Furthermore, MM are commonly diagnosed late in their disease trajectory and are often invasive when diagnosed, owing to a lack of early symptoms and difficult visual detection.^3^^,^^6^ These factors culminate in a poor prognosis, quality-of-life and mental health outcomes for patients,^7^, ^8^, ^9^, ^10^, ^11^, ^12^ highlighting the need for a consolidated analysis of current data to develop consensus guidelines on optimal systemic therapy, recognizing MM as a distinct entity from other types of melanomas.

While immune checkpoint blockade (ICB) has shown tremendous promise in cutaneous melanoma, with response rates of 70%,^13^^,^^14^ response to ICB in non-cutaneous melanomas is significantly lower.^15^ Additionally, non-cutaneous melanomas are characterized by lower rates of targetable mutations in BRAF and NRAS. MM has also been associated with a high incidence of activating mutations and/or amplifications in the KIT oncogene.^5^^,^^16^^,^^17^ This has therefore generated interest over the utility of KIT inhibitors as a targeted therapy in MM.^6^^,^^18^ Existing studies have shown that VEGF expression is associated with poorer outcomes in patients with MM.^19^^,^^20^ Some have also hypothesized that targeting the tumor microenvironment, including the tumor blood vessels, could improve the efficacy of treatment in melanomas if used in combination with immunotherapy.^21^ However, the literature surrounding the efficacy of VEGF inhibitors remains scant. Hence, VEGF, often overexpressed in melanomas, has also become a target of interest in melanoma treatment in recent years.^22^

Several studies have also explored the utility of radiotherapy as an immune adjuvant. It is believed that radiotherapy, when given at specific volumes, dose fraction sizes, and in specific tumor environments, can either suppress or stimulate the immune system.^23^^,^^24^ It has been suggested that radiotherapy in combination with immunotherapy in MM may improve locoregional control, but its impact on overall survival is less certain.^25^

In this study, we conducted an individual patient data meta-analysis to compare the survival outcomes of patients with MM undergoing immunotherapy, and other treatment modalities including targeted therapy and radiotherapy.

## Methods

### Search strategy

The Preferred Reporting Items for Systematic Reviews and Meta-Analyses (PRISMA) guidelines informed the design and execution of this study. The protocol was prospectively registered on PROSPERO (PROSPERO ID: CRD42023411195, registered 1st July 2022). PubMed, Embase, Cochrane Central Register of Controlled Trials (CENTRAL), Web of Science and Google Scholar were searched from inception until 25 July 2024. Keywords related to MM, immunotherapy and other treatment modalities were used in the search. The full search strategy is reported in Supplementary Data 1. References from included studies and review papers were systematically hand searched to include studies omitted by the electronic search.

### Eligibility criteria

We included studies evaluating the efficacy of immune checkpoint inhibitors in the treatment of patients with MM. We included only cohort and observational studies which (a) had five or more participants, (b) with locally advanced or metastatic MM treated with anti-PD1, anti-CTLA4, VEGF inhibitors and/or KIT inhibitors, (c) reported on overall survival (OS) or progression-free survival (PFS) and (d) published Kaplan–Meier curves amenable for graphical reconstruction. Studies were excluded if they (a) did not publish Kaplan–Meier curves, (b) did not publish data on patients with MM, (c) not in English and (d) were reviews, case reports, case series, letters, comments, editorials, conference proceedings, trial registrations, and personal communications.

### Data extraction

The titles and abstracts of potential articles were screened against the eligibility criteria independently by four authors, and any discrepancies were resolved with an independent reviewer by discussion. The full texts of all potentially eligible studies were then retrieved and reviewed by two independent reviewers, with any discrepancies resolved by common consensus. Baseline information including age, gender and comorbidities were collected. The primary outcomes of interest were OS and PFS. Among the studies which included the definitions of OS and PFS, OS was defined as the time from treatment initiation until the date of death, while PFS was defined as the time of treatment initiation to radiologic or clinical progression.^26^, ^27^, ^28^, ^29^, ^30^, ^31^, ^32^, ^33^^,^^24^^,^^34^, ^35^, ^36^, ^37^, ^38^, ^39^, ^40^, ^41^, ^42^, ^43^, ^44^, ^45^

### Statistical analysis

Individual patient data (IPD) from published Kaplan–Meier curves were reconstructed using Guyot et al.‘s^46^ graphical reconstruction method and pooled as a one-stage meta-analyses. Using digitised images of Kaplan–Meier curves, we calculated step functions and timing values. Survival information of individual patients from each treatment arm was recovered using numerical solutions to the inverted Kaplan–Meier product-limit equations. The reconstructed IPD dataset was checked visually against the original curves (Supplementary Data 2) and compared to the original log-rank values when possible. The results were quantitatively pooled and analysed using RStudio (Version 4.1.2). We calculated OS and PFS using the Kaplan–Meier method. For the analysis of cumulative incidence, we used the stratified Cox model, which models inter-study heterogeneity by assuming a baseline hazard for patients from each unique study.^47^ For each treatment type, we estimated the median survival time and 12-month survival proportion. We pooled data from the double-arm studies to calculate the hazard ratios (HRs) and their 95%-confidence intervals (95% CI). The proportional hazards assumption by testing for the presence of a non-zero slope in a generalised linear regression of scaled Schoenfeld residuals on time using the R package “survival” and R function “cox.zph” (Supplementary Table S1). Due to possible non-proportionality of hazards, we performed analysis of the ratios of restricted mean time lost (RMTL) and restricted mean survival time (RMST) using the survRM2 R package.^48^^,^^49^ The restricted mean measures average survival from time 0 to a specified time point and may be estimated as the area under (RMST) or above (RMTL) the survival curve up to that point.^50^ For the purposes of this study, we chose the timepoint based on the default tau value in the survRM2 R package, as it makes full use of the available data to capture the long-term benefits of the various treatment modalities. RMST and RMTL ratios have been used in a variety of clinical studies,^50^, ^51^, ^52^, ^53^ yielding similar statistical conclusions as HR analysis without the model assumptions of proportional hazard analysis. The RMTL ratio is calculated by RMTL of experimental intervention/RMTL control intervention, with an RMTL ratio <1 indicating superiority of the experimental intervention.^54^ Conversely, an RMST ratio of >1 indicates superiority of the experimental intervention.

As a sensitivity analysis, we followed the approaches outlined in the Cochrane Handbook^55^ and conducted two-stage meta-analyses of survival proportions and hazard ratios the R package *meta*. A random-effects model was applied in all analyses due to the high clinical likelihood that the included study populations are heterogeneous,^56^^,^^57^ and to allow generalisation of the results beyond the included studies. Proportions were analysed using a generalized linear mixed model a logit transformation of proportions. Between-study heterogeneity was evaluated using I^2^ statistics, where an I^2^ value of 25% indicates low heterogeneity,^58^^,^^59^ 50% indicates moderate heterogeneity, and 75% indicates high heterogeneity. Two-sided P values < 0.05 were regarded to indicate nominal statistical significance. Publication bias was not assessed as there were fewer than ten studies which reported on the hazard ratios of the treatment options when compared to each other in patients with MM.^60^

### Risk of bias assessment

Risk of bias in the studies were assessed independently by two reviewers using the Joanna Briggs Institute Critical appraisal (JBI) checklist tool and discrepancies were resolved by discussion.^61^

### Role of funding

The funder of the study had no role in study design, data collection, data analysis, data interpretation, or writing of the report.

## Results

**Fig. 1:**
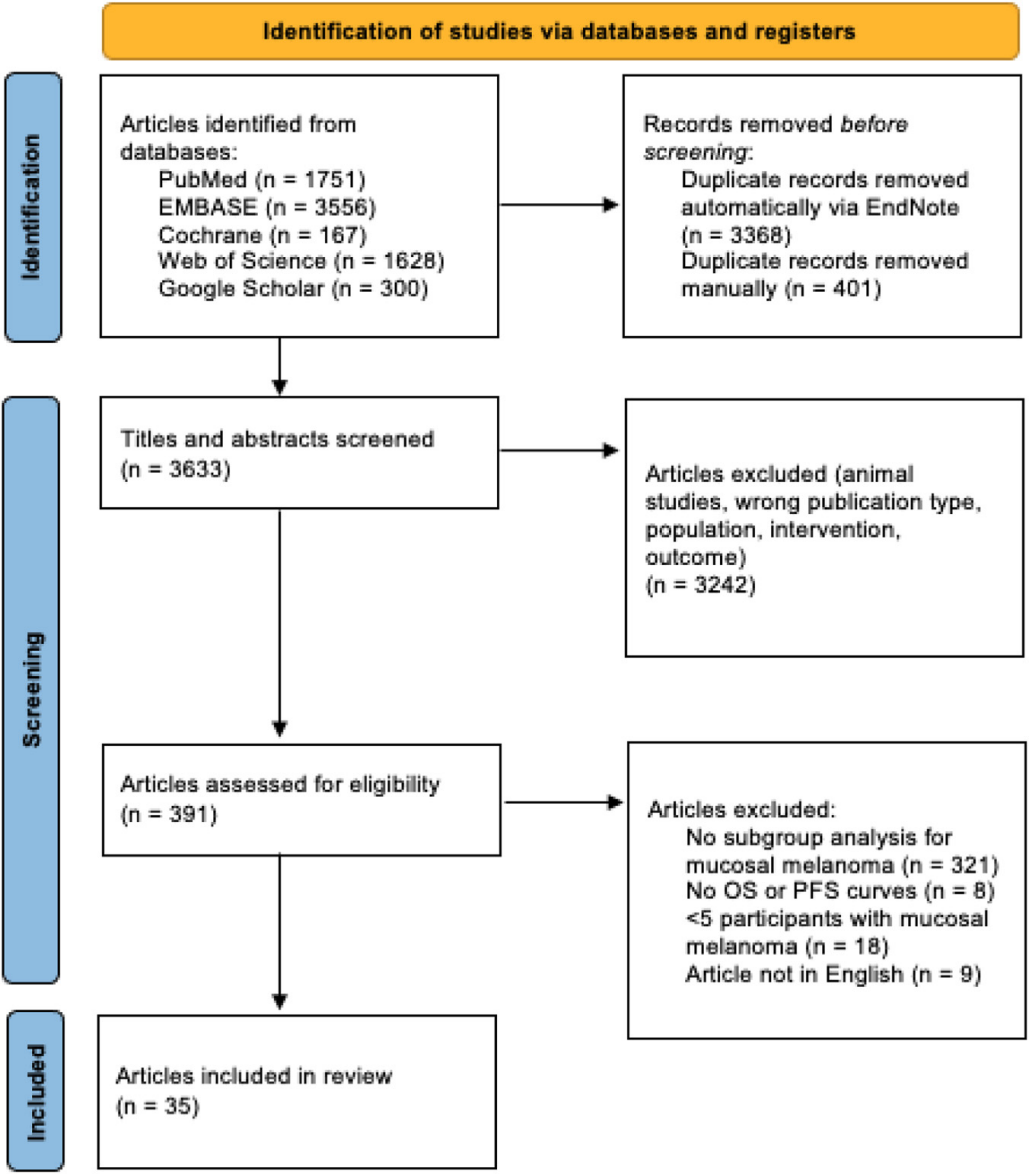
PRISMA flowchart.

The PRISMA flow diagram is presented in Fig. 1. From 7402 articles initially identified, we included a total of 35 studies.^24^^,^^26^, ^27^, ^28^, ^29^, ^30^, ^31^, ^32^, ^33^, ^34^,^36^, ^37^, ^38^, ^39^, ^40^, ^41^, ^42^, ^43^, ^44^, ^45^,^62^, ^63^, ^64^, ^65^, ^66^, ^67^, ^68^, ^69^, ^70^ The remaining 7367 studies were excluded after removing duplicates, irrelevant study types and outcomes, and studies without available data for the MM subgroup.

### Baseline characteristics

The characteristics of the included studies are summarised in Table 1. Seven studies were double-armed and directly compared two or more treatment modalities.^24^^,^^30^, ^31^, ^32^, ^33^^,^^35^,^64^^,^^66^^,^^71^, ^72^, ^73^, ^74^, ^75^ A total of 2833 participants were evaluated. The specific intervention conducted by the included studies and the number of subjects investigated per intervention are detailed in Tables 2 and 3.

**Table 1:** Overall characteristics of patients in the included studies.

Author	Region of study	Systemic therapy studied	Gender male n (%)	Number of participants	Median age in years (range)	Primary site of MM n (%)	Baseline ECOG status n (%)	Disease stage n (%)	Previous lines of systemic therapy	Median follow-up duration (range)	Mutational status n/patients tested (%)
Alexander 2014	Australia	Ipilimumab	NR^a^	8	>65	NR	0-2–8 (100)	NR	NR	NR^a^	NR^a^
Del Vecchio 2014	Europe	Ipilimumab	27 (38)	71 (69 evaluated for efficacy)	63 (22–85)	Gastrointestinal–28 (39) Urogenital—18 (25) Head and neck—22 (31) Others—3 (4)	0–40 (56) 1–27 (38) ≥2–4 (6)	Locally advanced—0 Metastatic—71 (100)	0–0 (0) 1–47 (66) 2–14 (20) ≥3–10 (14)	21.8 months (1.0–32.7)	BRAF—2/31 (6) KIT—2/12 (17) NRAS—0/3 (0)
Yamazaki 2020	Asia	Ipilimumab	NR^a^	180	NR^a^	NR	NR^a^	NR^a^	NR^a^	NR	NR^a^
Postow 2013	USA	Ipilimumab	14 (42)	33 (30 evaluated for efficacy)	65 (35–90)	Gastrointestinal—9 (27) Urogenital—11 (33) Head and neck—13 (39) Others—0 (0)	0–19 (58) 1–12 (36) ≥2–2 (6)	Locally advanced—2 (6) Metastatic—31 (94)	0–8 (24) 1–17 (52) 2–6 (18) ≥3–2 (6)	9.9 months (5.8–20.2)	BRAF—1/22 (5) KIT—4/25 (16) NRAS—6/20 (30)
Zimmer 2015	Europe	Ipilimumab	2 (29)	7	63 (33–37)	NR	0–2 (29) 1–5 (71) ≥2–0 (0)	Locally advanced—0 (0) Metastatic—7 (100)	0–0 (0) 1–8 (86) 2–0 (0) ≥3–1 (14)	NR	BRAF—0/3 (0)
Moya-Plana 2019	Europe	–	16 (36)	44	63 (24–88)	Gastrointestinal—14 (32) Urogenital—12 (27) Head and neck—18 (41) Others—0 (0)	0–28 (64) 1–16 (36) ≥2–0 (0)	Locally advanced—11 (25) Metastatic—19 (43)	17 (39) with prior systemic therapy	24 months (4–73)	BRAF—0/44 (0) KIT—2/44 (5) NRAS—3/44 (7)
		Ipilimumab	–	24	–	–	–	–	–	–	–
		Pembrolizumab	–	20	–	–	–	–	–	–	–
Namikawa 2018	Asia	Nivolumab + Ipilimumab	NR^a^	12	NR^a^	NR	NR^a^	NR^a^	NR^a^	NR^a^	NR^a^
Hodi 2021	USA	Nivolumab + Ipilimumab	NR^a^	47	NR^a^	NR	NR^a^	NR^a^	NR^a^	NR^a^	NR^a^
Kottschade et al.	USA	Nivolumab + Ipilimumab	14 (40)	35	67 (39–80)	Gastrointestinal—9 (25.7) Urogenital—12 (34.3) Head and neck—10 (28.6) Others—4 (11.4)	0-1–35 (100)	Locally advanced—15 Metastatic—2 NR—17	NR	36.5 months	BRAF—1/19 KIT—3/15 NRAS—1/11
Takahashi 2023	Japan	Nivolumab + Ipilimumab	NR^a^	36	NR^a^	NR^a^	NR^a^	NR^a^	NR^a^	NR^a^	NR^a^
Umeda 2021	Asia	–	81 (36)	225	–	Gastrointestinal—43 (19) Urogenital—58 (26) Head and neck—124 (55) Others—0 (0)	0–148 (66) ≥1–77 (34)	Locally advanced—53 (24) Metastatic—172 (76)	0–225 (100) 1–0 (0) 2–0 (0) ≥3–0 (0)	NR	BRAF—4/200 (2)
		Anti-PD1 alone	38 (33)	115	71 (29–88)	Gastrointestinal—23 (20) Urogenital—32 (28) Head and neck—60 (52) Others—0 (0)	0–80 (70) ≥1–35 (30)	Locally advanced—31 (27) Metastatic—84 (73)	0–115 (100) 1–0 (0) 2–0 (0) ≥3–0 (0)	NR	BRAF—2/107 (2)
		Anti-PD1 + RT	20 (36)	56	69 (34–89)	Gastrointestinal—7 (12) Urogenital—11 (20) Head and neck—38 (68) Others—0 (0)	0–29 (52) ≥1–27 (48)	Locally advanced—13 (23) Metastatic—43 (77)	0–56 (100) 1–0 (0) 2–0 (0) ≥3–0 (0)	NR	BRAF—0/43 (0)
		Anti-PD1 + anti-CTLA4	16 (38)	42	62 (22–83)	Gastrointestinal—11 (26) Urogenital—10 (24) Head and neck—21 (50) Others—0 (0)	0–31 (74) ≥1–11 (26)	Locally advanced—6 (14) Metastatic—36 (86)	0–42 (100) 1–0 (0) 2–0 (0) ≥3–0 (0)	NR	BRAF—2/38 (5)
		Anti-PD1 + anti-CTLA4 + RT	7 (58)	12	66 (44–86)	Gastrointestinal—2 (16) Urogenital—5 (42) Head and neck—5 (42) Others—0 (0)	0–8 (67) ≥1–4 (33)	Locally advanced—3 (25) Metastatic—9 (67)	0–12 (100) 1–0 (0) 2–0 (0) ≥3–0 (0)	NR	BRAF—0/12 (0)
Nakamura 2021	Asia	–	130 (40)	329	70 ([IQR] 63–76)	Gastrointestinal—69 (21) Urogenital—76 (23) Head and neck—184 (56)	0–235 (71) ≥1–94 (29)	Locally advanced—70 (21) Metastatic—259 (79)	0–329 (100) 1–0 (0) 2–0 (0) ≥3–0 (0)	NR	BRAF—7/292 (2)
		Anti-PD1	102 (39)	263	71 ([IQR] 65–77)	Gastrointestinal—54 (20) Urogenital—57 (22) Head and neck—152 (58)	0–185 (70) ≥1–78 (30)	Locally advanced—58 (22) Metastatic—205 (78)	0–263 (100) 1–0 (0) 2–0 (0) ≥3–0 (0)	–	BRAF—4/232 (2)
		Anti-PD1 + anti-CTLA4	28 (42)	66	65 ([IQR] 58–73)	Gastrointestinal—15 (23) Urogenital—19 (29) Head and neck—32 (48)	0–50 (76) ≥1–16 (24)	Locally advanced—12 (18) Metastatic—54 (82)	0–66 (100) 1–0 (0) 2–0 (0) ≥3–0 (0)	–	BRAF—3/60 (5)
D'Angelo 2017	USA, Europe, Israel, Australia/New Zealand	–	75 (48)	157	–	NR	–	–	NR	Range: 6.2–8.6 months	
		Nivolumab alone	42 (49)	86	61 (22–89)	NR	0–57 (66) 1–27 (31) ≥2–0 (0) NR–2 (2)	Locally advanced/nodal metastasis—28 (33) Distant metastasis—57 (66) NR—1 (1)	–	–	BRAF—4/83 (5)
		Ipilimumab + Nivolumab	16 (46)	35	65 (35–86)	NR	0–24 (69) 1–10 (29) ≥2–1 (3)	Locally advanced/nodal metastasis—12 (34) Distant metastasis—22 (63) NR—1 (3)	–	–	BRAF—3/35 (9)
		Ipilimumab alone	17 (47)	36	61 (31–80)	NR	0–25 (69) 1–11 (31) ≥2–0 (0)	Locally advanced/nodal metastasis—16 (44) Distant metastasis—19 (53) NR—1 (3)	–	–	BRAF—0/36 (0)
Rose 2021	Canada	–	12 (43)	28	<60	NR	NR	Locally advanced/nodal metastasis—4 (14) Distant metastasis—24 (86)	10 (36) with prior systemic therapy	20.5 months	BRAF—0 (0) NRAS—46 (25)
		Anti-PD1	–	15	–	–	–	–	–	–	–
		Anti-PD1 + anti-CTLA4	–	13	–	–	–	–	–	–	–
Dimitriou 2022	USA, Europe, Australia, Asia	–	187 (34)	545	65 (25–93)	Gastrointestinal—116 (21) Urogenital—178 (32) Head and neck—206 (38) Others—45 (8)	0–341 (64) ≥1–192 (35)	Locally advanced—136 (25) Metastatic—409 (75)	0–471 (86) 1-2–72 (13) ≥3–2 (1)	31 months (95% CI: 17–54)	BRAF—23 (4) KIT—52 (10) NRAS—50 (9)
		Anti-PD1	120 (33)	348	67 (27–97)	Gastrointestinal—70 (20) Urogenital—104 (30) Head and neck—140 (40) Others—34 (10)	0–215 (63) ≥1–128 (37)	Locally advanced—75 (22) Metastatic—273 (78)	0–288 (82) 1-2–59 (17) ≥3–1 (1)	–	BRAF—12 (10) KIT—21 (18) NRAS—27 (23)
		Anti-PD1 + ipilimumab	67 (33)	197	64 (31–85)	Gastrointestinal—46 (23) Urogenital—74 (38) Head and neck—66 (34) Other—11 (6)	0–126 (66) ≥1–64 (33)	Locally advanced—61 (31) Metastatic—136 (69)	0–193 (93) 1-2–13 (6) ≥3–1 (1)	–	BRAF—11 (6) KIT—31 (16) NRAS—23 (12)
Ho 2022	USA	–	15 (42)	36	62 (33–83)	Gastrointestinal—21 (59) Urogenital—9 (25) Head and neck—6 (17) Others—0 (0)	0-1–36 (100) ≥2–0 (0)	Locally advanced—19 (53) Metastatic—17 (47)	NR	37.9 months	BRAF—2/33 (6) KIT—10/33 (30) NRAS—4/33 (12)
		Anti-PD1 + anti-CTLA4	–	28	–	–	–	–	–	–	–
		Anti-PD1 only	–	7	–	–	–	–	–	–	–
		Anti-CTLA4 only	–	1	–	–	–	–	–	–	–
Shoushtari 2016	USA	Anti-PD1 (Pembrolizumab/Nivolumab)	11 (31)	35	65 (37–89)	Gastrointestinal—12 (34) Urogenital—14 (40) Head and neck—9 (26) Others—0 (0)	0–18 (51) ≥1–17 (49)	Locally advanced—1 (3) Metastatic—34 (97)	28 (80) with prior systemic therapy	10.6 months	BRAF—0/35 (0) KIT—2/35 (6) NRAS—4/35 (11)
Teterycz 2020	Europe	Anti-PD1 (Pembrolizumab/Nivolumab)	29 (35)	63	67.5 ([IQR] 57–76)	Gastrointestinal—27 (33) Urogenital—22 (27) Head and neck—33 (40) Others—0 (0)	0–40 (49) ≥1–42 (51)	Locally advanced—14 (17) Metastatic—68 (83)	NR	29.0 months (95% CI: 19.1–41.7)	BRAF—5/82 (6)
Uhara 2021	Asia	Nivolumab	NR^a^	6	NR^a^	NR	NR^a^	NR^a^	NR^a^	NR^a^	NR^a^
Yamazaki 2017	Asia	Pembrolizumab	NR^a^	8	NR^a^	NR	NR^a^	NR	NR^a^	NR^a^	NR^a^
Hamid 2018	USA, Europe, Israel, Australia	Pembrolizumab	36 (43)	84	64 (15–87)	NR	0–57 (68) 1–27 (32) ≥2–0 (0)	M1c disease—68 (81)	0–8 (10) 1–31 (37) 2–38 (45) ≥3–7 (8)	NR	BRAF—7/84 (8)
Nomura 2020	Asia	Nivolumab	13 (65)	20 (17 evaluated for efficacy)	66 (28–84)	Gastrointestinal—4 (20) Urogenital—3 (15) Head and neck—10 (50) Others—3 (15)	0–12 (60) 1–8 (40) ≥2–0 (0)	Locally advanced—4 (20) Metastatic—16 (80)	0–18 (90) 1–2 (10) 2–0 (0) ≥3–0 (0)	19.2 months (13.2–33.6)	BRAF—0/17 (0)
Ogata 2021	USA	Anti-PD1 (Pembrolizumab/Nivolumab)	21 (36)	59	69 (35–89)	Gastrointestinal—18 (31) Urogenital—17 (29) Head and neck—24 (40) Others—0 (0)	0–38 (64) ≥1–21 (36)	Locally advanced—6 (10) Metastatic—53 (90)	48 (81) with prior systemic therapy	16.5 months	BRAF—2/69 (3) KIT—6/69 (10) NRARS—11/69 (19)
Xue et al.	Australia, China, Japan, US	Anti-PD1	NR^a^	102	NR^a^	NR^a^	NR^a^	NR^a^	NR^a^	NR^a^	NR^a^
Jacques et a;	10 countries	Anti-PD1	14 (25.5)	55 (28 evaluated)	61	Gastrointestinal—12 (21.8) Urogenital—27 (49.1) Head and neck—16 (29.1) Others—0 (0)	NR	NR	NR	21 months (IQR 13.8–32)	NR
Kim 2019	Asia	–	10 (32)	31	55.1 ± 13.2	Gastrointestinal—8 (26) Urogenital—3 (10) Head and neck—20 (65) Others—0 (0)	NR	NR	NR	17.4 months (3.7–95.2)	BRAF—1/15 (7) KIT—0/9 (0)
		Pembrolizumab + RT	2 (17)	12	58.5 ± 15.1	Gastrointestinal—4 (33) Urogenital—3 (25) Head and neck—5 (42) Others—0 (0)	–	–	–	14 months (3.7–95.2)	BRAF—0/5 (0) KIT—0/4 (0)
		Pembrolizumab alone	3 (38)	8	59.5 ± 8.6	Gastrointestinal—1 (13) Urogenital—0 (0) Head and neck—7 (88) Others—0 (0)	–	–	–	9 months (4–35)	BRAF—0/6 (0) KIT—0/3 (0)
		RT alone	5 (46)	11	55.0 ± 12.2	Gastrointestinal—3 (27) Urogenital—0 (0) Head and neck—8 (73) Others—0 (0)	–	–	–	21.9 months (9.2–56.9)	BRAF—1/4 (25) KIT—0/2 (0)
Kiyohara 2018	Asia	Nivolumab	NR^a^	208 (185 evaluated for efficacy)	NR^a^	NR	NR^a^	NR	NR^a^	NR	NR
Nathan 2019	Europe	Nivolumab	23 (37)	63	63 (34–86)	NR	0-1–54 (86) ≥2–9 (14)	Locally advanced—0 (0) Metastatic—63 (100)	0–0 (0) 1–32 (51) 2–22 (35) ≥3–9 (14)	6.6 months	BRAF—6/60 (10)
Si 2022	Asia	Pembrolizumab	NR^a^	15	NR^a^	NR	NR^a^	NR^a^	NR	NR^a^	NR^a^
Li 2022	Asia	Axitinib + Toripalimab	12 (41)	29	54 (27–70)	Gastrointestinal—11 (38) Urogenital—7 (24) Head and neck—11 (38) Others—0 (0)	0–16 (55) 1–13 (45) ≥2–0 (0)	Locally advanced—6 (21) Metastatic—23 (79)	NR	42.5 months (1.5–43.7)	NR
Tang 2021	Asia	Axitinib + anti-PD1	58 (39)	147	61 (26–87)	Gastrointestinal—56 (38) Urogenital—27 (18) Head and neck—62 (42) Others—2 (1)	0–71 (48) 1–71 (48) ≥2–0 (0)	Locally advanced—41 (28) Metastatic—106 (72)	0–81 (55) 1–36 (25) 2–22 (15) ≥3–8 (5)	20.1 months (1.2–27.5)	BRAF—2/127 (2) KIT—8/127 (6) NRAS—14/127 (11)
Lian 2024	China	Axitinib + Toripalimab	8 (27.6)	29 (24 evaluated)	62 (34–72)	Gastrointestinal—15 (51.7) Urogenital—10 (34.5) Head and neck—4 (13.8) Others	0–3 (10.3) 1–26 (89.7)	Localized—9 (31.0) Regional lymphatic disease—19 (65.5) Metastatic disease—1 (3.5)	NR	34.2 months (95% CI: 20.4–48.0 months)	BRAF—2 (6.9) KIT—4 (13.8) NRAS—3 (10.3)
Zhao et al.	China	Apatinib + Camrelizumab	14 (43.8)	32 (28 evaluated)	62 (35–77)	Gastrointestinal—8 Urogenital—7 Head and neck—17 Others—0	0-1–32 (100)	NR	0–14 ≥1–14	18.07 months (IQR: 3.75–36.40)	BRAF—2/26 (7.7) KIT—3/26 (11.5) NRAS—8/26 (30.8)
Jung 2022	USA, Australia	Imatinib	NR^a^	25	NR^a^	NR	NR^a^	NR^a^	NR^a^	NR	BRAF—3/20 (15) KIT—25/25 (100) NRAS—1/20 (5)
Kalinsky 2017	USA	Dasatinib	NR^a^	29	NR^a^	NR	NR^a^	NR	NR	NR^a^	NR^a^

Abbreviations: Not reported, NR; Interquartile range, IQR.

Metastatic disease includes both nodal and distant metastases.

a Data for the study population was reported, but not specific to MM patients.

**Table 2:** Overall survival and progression-free survival for the different treatment types.

Intervention	Outcome	Number of studies	Number of patients	Median survival time/months	Survival at 12 months/%
Anti-CTLA4	PFS	4	139	3.04 (95% CI: 2.91, 3.93)	9.8 (95% CI: 5.9, 16.5)
Anti-CTLA4	OS	6	305	6.71 (95% CI: 6.03, 8.03)	33.3 (95% CI: 28.4, 39.1)
Anti-PD1 + Anti-CTLA4	PFS	7	401	4.67 (95% CI: 3.55, 5.99)	35.1 (95% CI: 30.5, 40.4)
Anti-PD1 + Anti-CTLA4	OS	9	476	25.9 (95% CI: 21.9, 31.7)	71.8 (95% CI: 67.6, 76.2)
Anti-PD1	PFS	15	1142	4.33 (95% CI: 3.96, 5.34)	28.3 (95% CI: 25.8, 31.2)
Anti-PD1	OS	17	1399	18.3 (95% CI: 16.9, 21.3)	64.0 (95% CI: 61.4, 66.7)
Anti-PD1 + VEGF inhibitors	OS	4	233	16.8 (95% CI: 12.6, 20)	57.1 (95% CI: 51.0, 63.9)
KIT inhibitors	PFS	2	71	3.75 (95% CI: 2.51, 4.87)	8.3 (95% CI: 3.7, 18.7)
KIT inhibitors	OS	2	70	11 (95% CI: 7.6, 18.9)	48.2 (95% CI: 37.6, 61.8)

**Table 3:** Overall survival and progression-free survival in double-arm studies.

Intervention 1	Intervention 2	Outcome	Number of studies	Number of patients (Intervention 1)	Number of patients (Intervention 2)	HR (95% CI)	P-value	Median survival time (Intervention 1)/months	Median survival time (Intervention 2)/months	Survival at 12 months (Intervention 1)/%	Survival at 12 months (Intervention 2)/%
Anti-PD1	Anti-PD1 + RT	OS	2	123	73	Anti-PD1 + RT versus Anti-PD1: 0.854 (95% CI: 0.567, 1.29)	0.451	19.2 (95% CI: 15.4, 27.6)	27.6 (95% CI: 18.8, 46.4)	65.9 (95% CI: 57.5, 75.4)	72.9 (95% CI: 62.8, 84.5)
Anti-PD1	Anti-PD1 + Anti-CTLA4	OS	4	741	318	Anti-PD1+ Anti-CTLA4 versus Anti-PD1: 0.856 (95% CI: 0.704, 1.04)	0.122	18.8 (95% CI: 17.0, 23.0)	24.9 (95% CI: 19.8, 28.9)	65.6 (95% CI: 62.1, 69.3)	69.6 (95% CI: 64.2, 75.4)
Anti-PD1	Anti-PD1 + RT	PFS	2	123	73	Anti-PD1 + RT versus Anti-PD1: 0.994 (95% CI: 0.710, 1.39)	0.972	5.87 (95% CI: 4.29, 8.77)	5.80 (95% CI: 3.94, 8.90)	33.5 (95% CI: 25.9, 43.3)	30.3 (95% CI: 21.2, 43.4)
Anti-PD1	Anti-PD1 + Anti-CTLA4	PFS	5	827	353	Anti-PD1 + CTLA4 versus Anti-PD1: 0.919 (95% CI: 0.788, 1.07)	0.283	5.34 (95% CI: 4.29, 5.95)	4.90 (95% CI: 3.69, 6.15)	31.4 (95% CI: 28.3, 34.8)	35.5 (95% CI: 30.6, 41.2)
Anti-PD1	Anti-CTLA4	PFS	2	60	106	Anti-PD1 versus CTLA4: 0.548 (95% CI: 0.376, 0.799)	0.00177	4.44 (95% CI: 2.57, 6.89)	2.80 (95% CI: 2.64, 3.02)	29.3 (95% CI: 21.2, 40.4)	6.9 (95% CI: 2.7, 17.8)

### Efficacy of anti-PD1 and anti-CTLA4 monotherapy

**Fig. 2:**
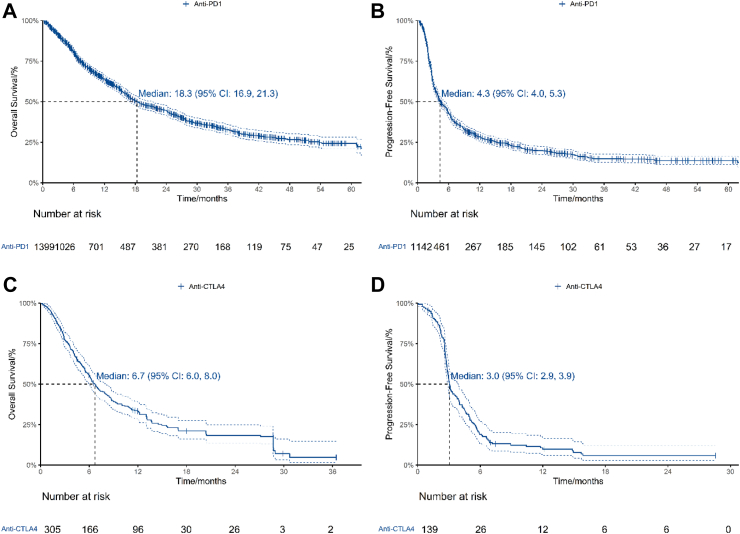
Efficacy of anti-PD1 and anti-CTLA4. 2A, OS of patients treated with anti-PD1. 2B, PFS of patients treated with anti-PD1. 2C, OS of patients treated with anti-CTLA4. 2D, PFS of patients treated with anti-CTLA4.

17 studies published OS data of 1399 patients treated with anti-PD1^24^^,^^30^, ^31^, ^32^, ^33^,^40^, ^41^, ^42^, ^43^, ^44^, ^45^^,^^64^^,^^67^^,^^68^^,^^70^^,^^73^^,^^74^ (Table 2, Fig. 2A). Median OS was 18.3 months (95% CI: 16.9, 21.3). 12-month OS was 64.0% (95% CI: 61.4%, 66.7%). 15 studies published PFS data of 1142 patients treated with anti-PD1^24^^,^^30^, ^31^, ^32^, ^33^^,^^38^^,^^40^^,^^42^, ^43^, ^44^, ^45^^,^^64^^,^^66^^,^^67^^,^^70^ (Table 2, Fig. 2B). Median PFS was 4.33 months (95% CI: 3.96, 5.34). 12-month PFS was 28.3% (95% CI: 25.8%, 31.2%).

Six studies published OS data of 305 patients treated with anti-CTLA4^26^^,^^28^^,^^42^^,^^62^, ^63^, ^64^ (Table 2, Fig. 2C). Median OS was 6.71 months (95% CI: 6.03, 8.03). 12-month OS was 33.3% (95% CI: 28.4%, 39.1%). Four studies published PFS data of 139 patients treated with anti-CTLA4^26^^,^^62^^,^^64^^,^^66^ (Table 2, Fig. 2D). Median PFS was 3.04 months (95% CI: 2.91, 3.93). 12-month PFS was 9.8% (95% CI: 5.9%, 16.5%).

### Efficacy of anti-PD1 versus anti-CTLA4 monotherapy

**Fig. 3:**
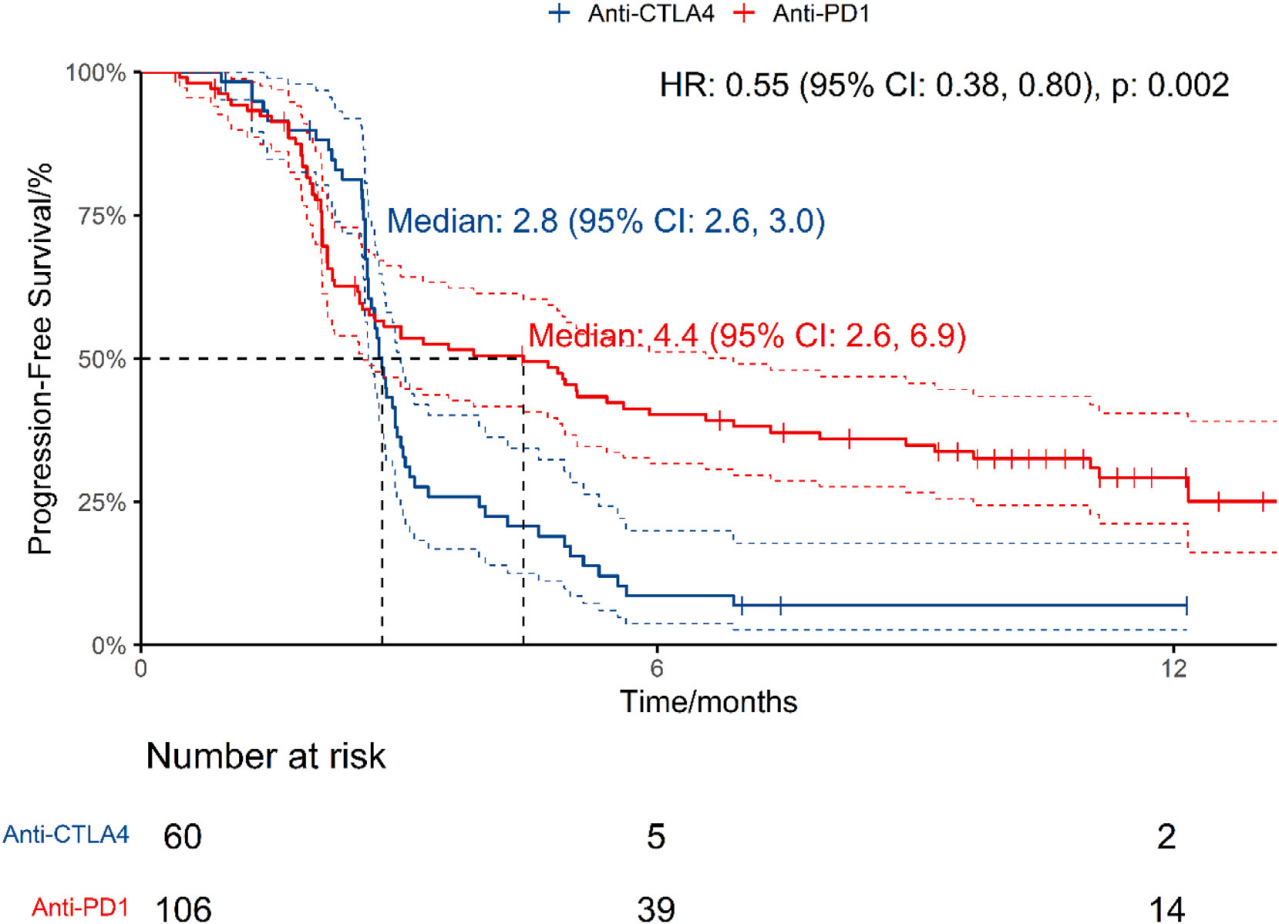
PFS of patients treated with anti-PD1 versus anti-CTLA4 in double-arm studies.

Two double-arm studies published PFS data of 166 patients comparing anti-PD1 against anti-CTLA4^64^^,^^66^ (Table 3, Fig. 3). The PFS HR was 0.548 (95% CI: 0.376, 0.799, P = 0.002), for a 45.2% reduction in progression risk with anti-PD1 versus anti-CTLA4. The PFS RMTL and RMST ratios of anti-PD1/anti-CTLA4 were 0.715 (95% CI: 0.606, 0.844, P < 0.001) and 1.659 (95% CI: 1.316, 2.092, P < 0.001) respectively (Table 4). The median PFS was 4.44 months (95% CI: 2.57, 6.89) for anti-PD1 and 2.80 months (95% CI: 2.64, 3.02) for anti-CTLA4. The estimated 12-month PFS was 29.3% (95% CI: 21.2%, 40.4%) for the anti-PD1 arm versus 6.9% (95% CI: 2.7%, 17.8%) for the anti-CTLA4 arm.

**Table 4:** Restricted mean survival time (RMST) and restricted mean time lost (RMTL) ratios in double-arm studies.

Intervention 1	Intervention 2	Outcome	Number of studies	Number of patients (Intervention 1)	Number of patients (Intervention 2)	RMTL ratio of intervention 2/intervention 1 (95% CI)	P-value	RMST ratio of intervention 2/intervention 1 (95% CI)	P-value
Anti-PD1	Anti-PD1 + RT	OS	2	123	73	0.855 (95% CI: 0.675, 1.083)	0.193	1.194 (95% CI: 0.928, 1.536)	0.168
Anti-PD1	Anti-PD1 + Anti-CTLA4	OS	4	741	318	0.932 (95% CI: 0.832, 1.044)	0.225	1.102 (95% CI: 0.948, 1.281)	0.204
Anti-PD1	Anti-PD1 + RT	PFS	2	123	73	1.006 (95% CI: 0.87, 1.162)	0.939	0.984 (95% CI: 0.658, 1.472)	0.939
Anti-PD1	Anti-PD1 + Anti-CTLA4	PFS	5	827	353	0.936 (95% CI: 0.866, 1.013)	0.100	1.21 (95% CI: 0.979, 1.496)	0.078
Anti-CTLA4	Anti-PD1	PFS	2	106	60	0.715 (95% CI: 0.606, 0.844)	<0.001	1.659 (95% CI: 1.316, 2.092)	<0.001

### Efficacy of anti-PD1 combined with anti-CTLA4

**Fig. 4:**
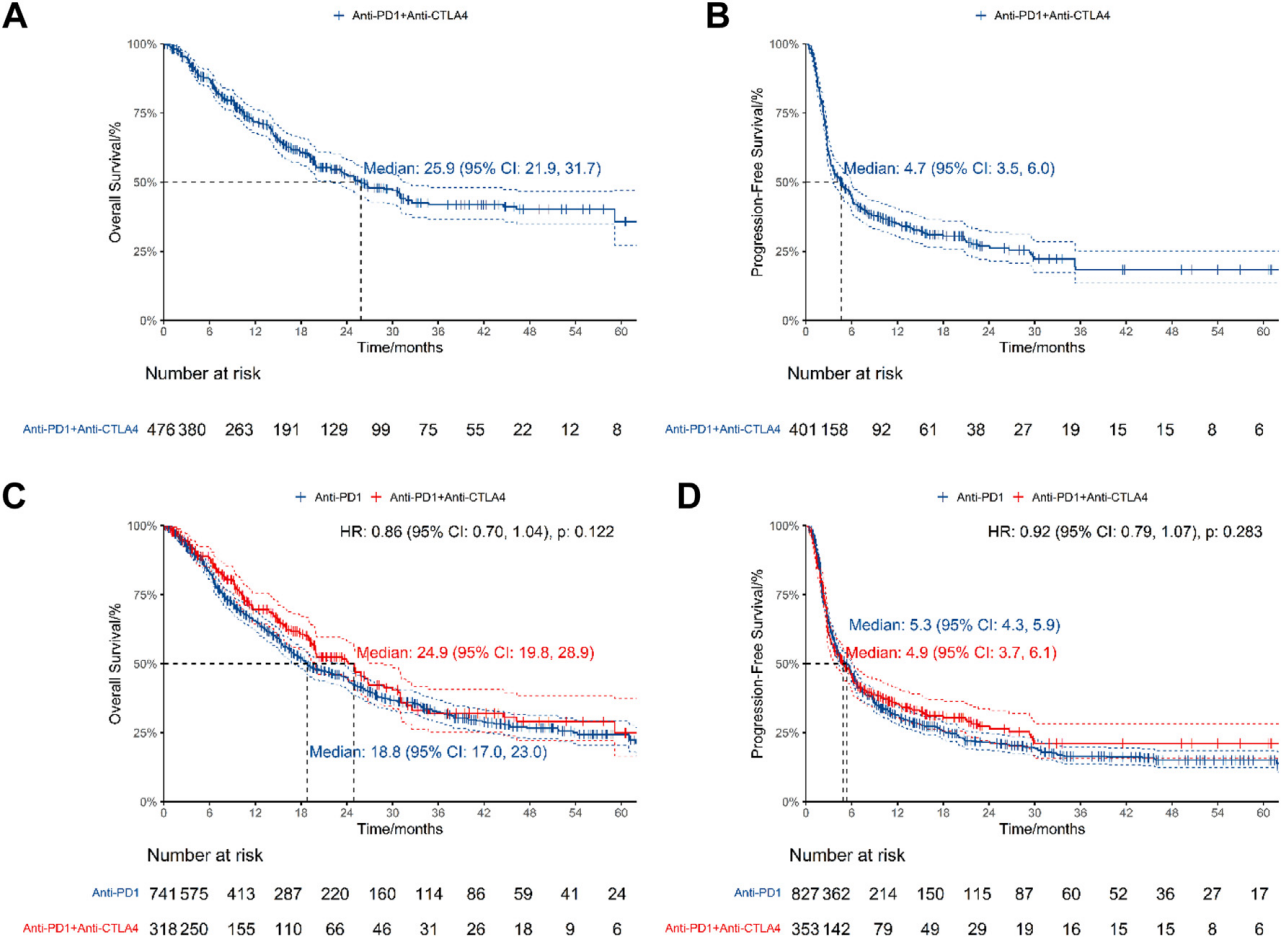
Efficacy of anti-PD1 combined with anti-CTLA4. 4A, OS of patients treated with anti-PD1 combined with anti-CTLA4. 4B, PFS of patients treated with anti-PD1 combined with anti-CTLA4. 4C, OS of patients treated with anti-PD1 versus anti-PD1 combined with anti-CTLA4 in double-arm studies. 4D, PFS of patients treated with anti-PD1 versus anti-PD1 combined with anti-CTLA4 in double-arm studies.

Nine studies published OS data of 476 patients treated with anti-CTLA4 and anti-PD1^29^, ^30^, ^31^, ^32^, ^33^,^37^^,^^65^^,^^71^^,^^72^ (Table 2, Fig. 4A). Median OS was 25.9 months (95% CI: 21.9, 31.7). 12-month OS was 71.8% (95% CI: 67.6%, 76.2%). Seven studies published PFS data of 401 patients treated with anti-CTLA4 and anti-PD1^30^, ^31^, ^32^, ^33^,^65^^,^^66^^,^^71^ (Table 2, Fig. 4B). Median PFS was 4.67 months (95% CI: 3.55, 5.99). 12-month PFS was 35.1% (95% CI: 30.5%, 40.4%).

### Double-arm analysis: Anti-PD1 monotherapy versus Anti-CTLA4 and Anti-PD1

Four double-arm studies published OS data of 1059 patients comparing anti-PD1 against anti-CTLA4 and anti-PD1^30^, ^31^, ^32^, ^33^ (Table 3, Fig. 4C). The OS HR was 0.856 (95% CI: 0.704, 1.04, P = 0.122) with anti-CTLA4 and anti-PD1 versus anti-PD1. The OS RMTL and RMST ratios of anti-CTLA4 and anti-PD1/anti-PD1 were 0.932 (95% CI: 0.832, 1.044, P = 0.225) and 1.102 (95% CI: 0.948, 1.281, P = 0.204) respectively (Table 4). The median OS was 18.8 months (95% CI: 17.0, 23.0) for anti-PD1 and 24.9 months (95% CI: 19.8, 28.9) for anti-CTLA4 and anti-PD1. The estimated 12-month OS was 65.6% (95% CI: 62.1%, 69.3%) for the anti-PD1 arm versus 69.6% (95% CI: 64.2%, 75.4%) for the anti-CTL4 and anti-PD1 arm.

Five double-arm studies published PFS data of 1180 patients comparing anti-CTLA4 and anti-PD1 against anti-PD1^30^, ^31^, ^32^, ^33^^,^^66^ (Table 3, Fig. 4D). The PFS HR was 0.919 (95% CI: 0.788, 1.07, P = 0.283) with anti-CTLA4 and anti-PD1 versus anti-PD1. The PFS RMTL and RMST ratios of anti-CTLA4 and anti-PD1/anti-PD1 were 0.936 (95% CI: 0.866, 1.013, P = 0.100) and 1.21 (95% CI: 0.979, 1.496, P = 0.078) respectively (Table 4). The median PFS was 5.34 months (95% CI: 4.29, 5.95) for anti-PD1 and 4.90 months (95% CI: 3.69, 6.15) for anti-CTLA4 and anti-PD1. The estimated 12-month PFS was 31.4% (95% CI: 28.3%, 34.8%) for the anti-PD1 arm versus 35.5% (95% CI: 30.6%, 41.2%) for the anti-CTLA4 and anti-PD1 arm.

### Efficacy of anti-PD1 combined with other modalities

**Fig. 5:**
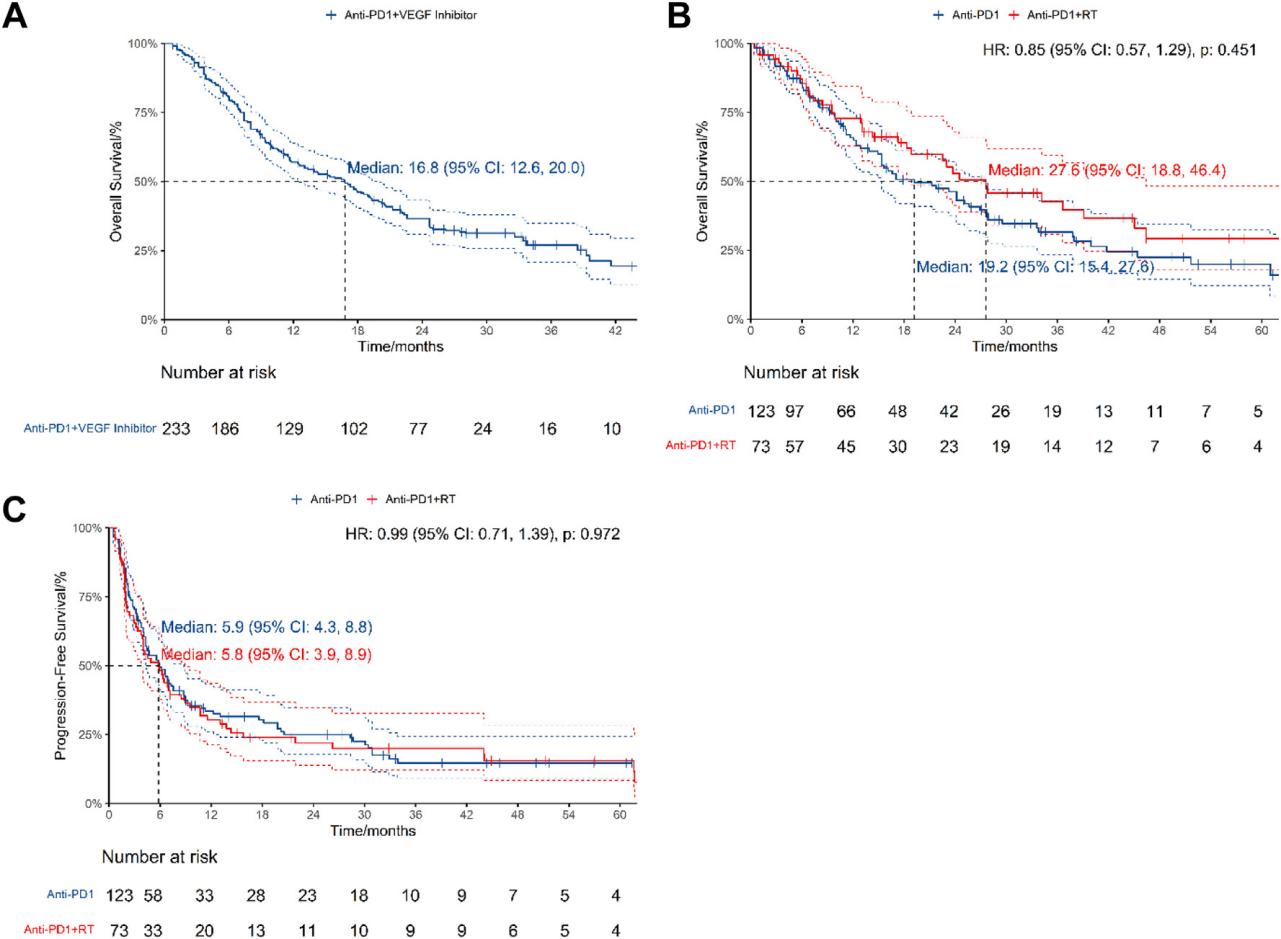
Efficacy of anti-PD1 combined with other modalities. 5A, OS of patients treated with anti-PD1 combined with VEGF inhibitors. 5B, OS of patients treated with anti-PD1 versus anti-PD1 combined with RT in double-arm studies. 5C, PFS of patients treated with anti-PD1 versus anti-PD1 combined with RT in double-arm studies.

Four studies published OS data of 233 patients treated with anti-PD1 and VEGF inhibitors^34^^,^^35^^,^^70^^,^^75^ (Table 2, Fig. 5A). Median OS was 16.8 months (95% CI: 12.6, 20). 12-month OS was 57.1% (95% CI: 51.0%, 63.9%). No studies reported PFS.

Two double-arm studies published OS data of 196 patients comparing anti-PD1 against anti-PD1 and radiotherapy (RT)^24^^,^^30^ (Table 3, Fig. 5B). The OS HR was 0.854 (95% CI: 0.567, 1.29, P = 0.451) with anti-PD1 and RT versus anti-PD1. The OS RMTL and RMST ratios of anti-PD1 and RT/anti-PD1 were 0.855 (95% CI: 0.675, 1.083, P = 0.193) and 1.194 (95% CI: 0.928, 1.536, P = 0.168) respectively (Table 4). The median OS was 19.2 months (95% CI: 15.4, 27.6) for anti-PD1 and 27.6 months (95% CI: 18.8, 46.4) for anti-PD1 and RT. The estimated 12-month OS was 65.9% (95% CI: 57.5%, 75.4%) for the anti-PD1 arm versus 72.9% (95% CI: 62.8%, 84.5%) for the anti-PD1 and RT arm.

Two double-arm studies published PFS data of 196 patients comparing anti-PD1 against anti-PD1 and RT^24^^,^^30^ (Table 3, Fig. 5C). The PFS HR was 0.994 (95% CI: 0.710, 1.39, P = 0.972) with anti-PD1 and RT versus anti-PD1. The PFS RMTL and RMST ratios of anti-PD1 and RT/anti-PD1 were 1.006 (95% CI: 0.87, 1.162, P = 0.939) and 0.984 (95% CI: 0.658, 1.472, P = 0.939) respectively (Table 4). The median PFS was 5.87 months (95% CI: 4.29, 8.77) for anti-PD1 and 5.80 months (95% CI: 3.94, 8.90) for anti-PD1 and RT. The estimated 12-month PFS was 33.5% (95% CI: 25.9%, 43.3%) for the anti-PD1 arm versus 30.3% (95% CI: 21.2%, 43.4%) for the anti-PD1 and RT arm.

### Efficacy of KIT inhibitors

Two studies published OS data of 70 patients treated with KIT inhibitors^36^^,^^39^ (Table 2, Supplementary Figure S1A). Median OS was 11 months (95% CI: 7.6, 18.9). 12-month OS was 48.2% (95% CI: 37.6%, 61.8%). Two studies published PFS data of 71 patients treated with KIT inhibitors^36^^,^^39^ (Table 2, Supplementary Figure S1B). Median PFS was 3.75 months (95% CI: 2.51, 4.87). 12-month PFS was 8.3% (95% CI: 3.7%, 18.7%).

### Overall survival and progression-free survival for all included studies

To further illustrate the efficacy of the different treatment modalities, the OS and PFS of the overall population and the different treatment types are illustrated in Supplementary Figures S2 and S3.

### Two-stage meta-analysis of 12-month survival proportion and HRs

The sensitivity analysis of 12-month survival proportion and HRs can be found in Supplementary Tables S2 and S3, Supplementary Figures S4 and S5. The results of this sensitivity analysis are largely concordant with the one-stage meta-analysis. For the analysis of the survival proportions of different treatment types, most of the results were low to moderate in heterogeneity. Anti-PD1 and anti-PD1 combined with VEGF inhibitors had the highest heterogeneity with I^2^ being more than 75%, while anti-CTLA4 had the lowest heterogeneity (0.0% for PFS and 22.6% for OS). For the pooled hazard ratios in double arm studies, most analyses had low to moderate heterogeneity. The analysis comparing anti-PD1 against anti-PD1 and anti-CTLA4 had the highest heterogeneity, with an I^2^ of 71.5%. Possible sources of heterogeneity are discussed below.

### Risk of bias

Across the domains of selection bias, performance bias, detection bias, attrition bias, reporting bias, and other bias, the studies were all low in bias (Supplementary Table S4).

## Discussion

To our knowledge, this is the first individual patient data meta-analysis investigating the efficacy of immunotherapy and other treatments on survival outcomes in MM. At present, systemic treatment options in MM follow cutaneous melanoma guidelines closely, as the rarity of the disease poses a challenge to developing evidence-based clinical guidelines for MM. Yet, it must be recognized that cutaneous melanomas and MM are two distinct entities, differing in pathogenesis, etiology and prognosis.^76^ While the pathogenesis of MM has not been fully elucidated, certain mutational profiles seem to be characteristic—c-KIT tends to be found in increased frequency in MM whereas BRAF mutations are rare; the converse is true in cutaneous melanomas.^25^ MM tend to be diagnosed later than cutaneous melanomas. Locoregional nodal metastasis at presentation is also more frequent in MM, possibly owing to less noticeable sites of disease and the rich lymphovascular supply of mucosal surfaces that aids metastasis.^77^ Compared to cutaneous melanomas, outcomes for MM are poorer irrespective of stage at diagnosis.^78^ There is therefore a need for an updated analysis of current data to guide the creation of an optimized set of guidelines for the management of MM.

Our study included a range of immunotherapy and targeted therapy agents: anti-PD1, anti-CTLA4, KIT inhibitors and VEGF inhibitors. Anti-PD1 and anti-CTLA4 therapies have long been established in the management of melanomas, with multiple landmark studies such as the CheckMate and KEYNOTE studies.^79^, ^80^, ^81^, ^82^, ^83^, ^84^ In our analyses, anti-PD1 demonstrated a clear survival benefit over anti-CTLA4 therapy. Anti-PD1 significantly improved PFS compared to anti-CTLA4 (HR: 0.548 [95% CI: 0.376, 0.799]). While the difference between these outcomes have not been elucidated mechanistically, our results are consistent with several keynote trials across various melanoma subtypes. A meta-analysis by Yun et al. performed on advanced melanoma patients showed that while both anti-CTLA4 and anti-PD1 treatments were associated with higher PFS rates when each treatment was compared to control, an indirect comparison of these two agents showed superior 6-month PFS of 28.5% versus 17.7% and ORR of 29.6% versus 17.7% in anti-PD1 compared to anti-CTLA4 treatment.^85^ Similar outcomes were also seen in the KEYNOTE-006 trial, in which 6-month PFS was 47.3% in the pembrolizumab group versus 26.5% in the ipilimumab group, and the CheckMate 067 clinical trial in melanomas, in which the median PFS was 6.9 months in the nivolumab group versus 2.9 months in the ipilimumab group. The anti-PD1 groups in these studies also did better in OS and rates of response.^80^^,^^86^

The combination of PD1 and CTLA4 blockers in cancer treatment has been suggested to augment the activation of anti-tumor immune response to improve outcomes in patients.^87^ It is theorized that blocking PD1, involved in the inhibition of effector T-cell and NK cell activation in peripheral tissues and in the induction of Treg cell differentiation, would function synergistically with inhibiting CTLA4, which is involved in the regulation of T-cell activation and in Treg-mediated immunosuppression.^88^^,^^89^ Multiple clinical trials of melanomas in general have demonstrated that combination therapy had higher response rates, and better PFS and OS.^86^^,^^90^, ^91^, ^92^, ^93^ However, the efficacy of combination therapy in MM may be more modest than the reported benefits in other subtypes of melanomas. For instance, a pooled analysis of data from patients treated with nivolumab alone or in combination with ipilimumab demonstrated that cutaneous melanoma patients receiving combination therapy had a median PFS of 11.7 months versus 6.2 months for nivolumab alone, while MM patients in the combination group had a median PFS of 5.9 months compared to 3.0 months for monotherapy.^66^ In our analysis of double-arm studies comparing combination anti-PD1 and anti-CTLA4 therapy with anti-PD1 therapy alone, while combination therapy did show numerically higher PFS (HR: 0.919 [0.788, 1.07]), OS (HR: 0.856 [0.704, 1.04]), superior RMST and RMTL ratios, these results did not reach statistical significance. This could also be due to the relatively small number of double-arm studies comparing monotherapy against a combined regimen of immunotherapies, warranting further study into the efficacy of combination therapy in MM. Nevertheless, given the non-significant difference between outcomes of combination therapy versus anti-PD1 monotherapy, it may be prudent to consider anti-PD1 monotherapy as the first-line treatment of choice for MM, when weighing between the benefits and risks of combination therapy. Asides from increased monetary costs to the patient, combination therapy with anti-CTLA4 carries an increased incidence of adverse effects. In their study, D'Angelo et al. demonstrated that the incidence of grade 3 or 4 treatment-related adverse events was only 8% for nivolumab monotherapy compared to 40% for combination therapy (nivolumab with ipilimumab).^66^ Similarly, Nakamura et al. reported a higher rate of ≥grade 3 immune-related adverse events in patients treated with combination anti-PD1 and anti-CTLA4 therapy versus anti-PD1 monotherapy (53% versus 17%).^31^

The use of radiotherapy to achieve local control, or as an “immune adjuvant” in MM has been discussed.^94^ Our data showed that combination of anti-PD1 therapy and radiotherapy did not show significant improvement in PFS (HR: 0.994 [0.710, 1.39]) and OS (HR: 0.854 [0.567, 1.29]) compared to anti-PD1 therapy alone. This is congruent with a study by Owens et al., which demonstrated that addition of radiotherapy did not significantly improve survival in head and neck MM, though it tended to decrease the rate of local failure.^95^

More recently, Nassar et al. have elucidated the differences in mutational profiles between MM and cutaneous melanomas.^96^ SF3B1 and KIT have been shown to have a higher mutation rate in MM as compared to cutaneous melanoma, whereas BRAF and NRAS have a lower mutation rate in MM.^96^ A study performed by Steeb et al. on the implications of c-Kit inhibitors on response rates for unresectable or metastatic MM, acral and sun-damaged melanomas demonstrated that objective responses were almost exclusively achieved by patients harboring KIT mutations in exon 11 and exon 13.^97^ For MM, the ORR was 0.14 (95% CI: 0.06, 0.24),^97^ while a meta-analysis of survival outcomes was not performed. In our study, we included two single-arm studies with KIT inhibitors.^36^^,^^39^ The pooled 12-month PFS was 8.3% (95% CI: 3.7, 18.7), while the pooled 12-month OS was 48.2% (95% CI: 37.6, 61.8). This was not superior to anti-PD1 alone (12-month PFS and OS 28.3% and 64.0% respectively), or anti-PD1 combination therapies. However, we lacked sufficient data to evaluate the efficacy of KIT inhibitors in KIT-mutated MM on survival outcomes, which is a key area for future research. Given the higher prevalence of KIT mutation in MM, it would be interesting to explore the combination of anti-PD1 therapy with KIT inhibitors, as previously suggested by Kim et al.^98^ A case report of combination therapy with pembrolizumab and imatinib in metastatic double KIT mutant melanoma showed good response with complete remission 6 months after treatment and no evidence of disease for almost 12 months.^99^ Another study also demonstrated partial remission of a metastatic c-KIT mutant MM with avapritinib treatment, after initial treatment failure on ipilimumab and nivolumab combination therapy.^100^ Despite these promising results, however, literature on this combined therapy in MM remains limited. Further cohort studies and randomized controlled trials on the combination of anti-PD1 and KIT inhibitors for KIT-mutant MM would therefore be beneficial.

While the majority of studies on immune checkpoint inhibitors in MM has been largely focused on adjuvant therapy, neoadjuvant therapy could also be a promising avenue of research. The definitive intervention for resectable MM is surgery, but surgical intervention is often limited by advanced and invasive disease in areas with challenging surgical access at the time of disease presentation. Neoadjuvant therapy could potentially downstage the tumour, making it amenable to resection. Emerging literature has suggested reasonable benefit of neoadjuvant immunotherapy in MM - a recent study on the utility of neoadjuvant toripalimab (anti-PD1) in combination with axitinib by Lian et al. earlier this year showed a favourable pathological response rate of 33.3% in a cohort of 29 patients. The team postulated that this could be because neoadjuvant immunotherapy positively modulates the tumour environment.^35^ Separately, Ho et al. reported a 3-year OS rate of 55% in patients receiving neoadjuvant immunotherapy (either anti-PD1, anti-CTLA4 or a combination).^37^ Validation of these results in larger studies for better patient selection should be encouraged.

Interestingly, our analyses revealed that there was some heterogeneity in the treatment effects across the included studies. For example, among the double-arm studies comparing between anti-PD1 and anti-CTLA4 combination therapy and anti-PD1 monotherapy, Rose et al. demonstrated the largest effect sizes for both OS and PFS (Supplementary Figures S4 and S5), with study participants receiving combination therapy having a significantly higher OS than anti-PD1 monotherapy (P = 0.0019).^32^ One possibility could be the relatively smaller number of MM participants in their study. In addition, compared to the other double arm studies included in our analysis, there was a higher proportion of participants with metastatic disease included in their study. Further research may be warranted to investigate the utility of combination therapy in metastatic versus locally advanced disease in MM.

There are several limitations of our review. Given the rarity of MM and its poor prognosis, many of the studies included were performed retrospectively and had relatively small sample sizes. The studies also spanned across differing patient baseline characteristics and locations of MM; however, the data was not sufficiently granular to perform subgroup analysis of specific cohorts such as those with tumor mutations or different melanoma locations. We were missing individual patient records and therefore utilized reconstruction for IPD analysis. Next, there was still relatively few studies, all studies were observational studies and not randomized clinical trials that reported on the efficacy of immunotherapy, targeted therapy, and radiotherapy for MM. This might incur bias to the individual study-level estimates as well as the summary estimates. The relatively heterogenous baseline characteristics of the included studies, coupled with the small number of double-arm studies making direct comparisons between treatment modalities made it untenable to perform a network meta-analysis. Moreover, in our analyses of double-arm studies, the assumption of proportional hazards was violated for some of the pairwise comparisons. The hazard ratios should hence be interpreted judiciously, while taking into account the RMTL and RMST ratios, along with the median survival times. In our study, the directionality and significance of the derived RMTL and RMST ratios are concordant with the hazard ratios.

In conclusion, this is the first individual patient data meta-analysis on the efficacy of immunotherapy and targeted therapy on survival outcomes in MM. Overall, our data suggests that for the systemic treatment of MM, anti-PD1 is the best monotherapy. While combining anti-PD1 with other treatment options such as anti-CTLA4, VEGF inhibitors or radiotherapy might achieve better outcomes, these improvements did not reach statistical significance when evaluated by HR, RMTL and RMST ratios. The efficacy of KIT inhibitors was also considerably lower than anti-PD1 monotherapy. Moving forward, it would be prudent to investigate the implications of KIT mutations on the efficacy of KIT inhibitor therapy, as well as the predictive impact of PD-L1 expression and other biomarkers on anti-PD1 treatments. In order to achieve greater insight into treatment regimens for MM (for instance, monotherapy versus combination therapy; adjuvant versus neoadjuvant therapy), international collaboration for the validation of these results in larger studies and randomized controlled trials should be encouraged.

## Contributors

VSY conceived and designed the study. AYTT, JVP, JYXN and TKS were involved in screening and reviewing potentially eligible studies, and retrieving data from the studies included. CEY and CEL analysed and interpreted the available data. AYTT, CEY and CEL were major contributors in writing the manuscript. VSY and JYTL provided critical feedback to the manuscript and data interpretation. All authors had access to and verified the data used in this study and had final responsibility for the decision to submit for publication. All authors read and approved the final manuscript.

## Data sharing statement

The datasets used and/or analysed during the current study are available from the corresponding author on reasonable request.

## Declaration of interests

The authors declare that they have no competing interests.
