# Supplementary material for: Effectiveness of immune checkpoint inhibitors and other treatment modalities in patients with advanced mucosal melanomas: a systematic review and individual patient data meta-analysis
Source: eClinicalMedicine. 2024 Oct 4;77:102870. doi: 10.1016/j.eclinm.2024.102870 (PMC11474374; doi:10.1016/j.eclinm.2024.102870)
Supplement: Supplementary material [file mmc1.docx]

**Supplementary Material**

**Supplementary Table 1: Graphs of scaled Schoenfeld residuals for double-arm studies**

| Intervention 1 | Intervention 2 | Outcome | Graph of the scaled Schoenfeld residuals |
| --- | --- | --- | --- |
| Anti-PD1 | Anti-PD1+RT | OS | 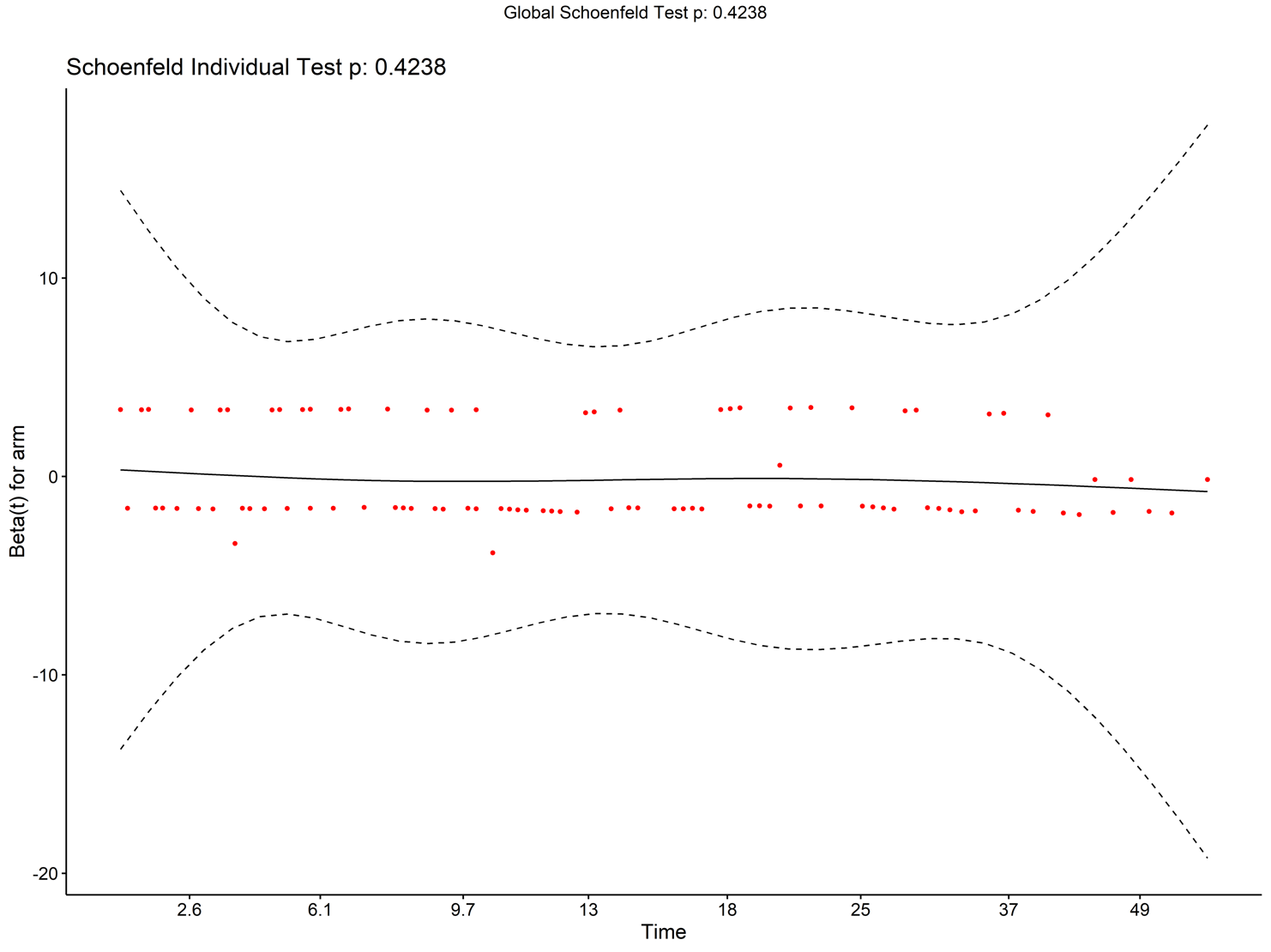 |
| Anti-PD1 | Anti-PD1+Anti-CTLA4 | OS | 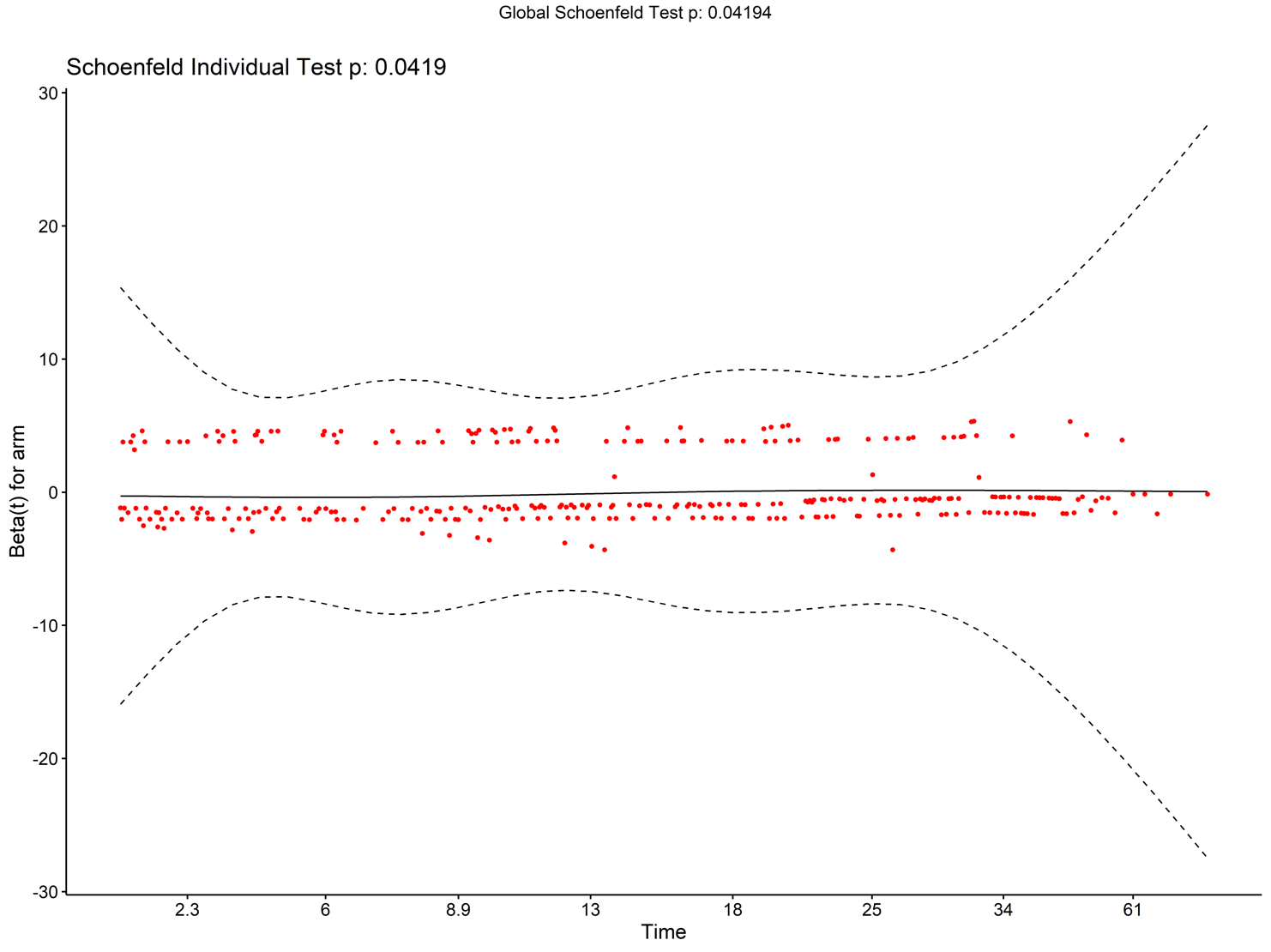 |
| Anti-PD1 | Anti-PD1+RT | PFS | 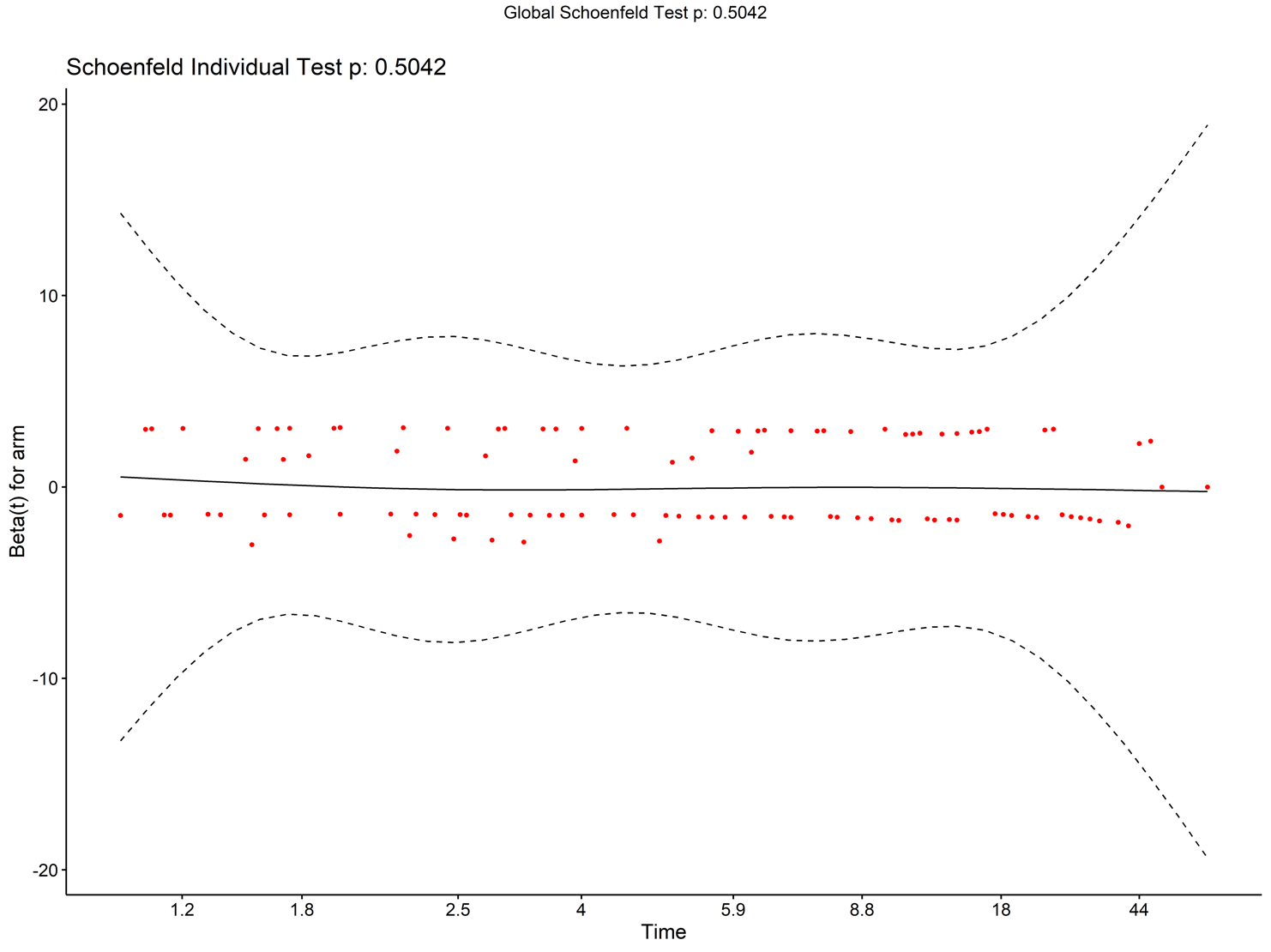 |
| Anti-PD1 | Anti-PD1+Anti-CTLA4 | PFS | 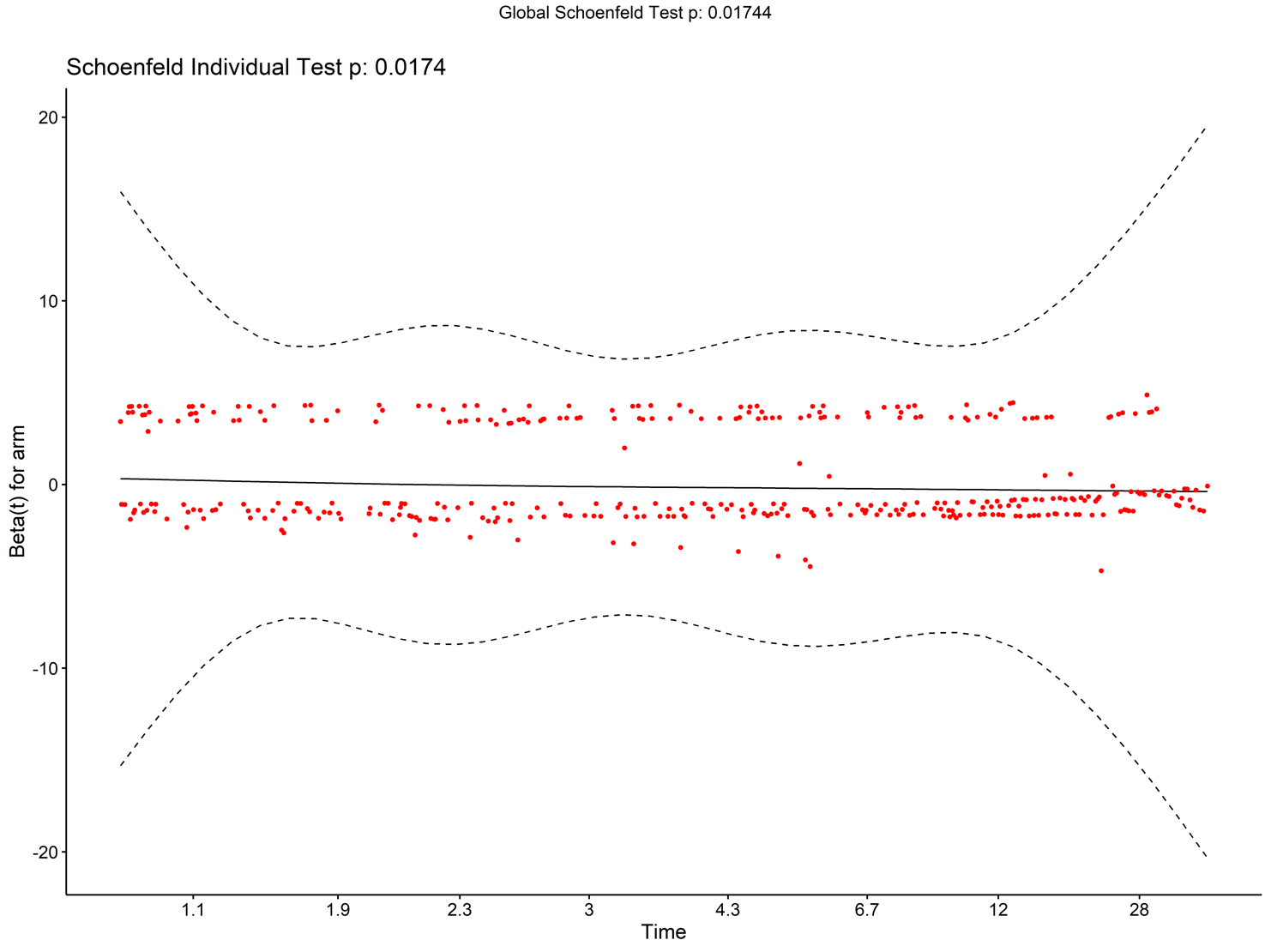 |
| Anti-PD1 | Anti-CTLA4 | PFS | 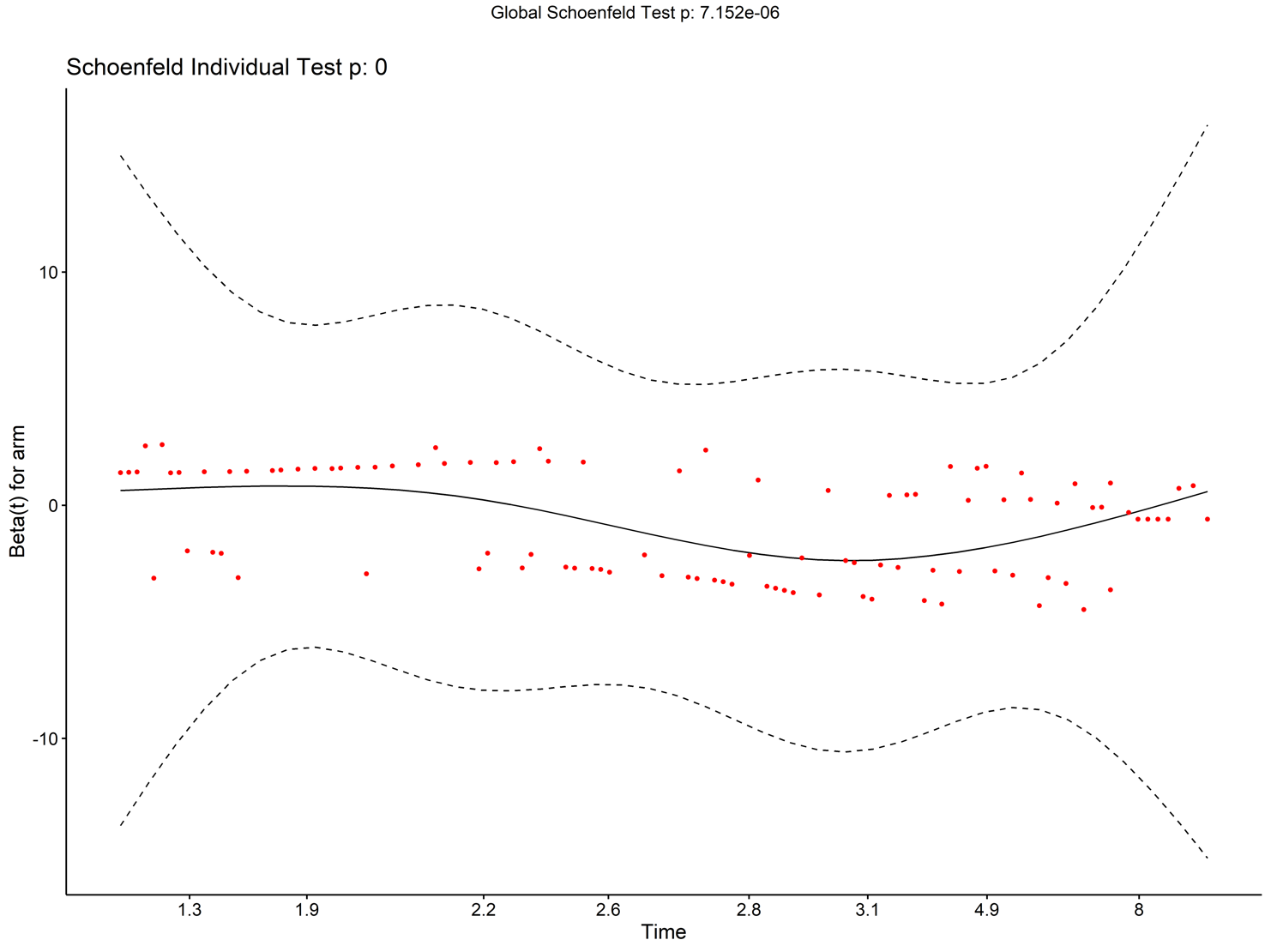 |

**Supplementary Table 2: Overall survival and progression-free survival for the different treatment types (two-stage meta-analysis)**

| **Intervention** | **Outcome** | **Survival at 12 months/%** | **I^2^** |
| --- | --- | --- | --- |
| Anti-CTLA4 | PFS | 12.6 (7.3, 20.9) | 0.0% |
| Anti-CTLA4 | OS | 33.8 (28.6, 39.3) | 22.6% |
| Anti-PD1+ Anti-CTLA4 | PFS | 37.0 (28.0, 46.9) | 50.7% |
| Anti-PD1+ Anti-CTLA4 | OS | 72.0 (63.7, 79.0) | 32.0% |
| Anti-PD1 | PFS | 24.2 (18.1, 31.6) | 55.8% |
| Anti-PD1 | OS | 61.8 (51.4, 71.3) | 79.7% |
| Anti-PD1+VEGF inhibitors | OS | 69.9 (26.5. 93.7) | 75.8% |
| KIT inhibitors | OS | 48.6 (37.2, 60.2) | 87.9% |

**Supplementary Table 3: Pooled hazard ratio in double-arm studies (two-stage meta-analysis)**

| **Intervention 1** | **Intervention 2** | **Outcome** | **Pooled HR** | **I^2^** |
| --- | --- | --- | --- | --- |
| PD1 | PD1+RT | OS | 0.857 (0.571, 1.28) | 56.4% |
| PD1 | PD1+CTLA4 | OS | 0.862 (0.709, 1.05) | 55.9% |
| PD1 | PD1+RT | PFS | 0.993 (0.707, 1.39) | 0.0% |
| PD1 | PD1+CTLA4 | PFS | 0.928 (0.796, 1.08) | 71.5% |
| PD1 | CTLA4 | PFS | 0.549 (0.375, 0.805) | 11.0% |

**Supplementary Table 4: Quality assessment of included cohort studies using the Joanna Brigg’s Institute Critical Appraisal tool**

|  | 1 | 2 | 3 | 4 | 5 | 6 | 7 | 8 | 9 | 10 | 11 |
| --- | --- | --- | --- | --- | --- | --- | --- | --- | --- | --- | --- |
| Alexander 2014 | NA | NA | Y | Y | Y | Y | Y | Y | Y | U | Y |
| Del Vecchio 2014 | NA | NA | Y | Y | Y | Y | Y | Y | Y | U | Y |
| Yamazaki 2020 | NA | NA | Y | Y | Y | Y | Y | Y | Y | Y | Y |
| Postow 2013 | NA | NA | Y | Y | Y | Y | Y | Y | Y | Y | Y |
| Zimmer 2015 | NA | NA | Y | Y | Y | Y | Y | Y | Y | Y | Y |
| Moya-Plana 2019 | Y | Y | Y | Y | U | Y | Y | Y | U | U | Y |
| Namikawa 2018 | NA | NA | Y | Y | U | Y | Y | Y | Y | Y | Y |
| Hodi 2021 | NA | NA | Y | Y | U | Y | Y | Y | Y | Y | Y |
| Kottschade 2023 | NA | NA | Y | Y | Y | Y | Y | Y | Y | Y | Y |
| Takahashi 2023 | NA | NA | Y | Y | Y | Y | Y | Y | Y | Y | Y |
| Umeda 2021 | Y | Y | Y | Y | Y | Y | Y | U | Y | Y | Y |
| Nakamura 2021 | Y | Y | Y | Y | Y | Y | Y | Y | Y | Y | Y |
| D'Angelo 2017 | Y | Y | Y | Y | Y | Y | Y | Y | U | Y | Y |
| Rose 2021 | Y | Y | Y | Y | Y | Y | Y | Y | U | Y | Y |
| Dimitriou 2022 | Y | Y | Y | Y | Y | Y | Y | Y | Y | Y | Y |
| Ho 2022 | NA | NA | Y | Y | Y | Y | Y | U | Y | Y | Y |
| Shoushtari 2016 | NA | NA | Y | Y | Y | Y | Y | Y | Y | Y | Y |
| Teterycz 2020 | NA | NA | U | Y | Y | Y | Y | Y | U | Y | Y |
| Uhara 2021 | NA | NA | Y | Y | Y | Y | Y | Y | Y | Y | Y |
| Yamazaki 2017 | NA | NA | Y | Y | U | Y | Y | Y | Y | Y | Y |
| Hamid 2018 | NA | NA | Y | Y | Y | Y | Y | Y | U | Y | Y |
| Nomura 2020 | NA | NA | Y | Y | U | Y | Y | Y | Y | Y | Y |
| Ogata 2021 | NA | NA | Y | Y | Y | Y | Y | Y | U | Y | Y |
| Xue 2024 | NA | NA | Y | Y | Y | Y | Y | Y | Y | Y | Y |
| Jacques 2024 | /na | NA | Y | Y | Y | Y | Y | Y | Y | Y | Y |
| Kim 2019 | Y | Y | Y | Y | Y | Y | Y | Y | Y | Y | Y |
| Kiyohara 2018 | NA | NA | Y | Y | Y | Y | Y | Y | Y | Y | Y |
| Nathan 2019 | NA | NA | Y | Y | U | Y | Y | Y | Y | Y | Y |
| Si 2022 | NA | NA | Y | Y | Y | Y | Y | Y | U | Y | Y |
| Li 2022 | NA | NA | Y | Y | Y | Y | Y | Y | Y | Y | Y |
| Tang 2021 | NA | NA | Y | Y | Y | Y | Y | Y | Y | Y | Y |
| Lian 2024 | NA | NA | Y | Y | Y | Y | Y | Y | Y | Y | Y |
| Zhao 2024 | NA | NA | Y | Y | Y | Y | Y | Y | Y | Y | Y |
| Jung 2022 | NA | NA | Y | Y | Y | Y | Y | Y | U | Y | Y |
| Kalinsky 2017 | Y | Y | Y | Y | U | Y | Y | Y | U | Y | Y |

| Checklist |
| --- |
| 1. Were the two groups similar and recruited from the same population? |
| 2. Were the exposures measured similarly to assign people to both exposed and unexposed groups? |
| 3. Was the exposure measured in a valid and reliable way? |
| 4. Were confounding factors identified? |
| 5. Were strategies to deal with confounding factors stated? |
| 6. Were the groups/participants free of the outcome at the start of the study (or at the moment of exposure)? |
| 7. Were the outcomes measured in a valid and reliable way? |
| 8. Was the follow up time reported and sufficient to be long enough for outcomes to occur? |
| 9. Was follow up complete, and if not, were the reasons to loss to follow up described and explored? |
| 10. Were strategies to address incomplete follow up utilized? |
| 11. Was appropriate statistical analysis used? |

Legend:

Y – Yes

N – No

U – Unclear

NA – Not applicable

**Supplementary Figure 1: Efficacy of KIT inhibitors. Supplementary Figure 1A, OS of patients treated with KIT inhibitors. Supplementary Figure 1B, PFS of patients treated with KIT inhibitors.**


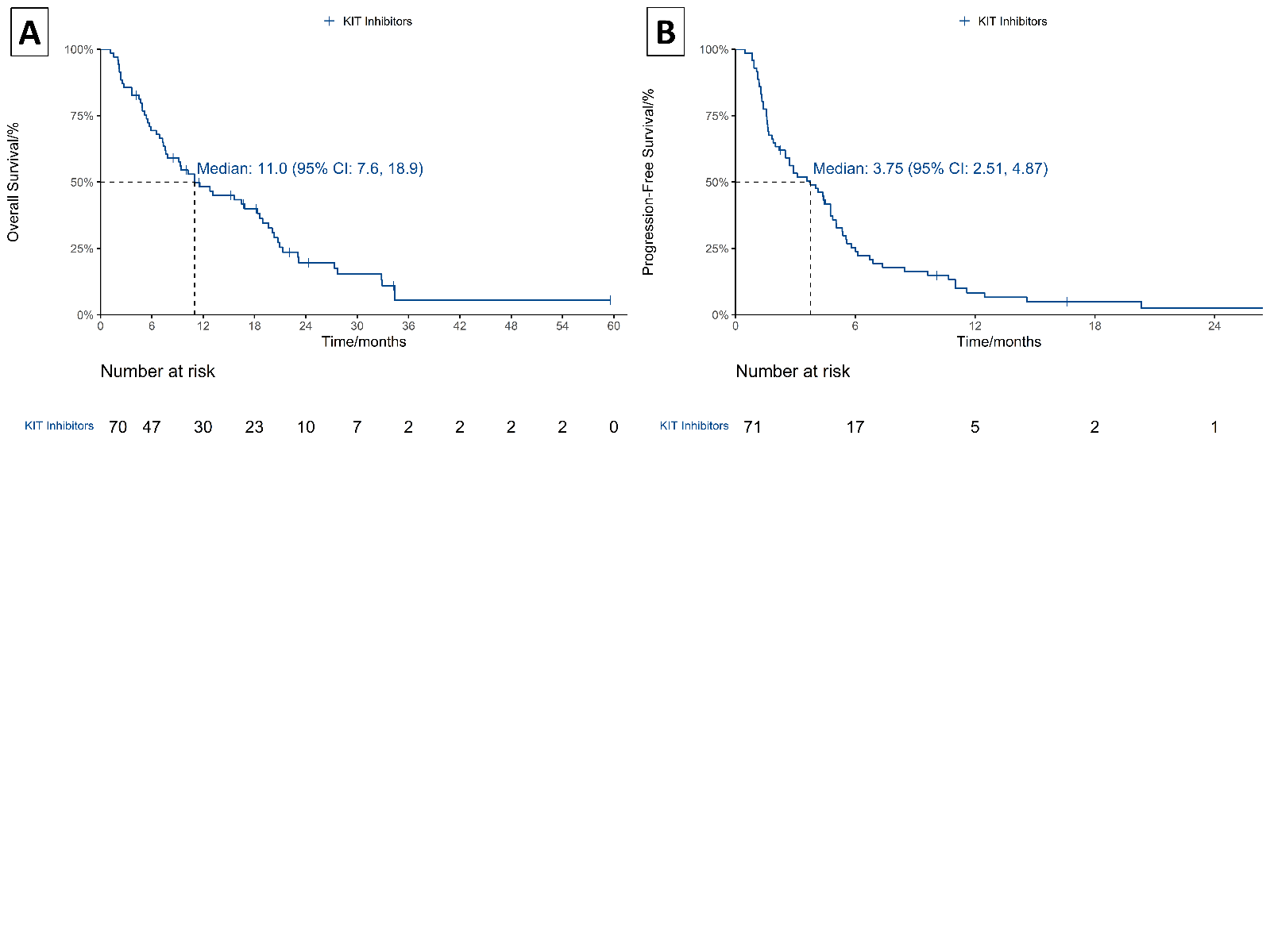


**Supplementary Figure 2: OS of overall population stratified by treatment modalities**

**
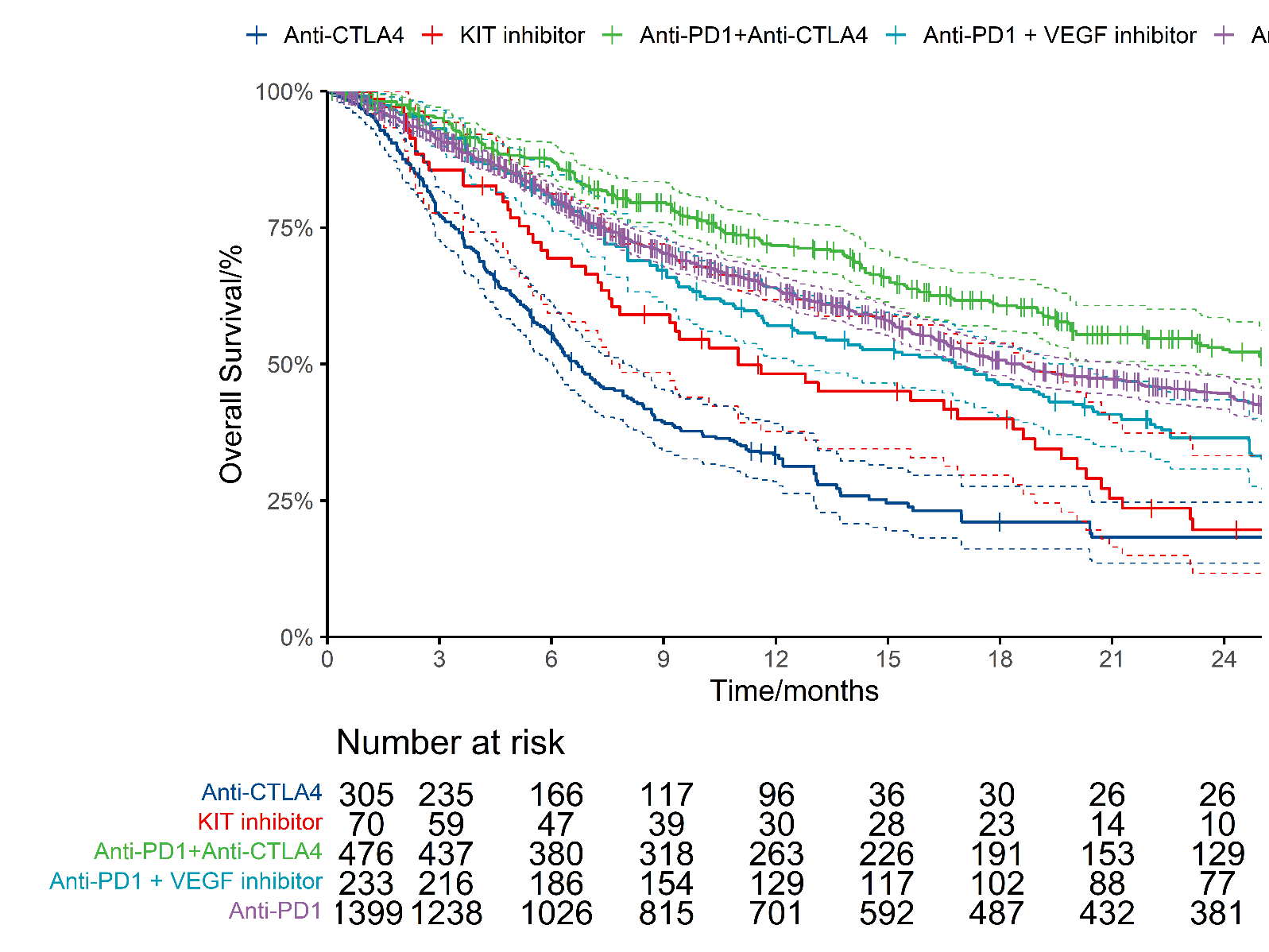
**

**Supplementary Figure 3: PFS of overall population stratified by treatment modalities**

**
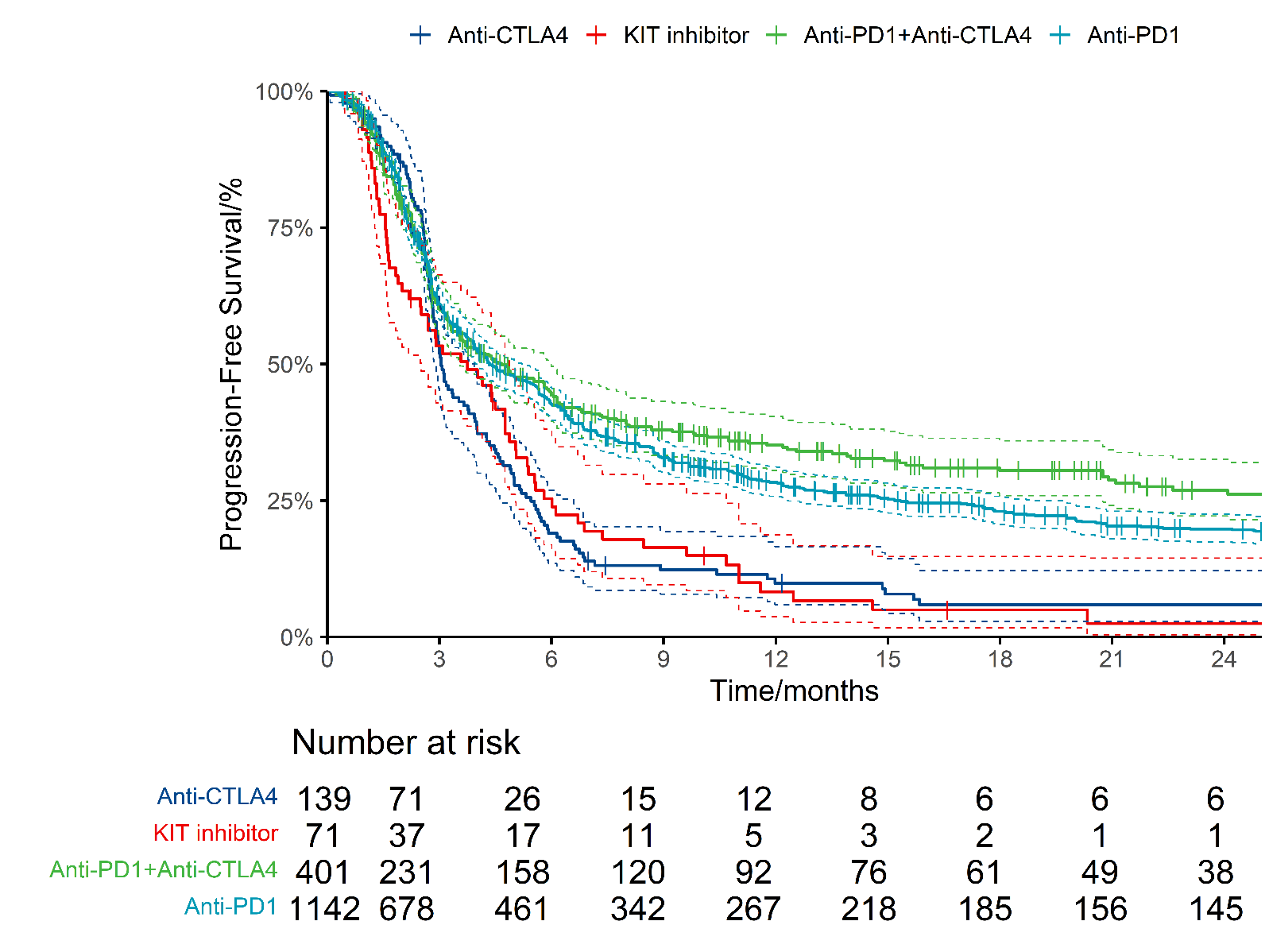
**

**Supplementary Figure 4: Two-stage meta-analysis of hazard ratios in double-arm studies comparing OS of patients treated with anti-PD1 and anti-CTLA4 combination therapy versus anti-PD1 monotherapy**


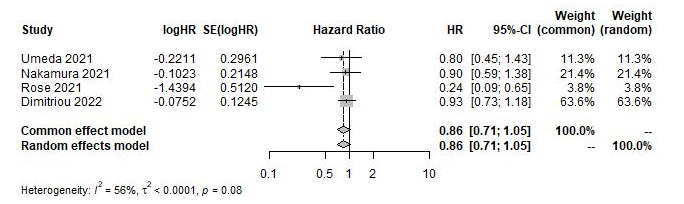


**Supplementary Figure 5: Two-stage meta-analysis of hazard ratios in double-arm studies comparing PFS of patients treated with anti-PD1 and anti-CTLA4 combination therapy versus anti-PD1 monotherapy**


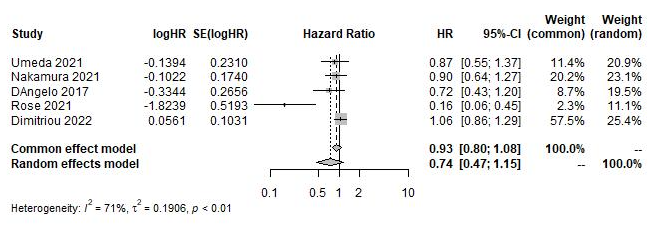


Supplementary Data 1: Search strategy

| **Mucosal melanoma**  Mucosal melanoma OR ((melanoma OR malignant melanoma) AND (mucosa OR mucous membrane OR mucosal))  AND  **Immunotherapy**  Immunotherapy OR antibodies OR PD-1 OR CTLA-4  **Targeted therapy**  Targeted therapy OR c-kit OR BRAF OR MEK |
| --- |

**Supplementary Data 2: Comparison of reconstructed graphs**

| Included in single or double arm analysis | Intervention | Outcome | Figure label in original study | Study label | Reconstructed graph |
| --- | --- | --- | --- | --- | --- |
| Single-arm | Anti-CTLA4 | PFS | 2B | Alexander et al. 2014 | 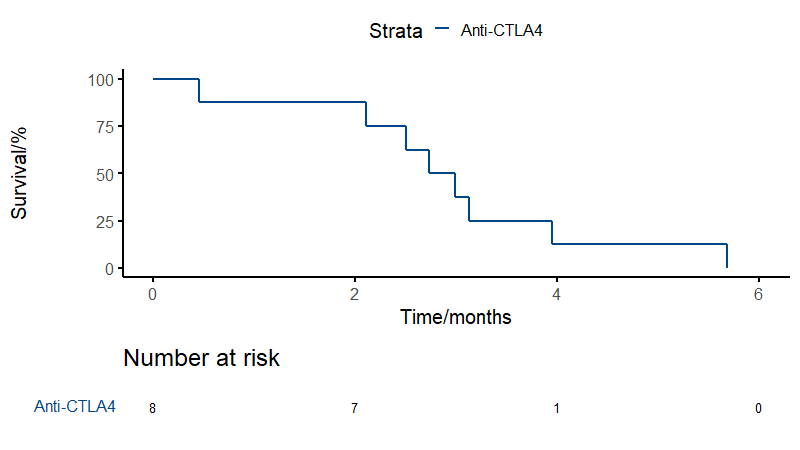 |
| Single-arm | Anti-CTLA4 | PFS | 1A | D’Angelo et al., 2017 | 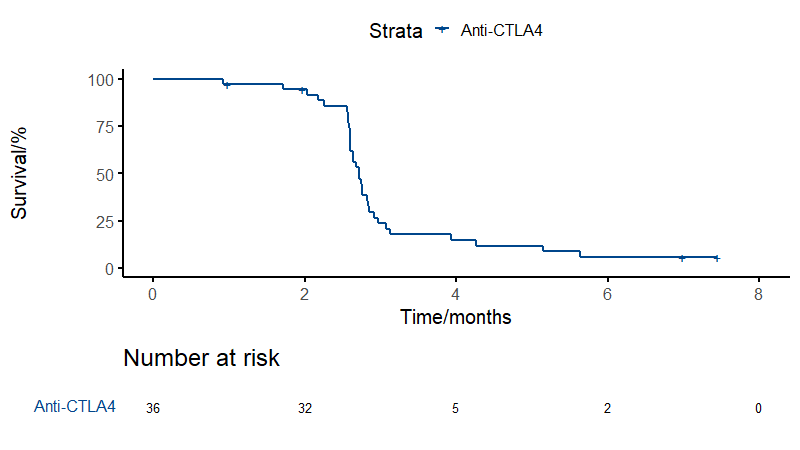 |
| Single-arm | Anti-CTLA4 | PFS | 2 | Del Vecchio et al., 2014 | 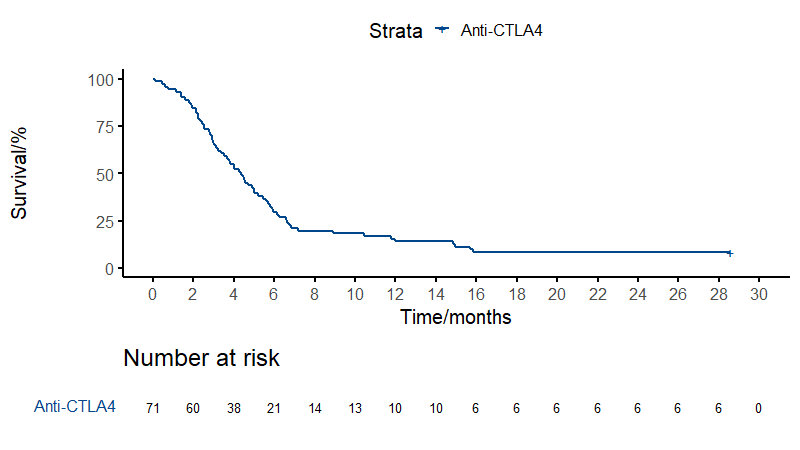 |
| Single-arm | Anti-CTLA4 | PFS | 2B | Moya-Plana et al., 2019 | 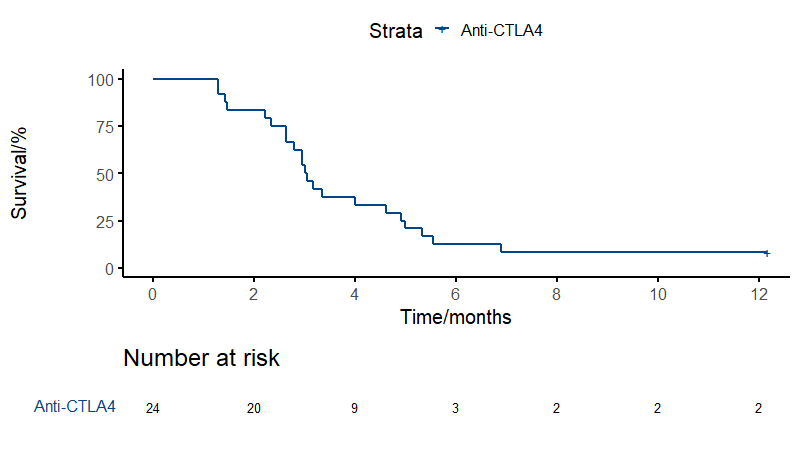 |
| Single-arm | Anti-CTLA4 | OS | 2A | Alexander et al., 2014 | 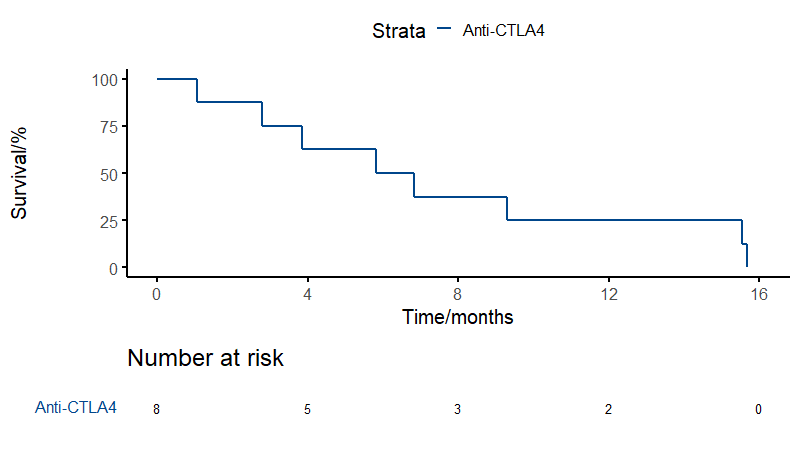 |
| Single-arm | Anti-CTLA4 | OS | 1 | Del Vecchio et al., 2014 | 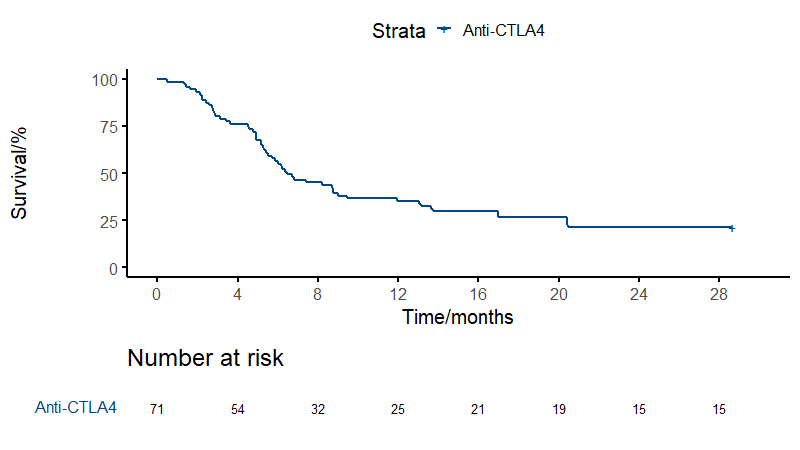 |
| Single-arm | Anti-CTLA4 | OS | 2A | Moya-Plana et al., 2019 | 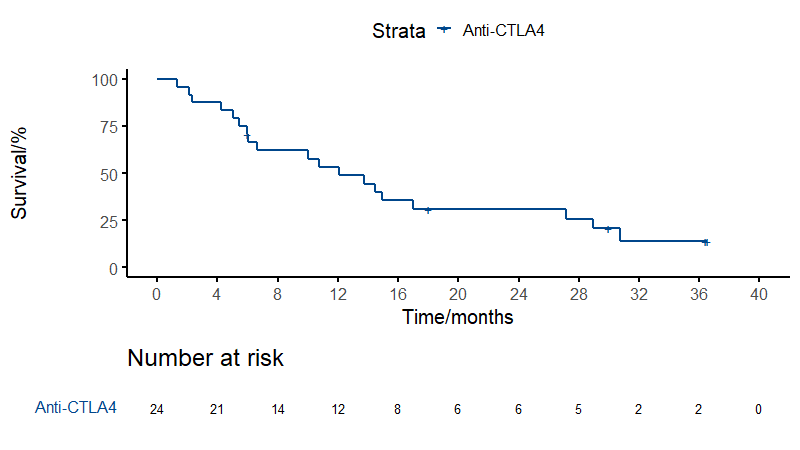 |
| Single-arm | Anti-CTLA4 | OS | 3 | Postow et al., 2013 | 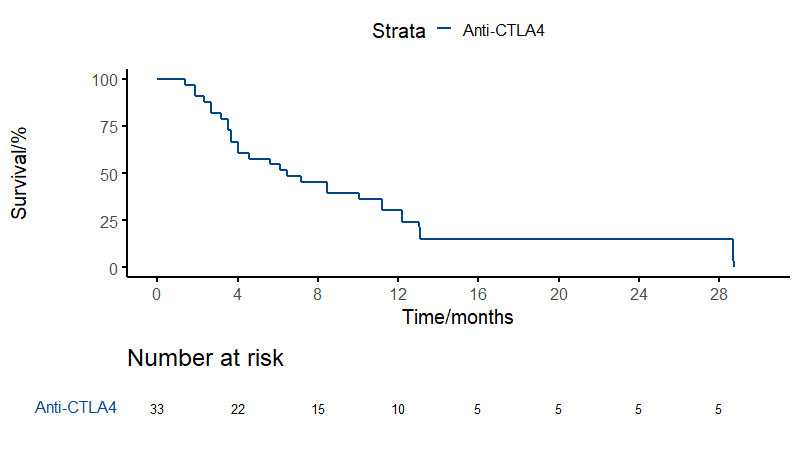 |
| Single-arm | Anti-CTLA4 | OS | 5D | Yamazaki et al., 2020 | 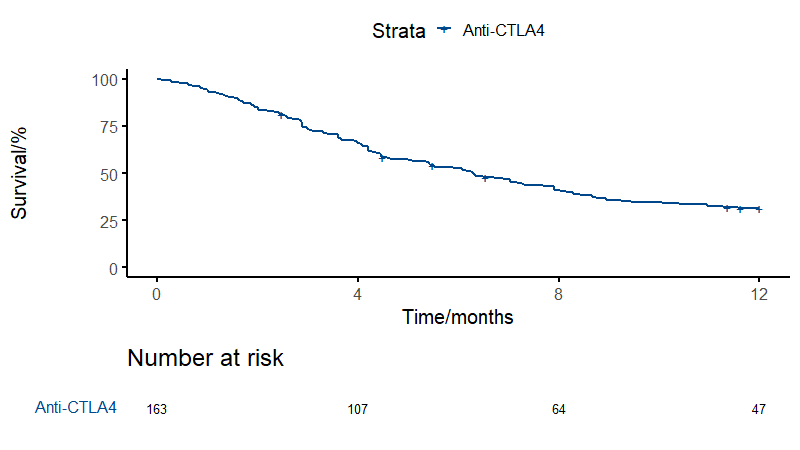 |
| Single-arm | Anti-CTLA4 | OS | 1B | Zimmer et al., 2015 | 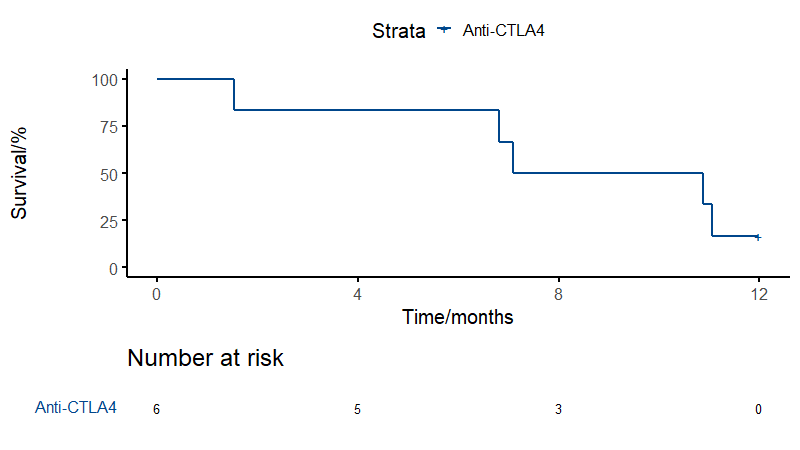 |
| Single-arm | Anti-PD1+Anti-CTLA4 | PFS | 1A | D’Angelo et al., 2017 | 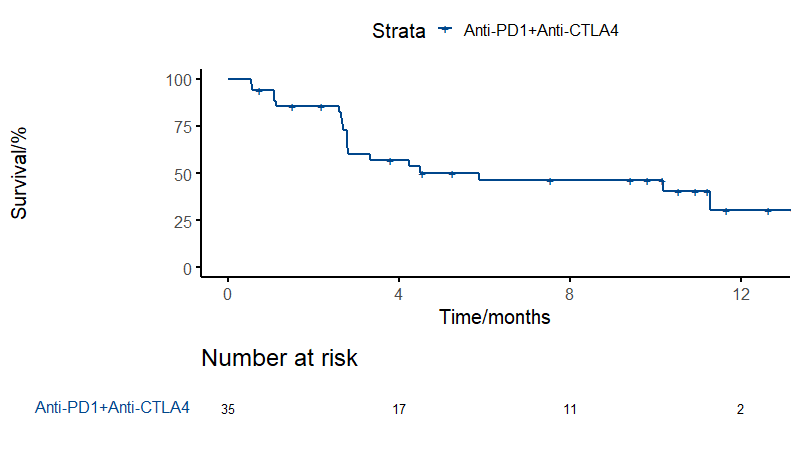 |
| Single-arm | Anti-PD1+Anti-CTLA4 | PFS | 3A | Dimitriou et al., 2022 | 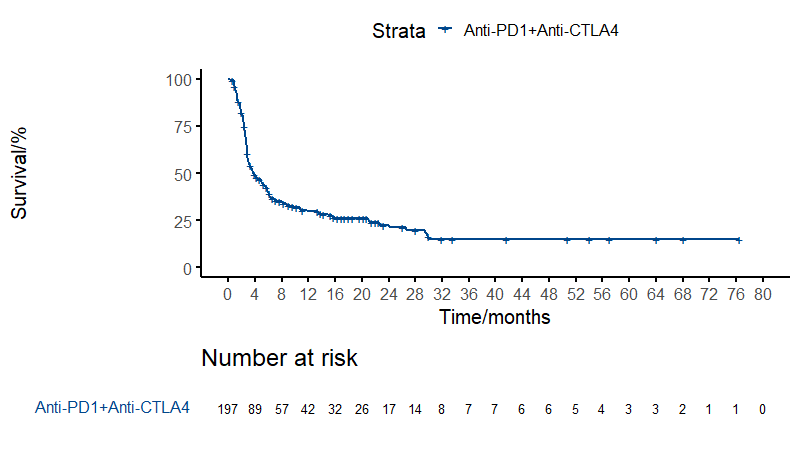 |
| Single-arm | Anti-PD1+Anti-CTLA4 | PFS | Supplementary Figure 1B | Ho et al., 2022 | 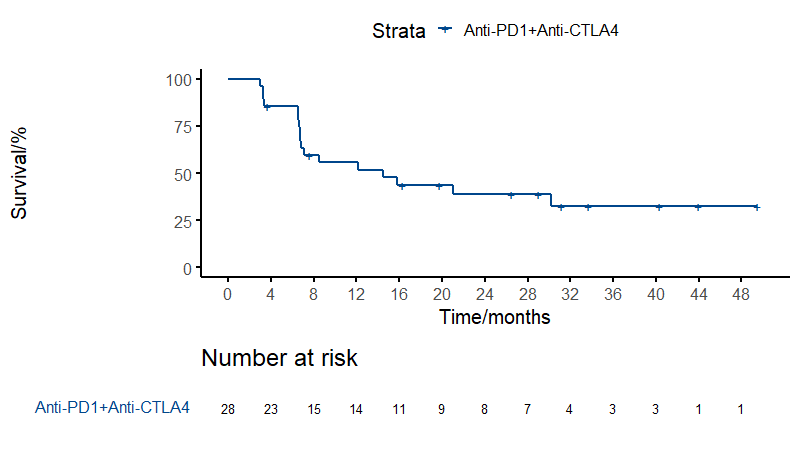 |
| Single-arm | Anti-PD1+Anti-CTLA4 | PFS | 1A | Nakamura et al., 2021 | 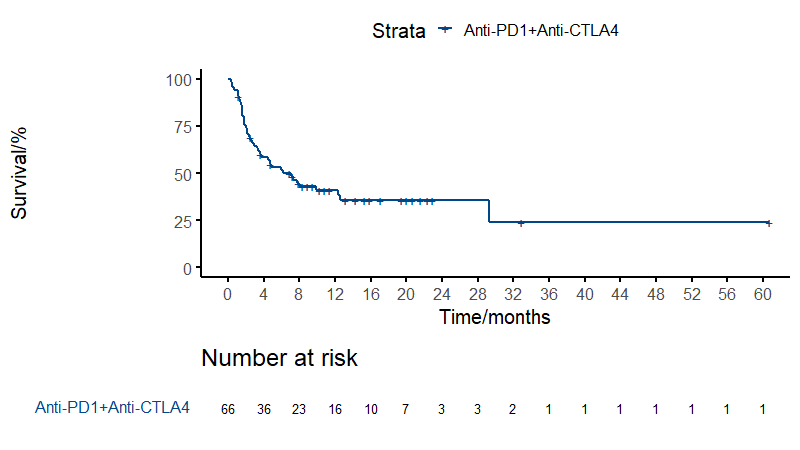 |
| Single-arm | Anti-PD1+Anti-CTLA4 | PFS | 5 | Namikawa et al., 2018 | 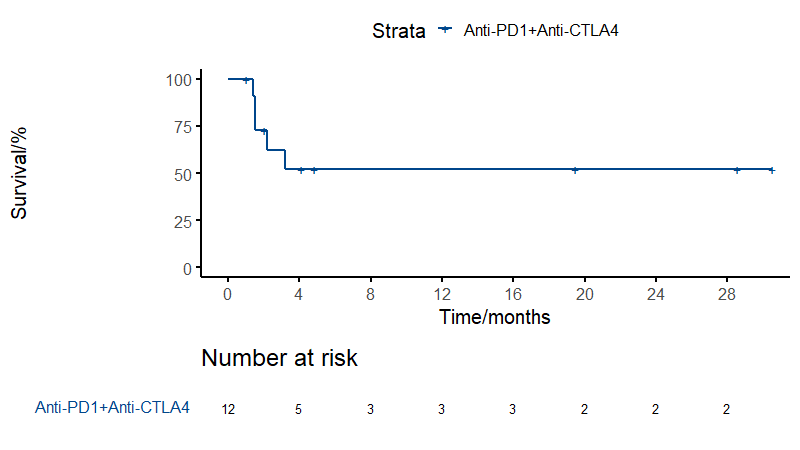 |
| Single-arm | Anti-PD1+Anti-CTLA4 | PFS | 4C | Rose et al., 2021 | 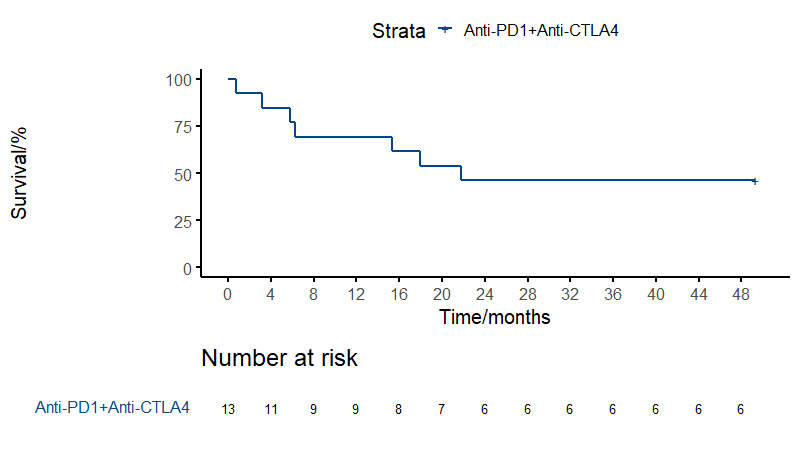 |
| Single-arm | Anti-PD1+Anti-CTLA4 | PFS | 4A | Takahashi et al., 2020 | 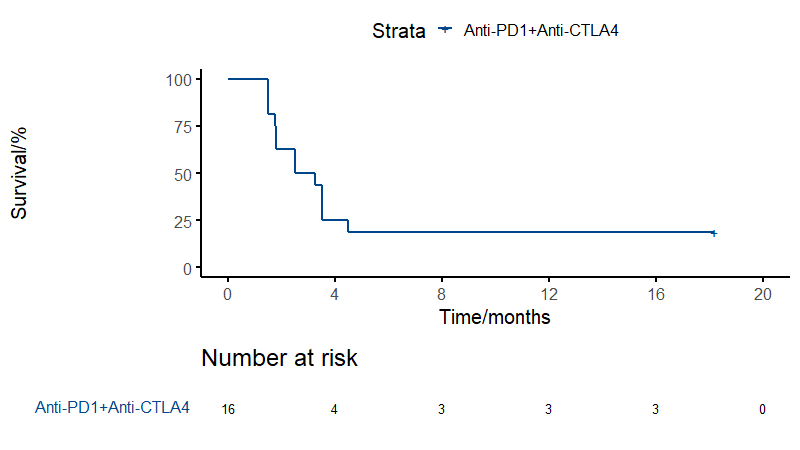 |
| Single-arm | Anti-PD1+Anti-CTLA4 | PFS | 3A | Umeda et al., 2021 | 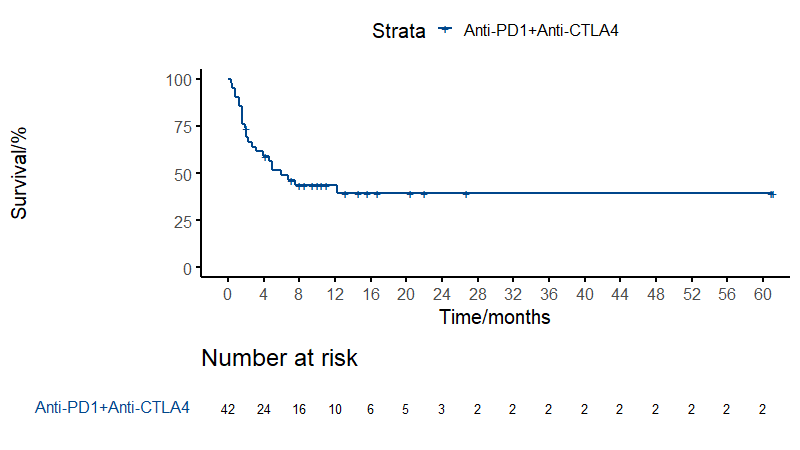 |
| Single-arm | Anti-PD1+Anti-CTLA4 | PFS | 3A | Takahashi et al., 2023 | 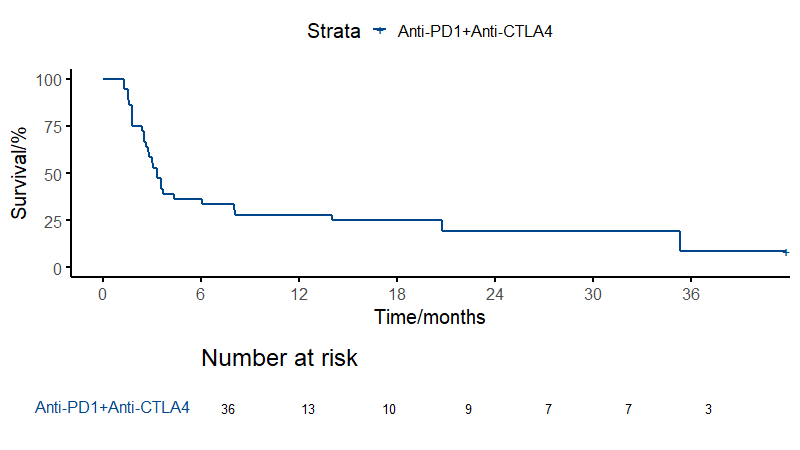 |
| Single-arm | Anti-PD1+Anti-CTLA4 | OS | 3B | Dimitriou et al., 2022 | 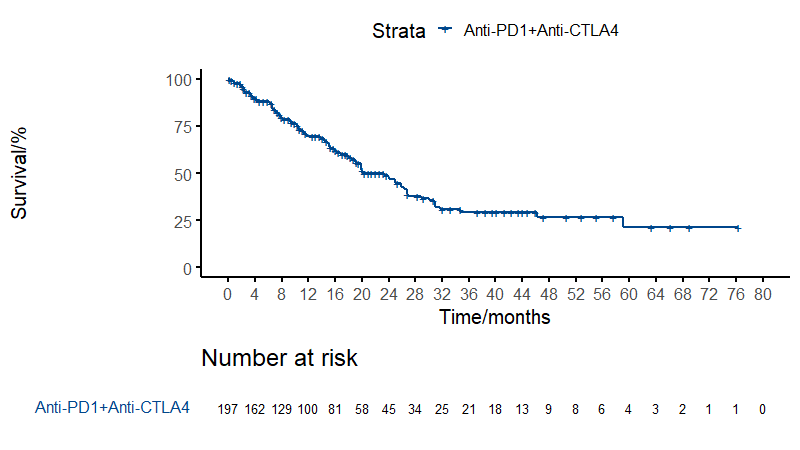 |
| Single-arm | Anti-PD1+Anti-CTLA4 | OS | Supplementary Figure 1A | Ho et al., 2022 | 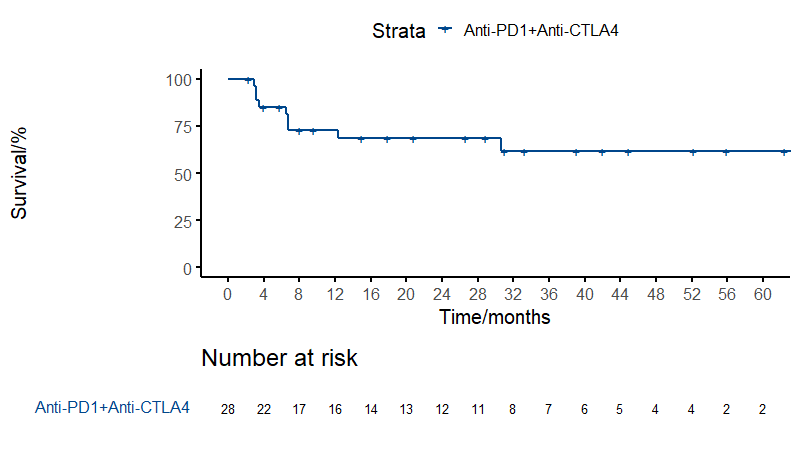 |
| Single-arm | Anti-PD1+Anti-CTLA4 | OS | 3H | Hodi et al., 2021 | 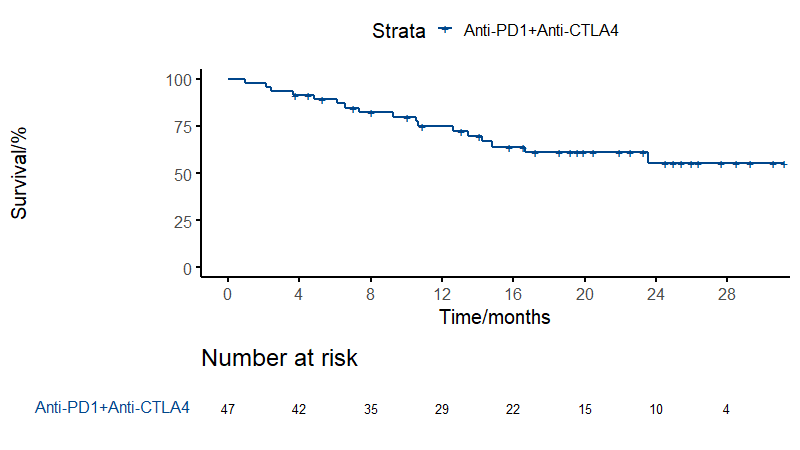 |
| Single-arm | Anti-PD1+Anti-CTLA4 | OS | 1B | Nakamura et al., 2021 | 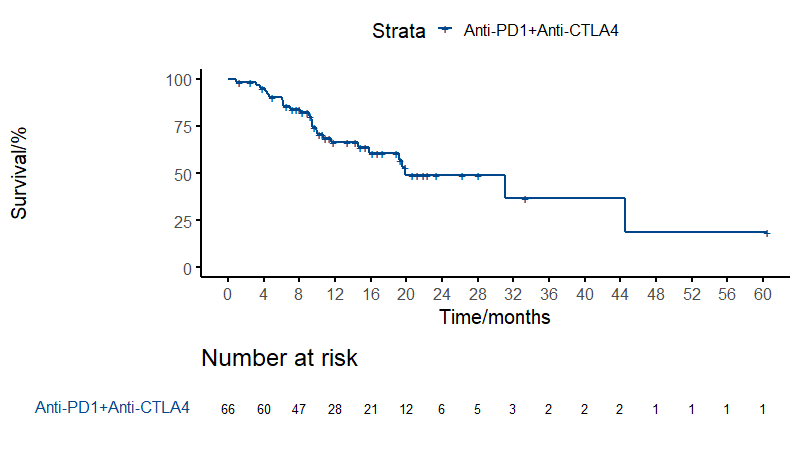 |
| Single-arm | Anti-PD1+Anti-CTLA4 | OS | 4 | Namikawa et al., 2018 | 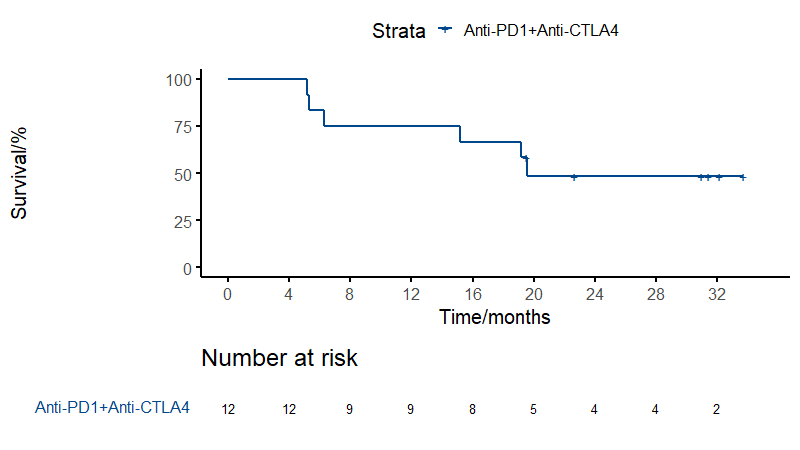 |
| Single-arm | Anti-PD1+Anti-CTLA4 | OS | 4D | Rose et al., 2021 | 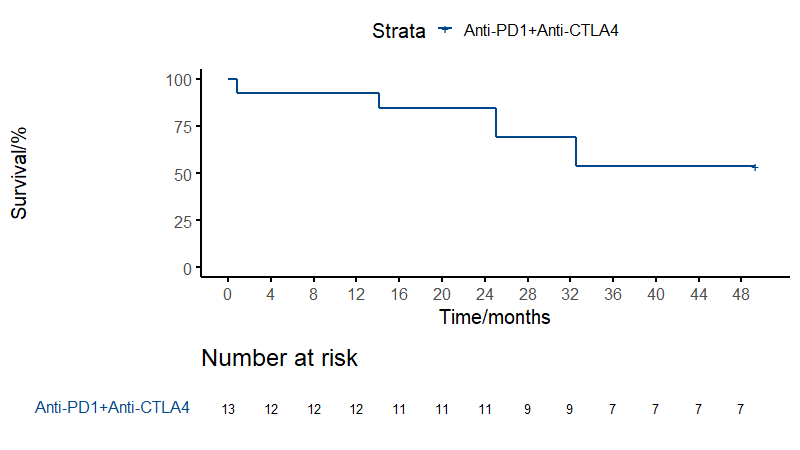 |
| Single-arm | Anti-PD1+Anti-CTLA4 | OS | 2 | Kottschade et al, 2023 | 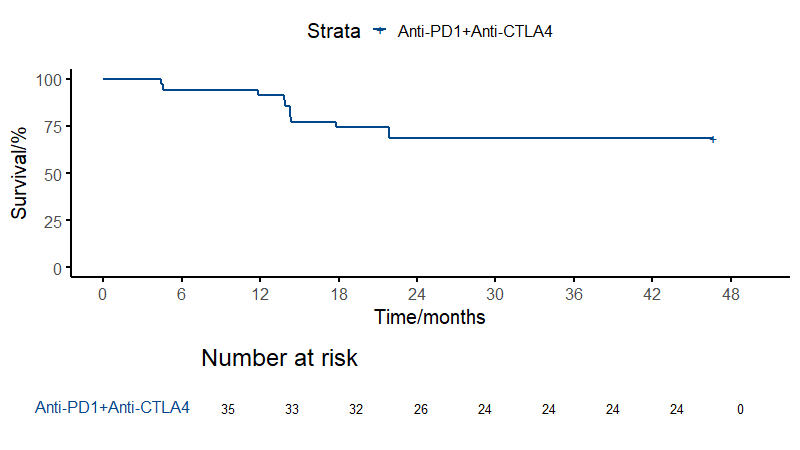 |
| Single-arm | Anti-PD1+Anti-CTLA4 | OS | 3B | Takahashi et al., 2023 | 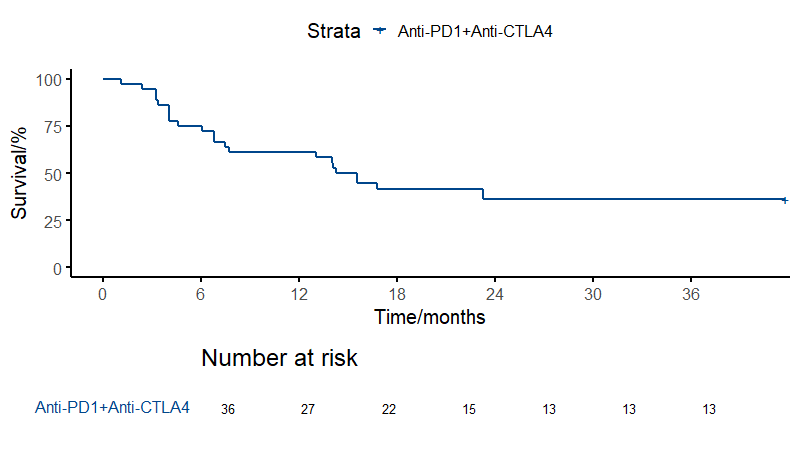 |
| Single-arm | Anti-PD1+Anti-CTLA4 | OS | 3B | Umeda et al., 2021 | 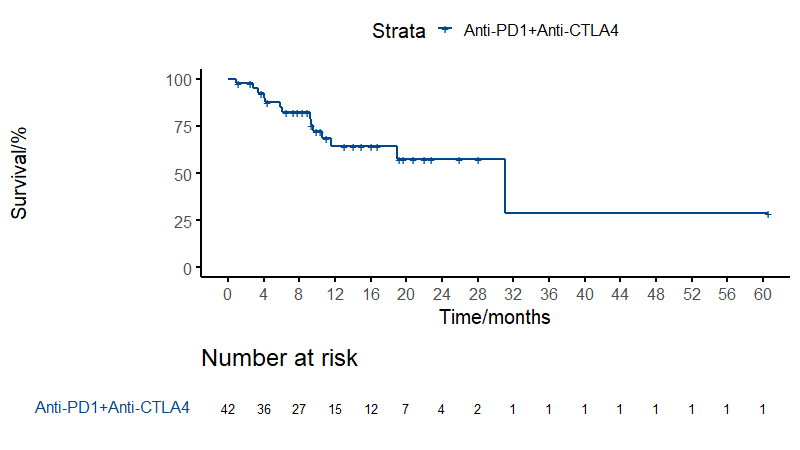 |
| Single-arm | Anti-PD1 | PFS | 1A | D’Angelo et al., 2017 | 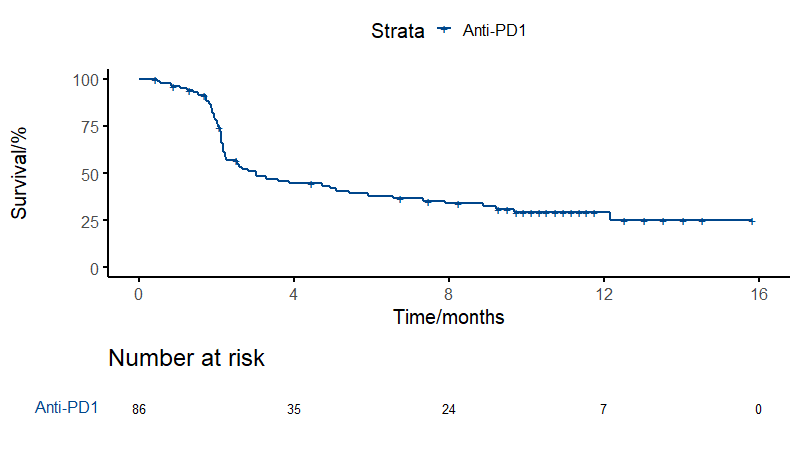 |
| Single-arm | Anti-PD1 | PFS | 3A | Dimitriou et al., 2022 | 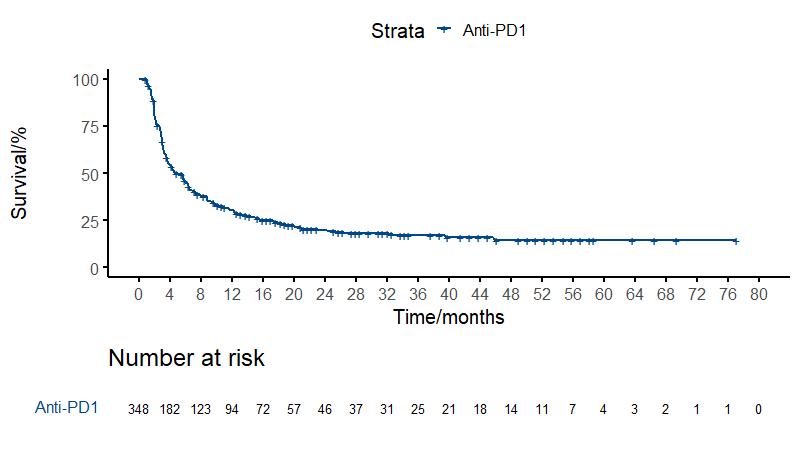 |
| Single-arm | Anti-PD1 | PFS | 2A | Hamid et al., 2018 | 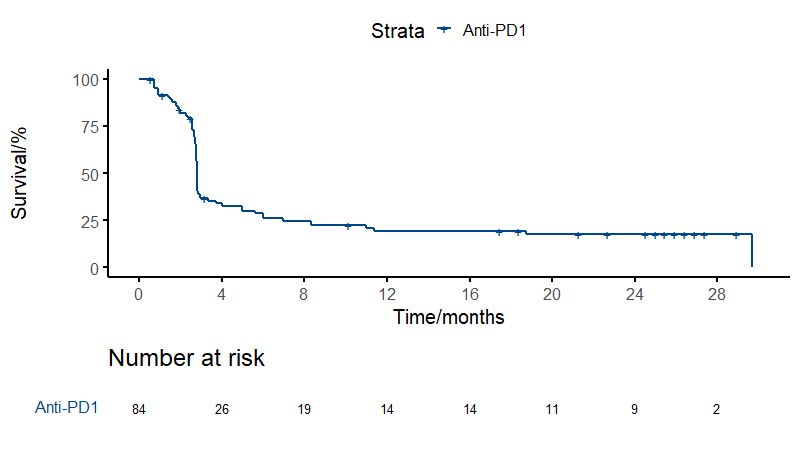 |
| Single-arm | Anti-PD1 | PFS | 3B | Si et al., 2022 | 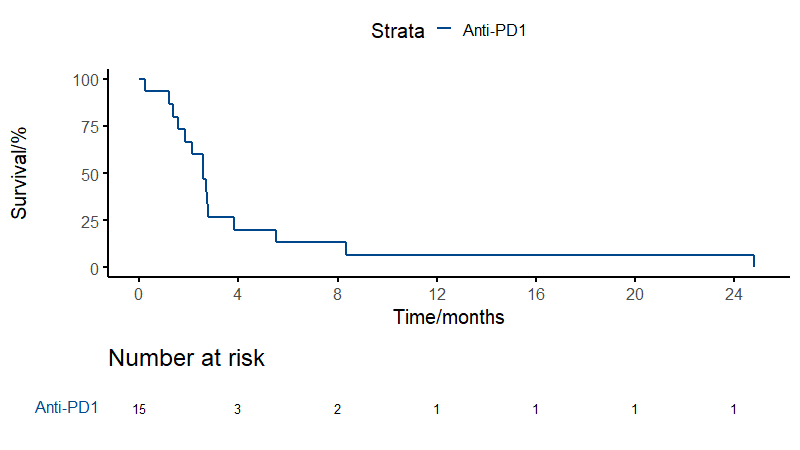 |
| Single-arm | Anti-PD1 | PFS | 2B | Moya-Plana et al., 2019 | 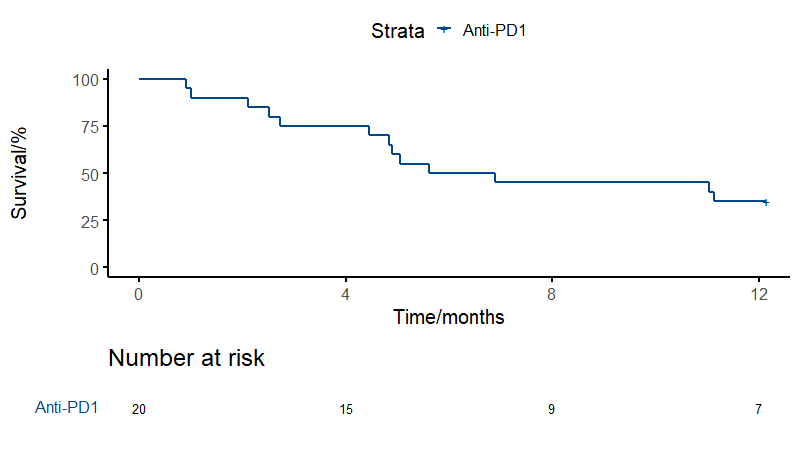 |
| Single-arm | Anti-PD1 | PFS | 1A | Nakamura et al., 2021 | 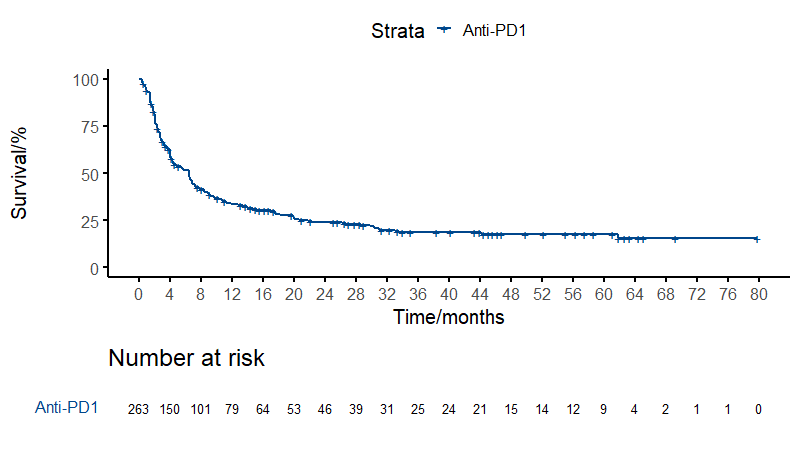 |
| Single-arm | Anti-PD1 | PFS | 3 | Nomura et al., 2020 | 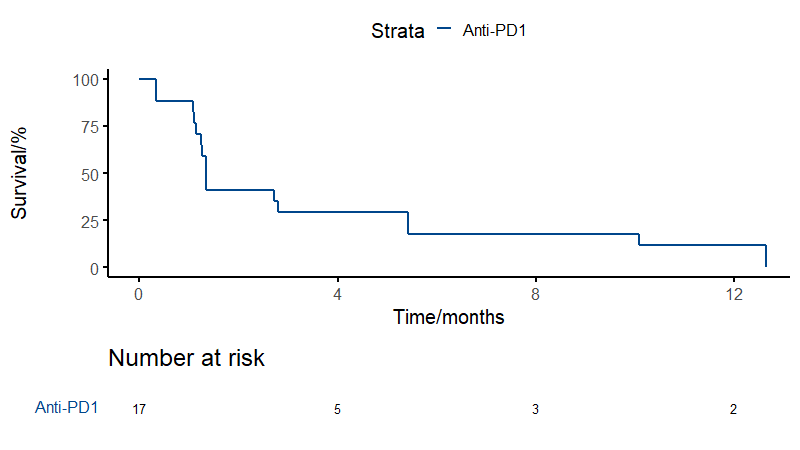 |
| Single-arm | Anti-PD1 | PFS | 1A | Ogata et al., 2021 | 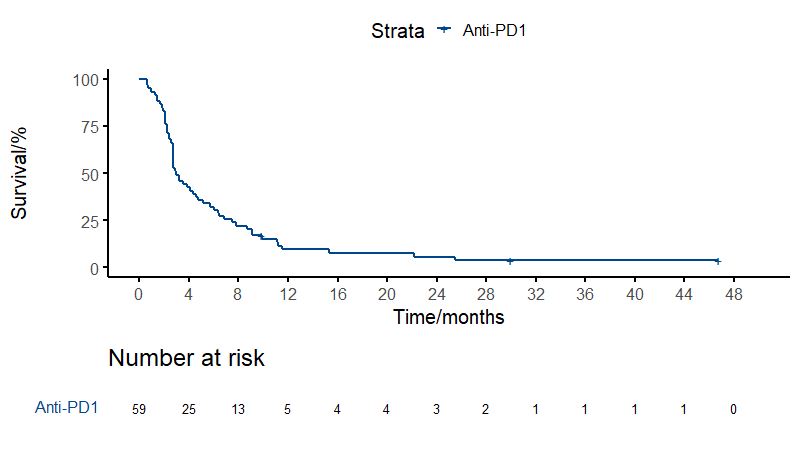 |
| Single-arm | Anti-PD1 | PFS | 4A | Kim et al., 2019 | 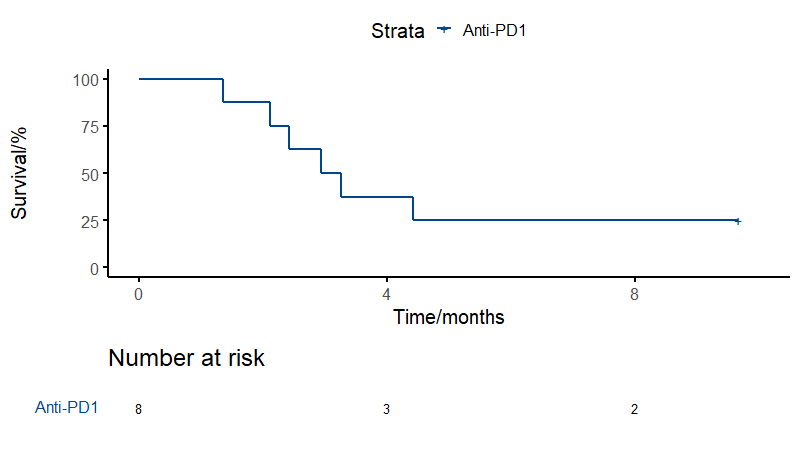 |
| Single-arm | Anti-PD1 | PFS | 4C | Rose et al., 2021 | 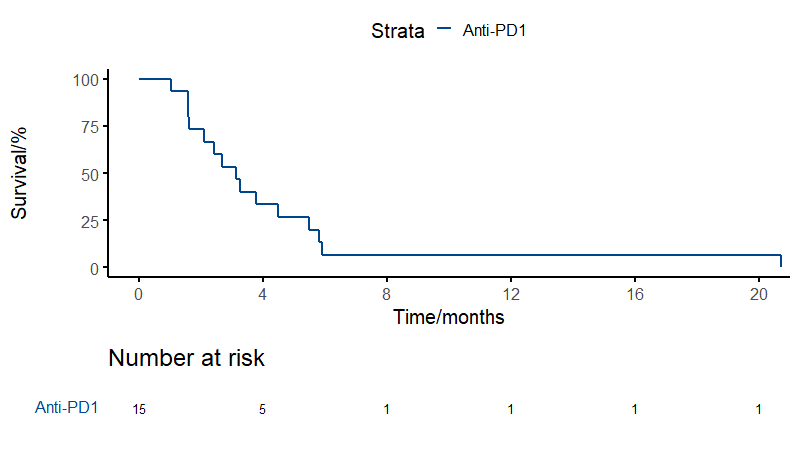 |
| Single-arm | Anti-PD1 | PFS | 2B | Shoushtari et al., 2016 | 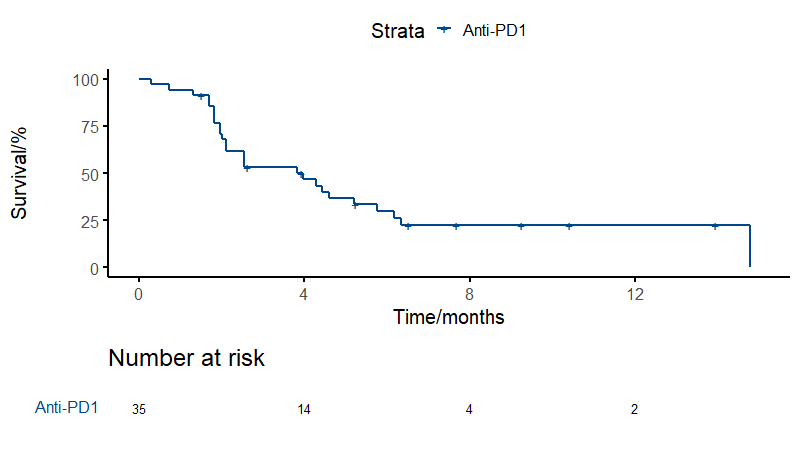 |
| Single-arm | Anti-PD1 | PFS | 2A | Teterycz et al., 2020 | 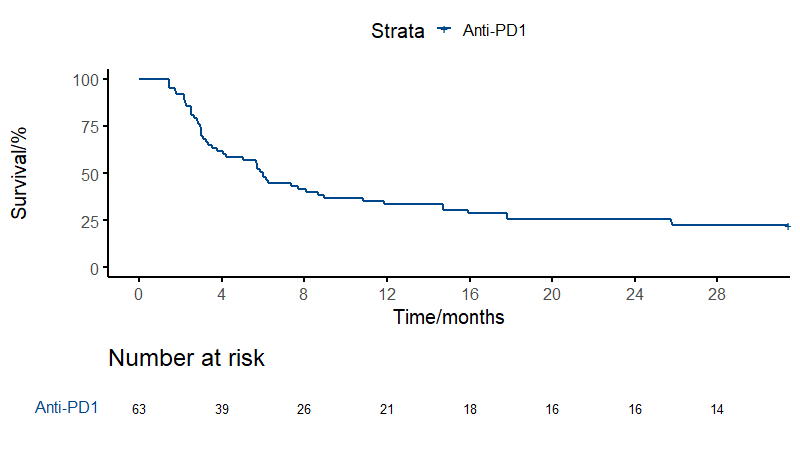 |
| Single-arm | Anti-PD1 | PFS | 3B | Uhara et al., 2021 | 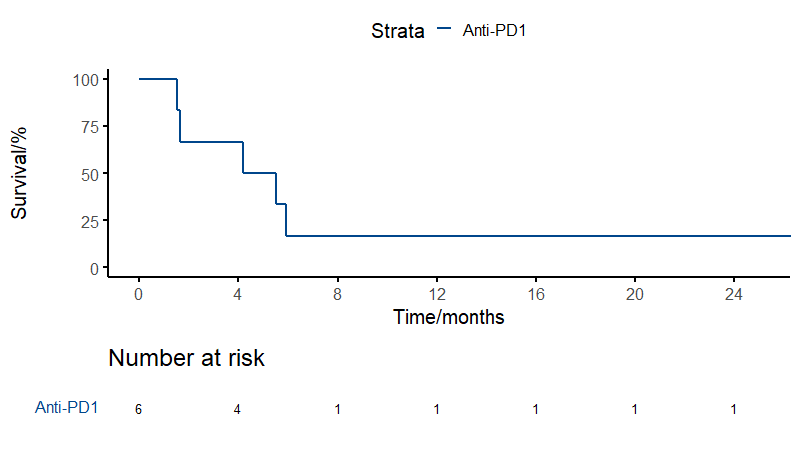 |
| Single-arm | Anti-PD1 | PFS | 2A | Umeda et al., 2021 | 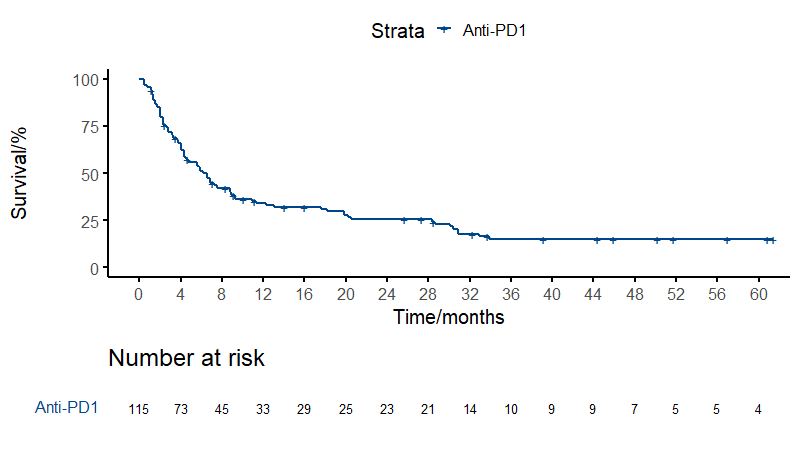 |
| Single-arm | Anti-PD1 | PFS | 2A | Yamazaki et al., 2017 | 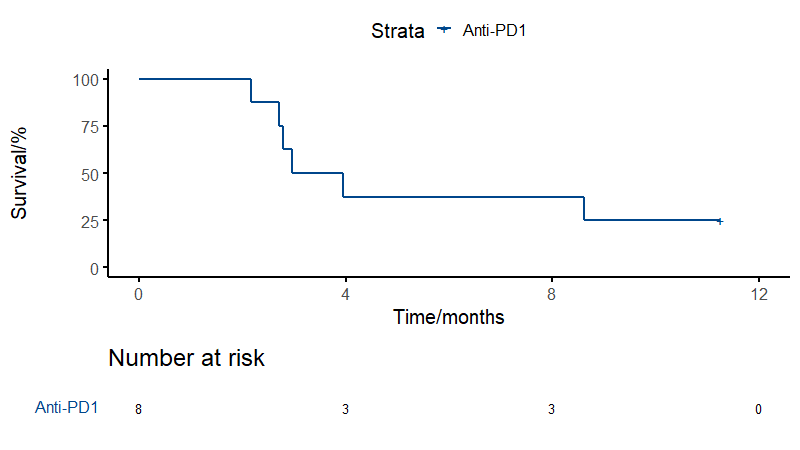 |
| Single-arm | Anti-PD1 | OS | 3B | Dimitriou et al., 2022 | 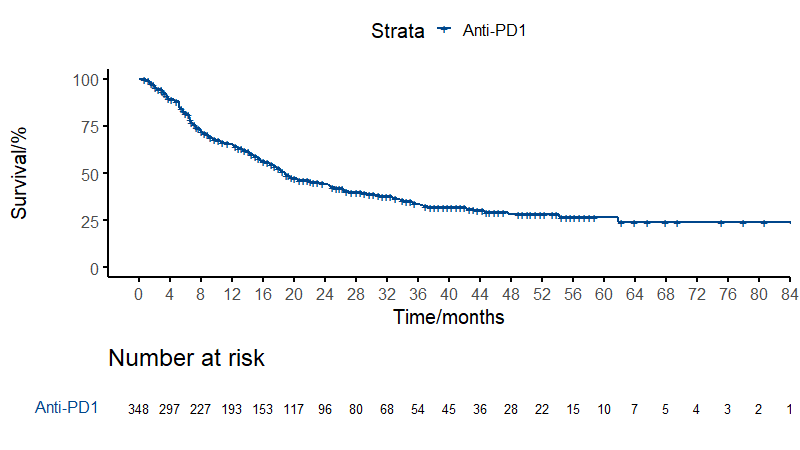 |
| Single-arm | Anti-PD1 | OS | 2B | Hamid et al., 2018 | 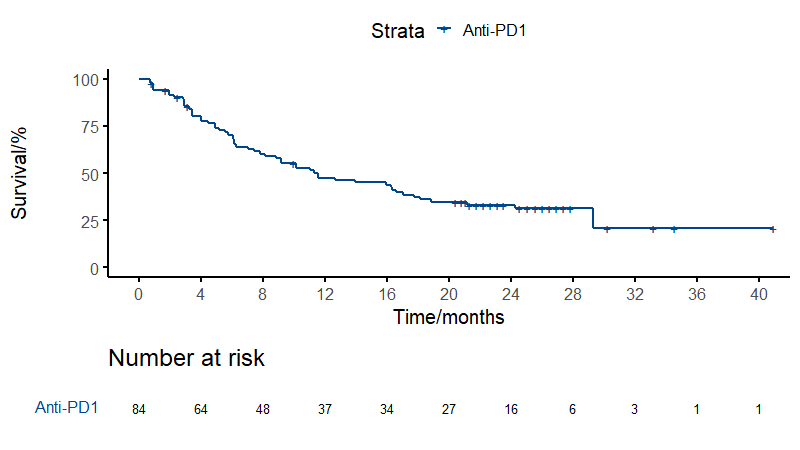 |
| Single-arm | Anti-PD1 | OS | 5 | Kiyohara et al., 2018 | 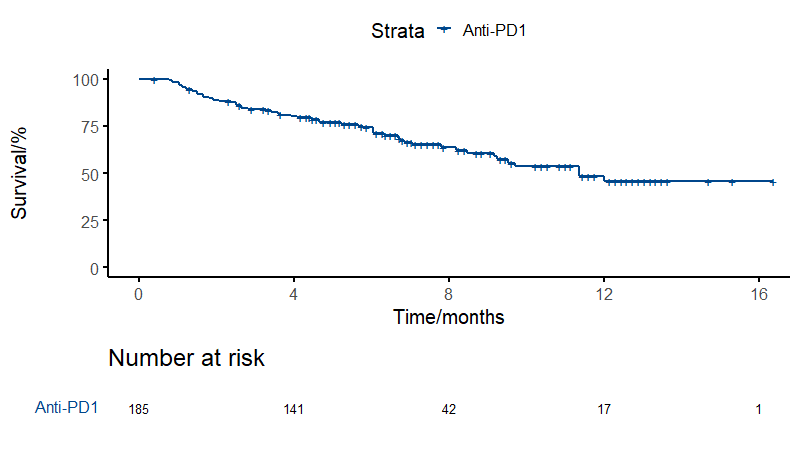 |
| Single-arm | Anti-PD1 | OS | 4B | Si et al., 2022 | 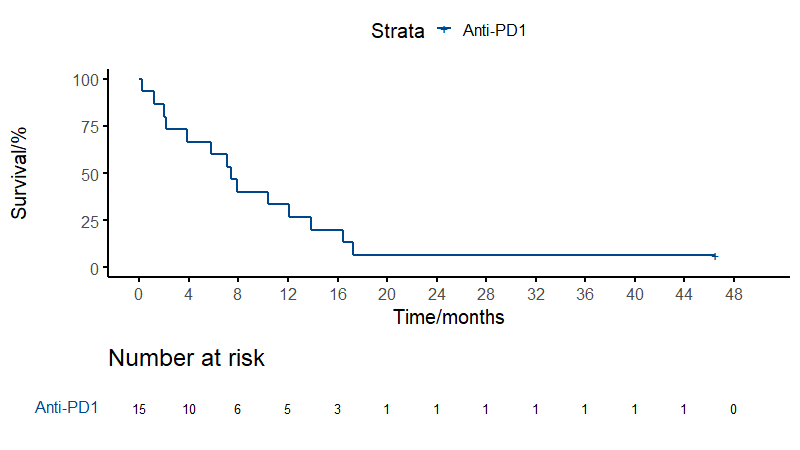 |
| Single-arm | Anti-PD1 | OS | 2A | Moya-Plana et al., 2019 | 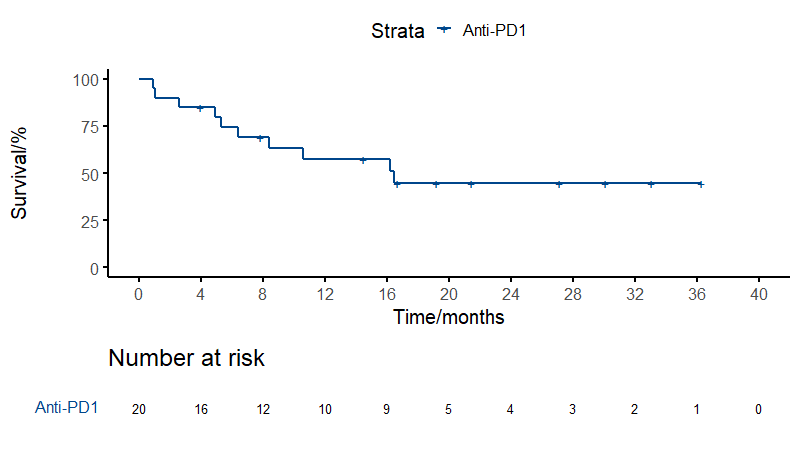 |
| Single-arm | Anti-PD1 | OS | 1B | Nakamura et al., 2021 | 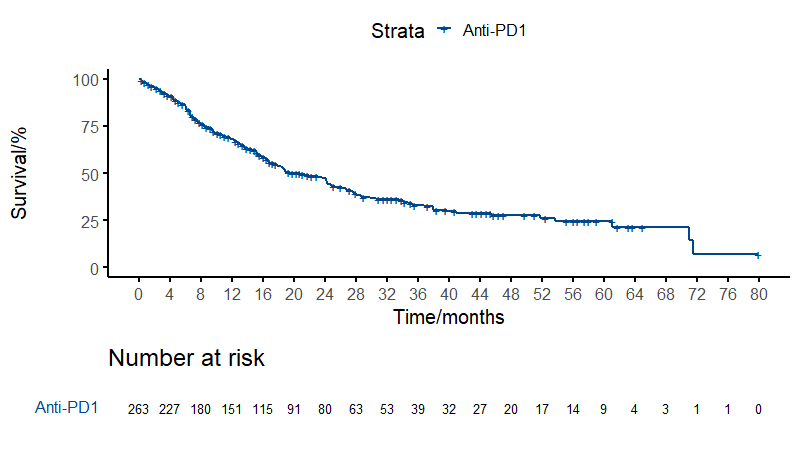 |
| Single-arm | Anti-PD1 | OS | 1 | Nathan et al., 2019 | 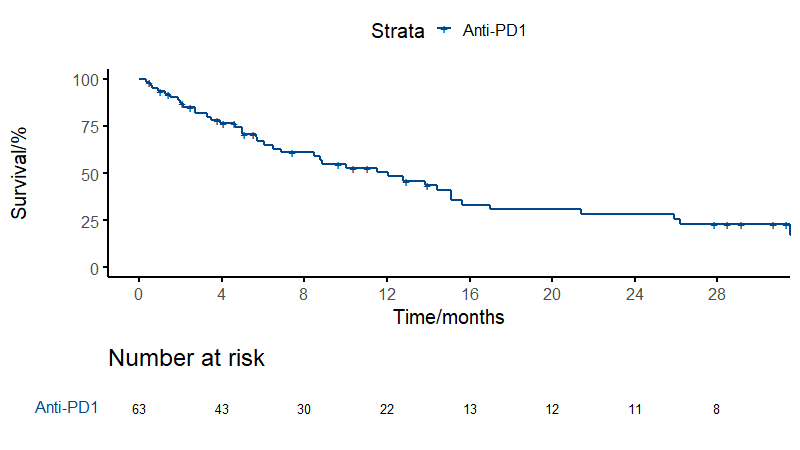 |
| Single-arm | Anti-PD1 | OS | 3 | Nomura et al., 2020 | 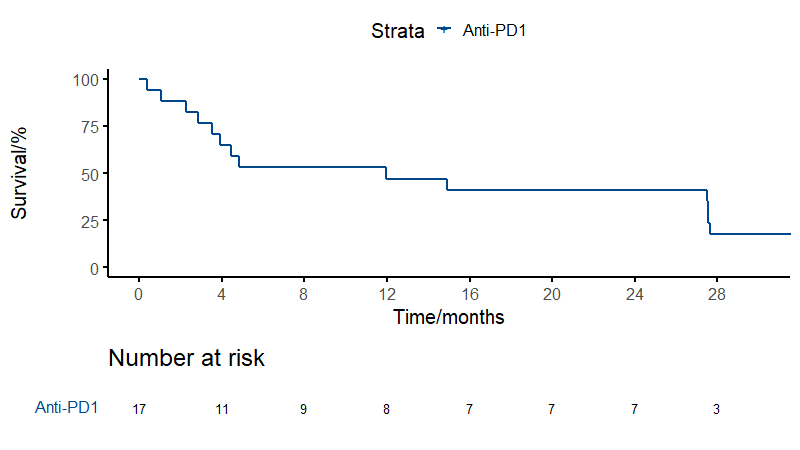 |
| Single-arm | Anti-PD1 | OS | 1B | Ogata et al., 2021 | 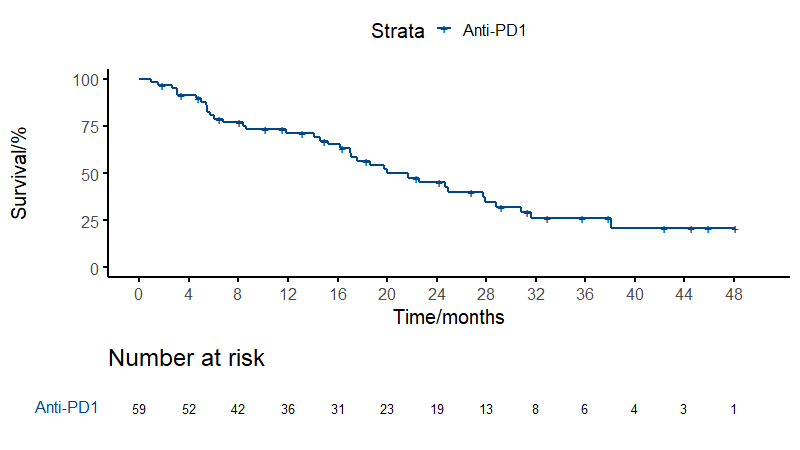 |
| Single-arm | Anti-PD1 | OS | 4B | Kim et al., 2019 | 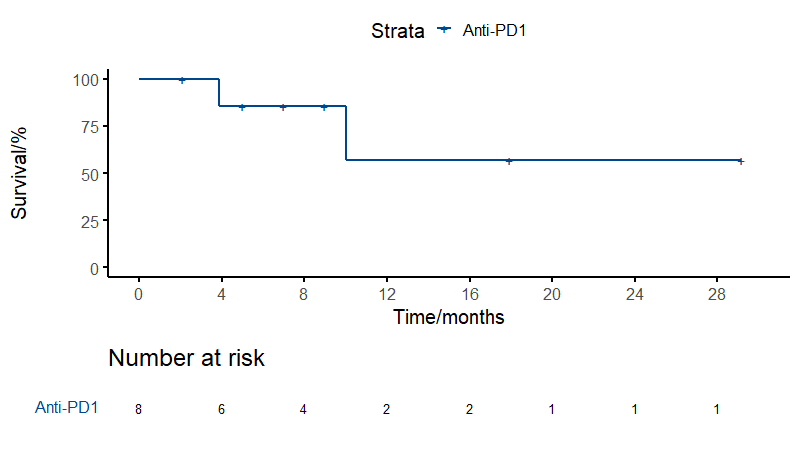 |
| Single-arm | Anti-PD1 | OS | 4D | Rose et al., 2021 | 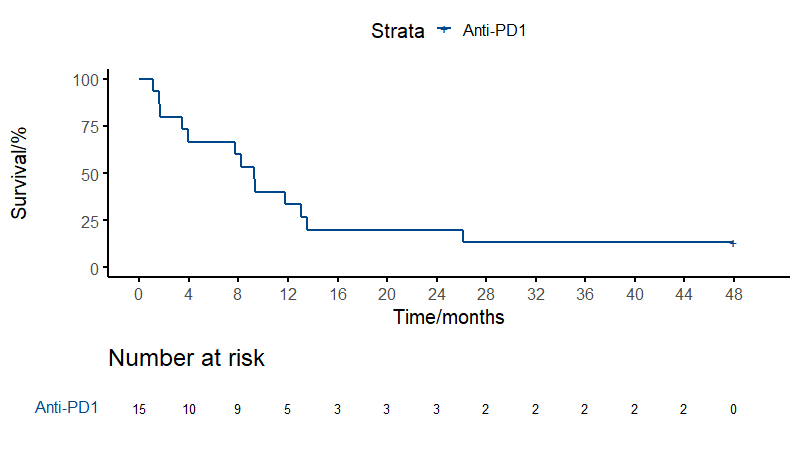 |
| Single-arm | Anti-PD1 | OS | 2B | Teterycz et al., 2020 | 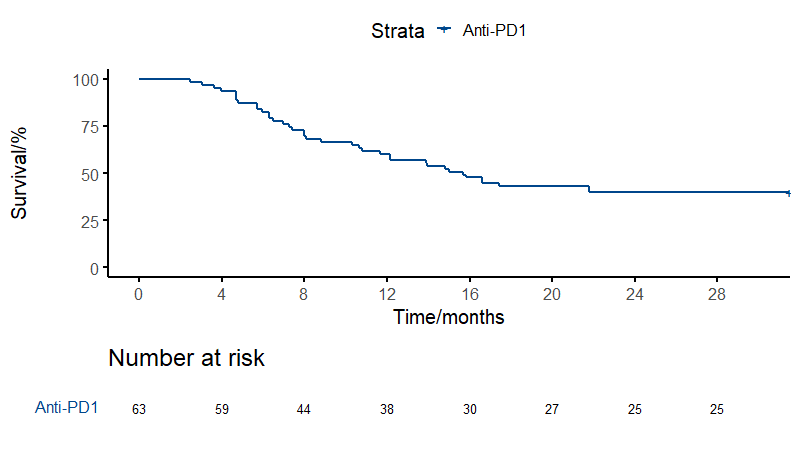 |
| Single-arm | Anti-PD1 | OS | 3A | Uhara et al., 2021 | 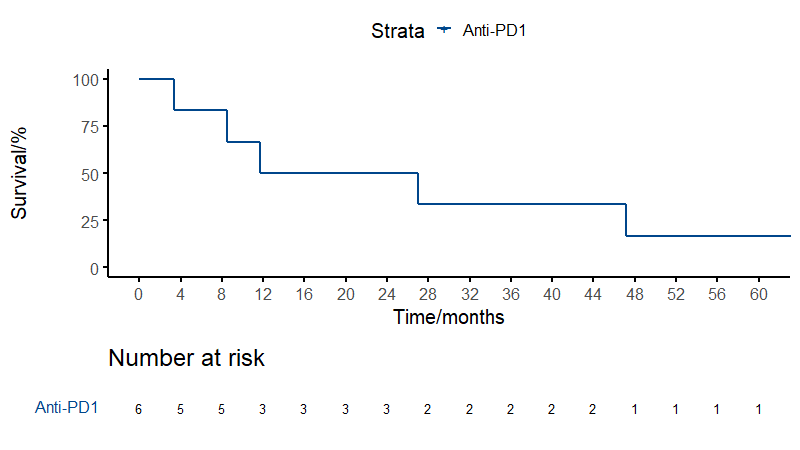 |
| Single-arm | Anti-PD1 | OS | 2B | Umeda et al., 2021 | 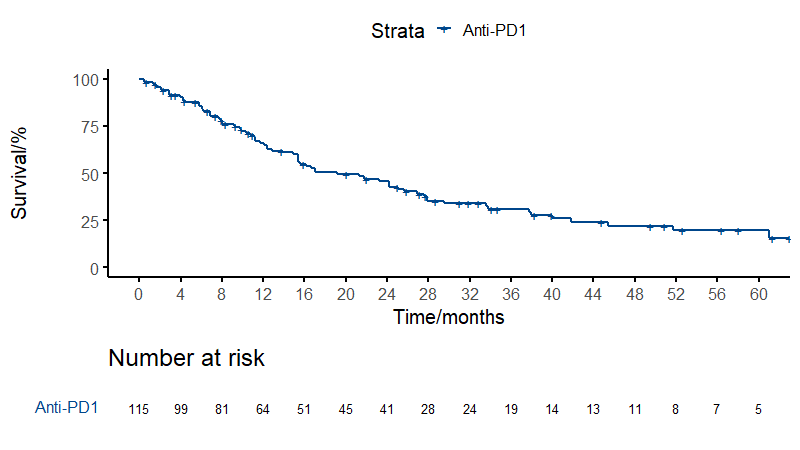 |
| Single-arm | Anti-PD1 | OS | 2B | Yamazaki et al., 2017 | 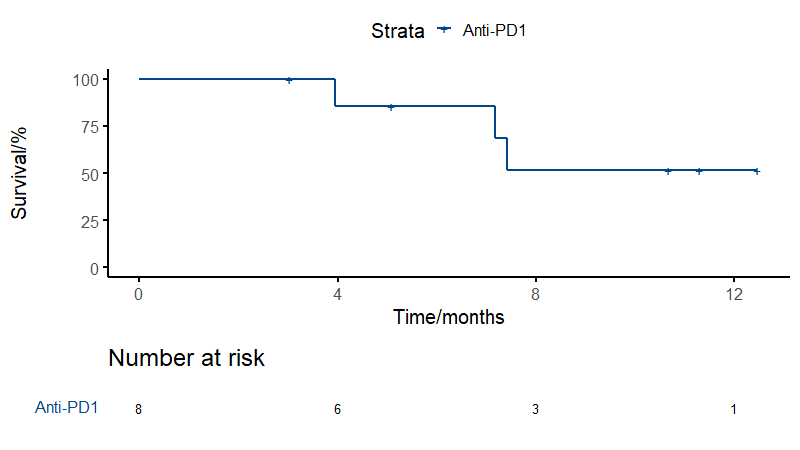 |
| Single-arm | Anti-PD1 | OS | 2C | Jacques et al ., 2024 | 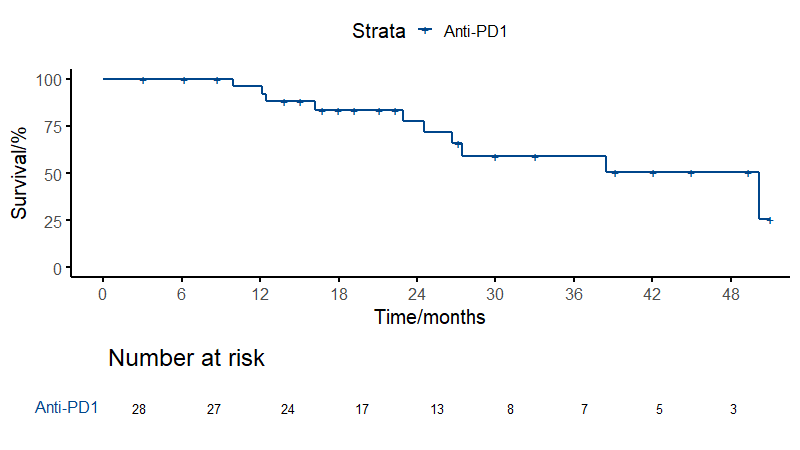 |
| Single-arm | Anti-PD1 | OS | 2D | Xue et al., 2024 | 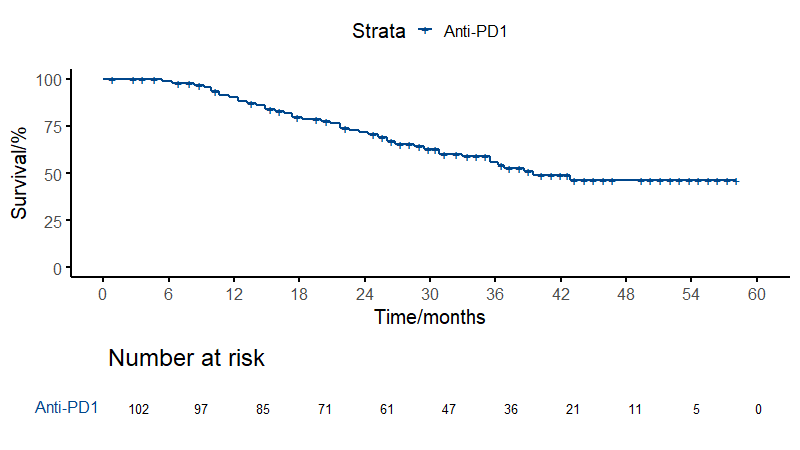 |
| Single-arm | Anti-PD1+VEGF inhibitors | OS | 2C | Li et al., 2022 | 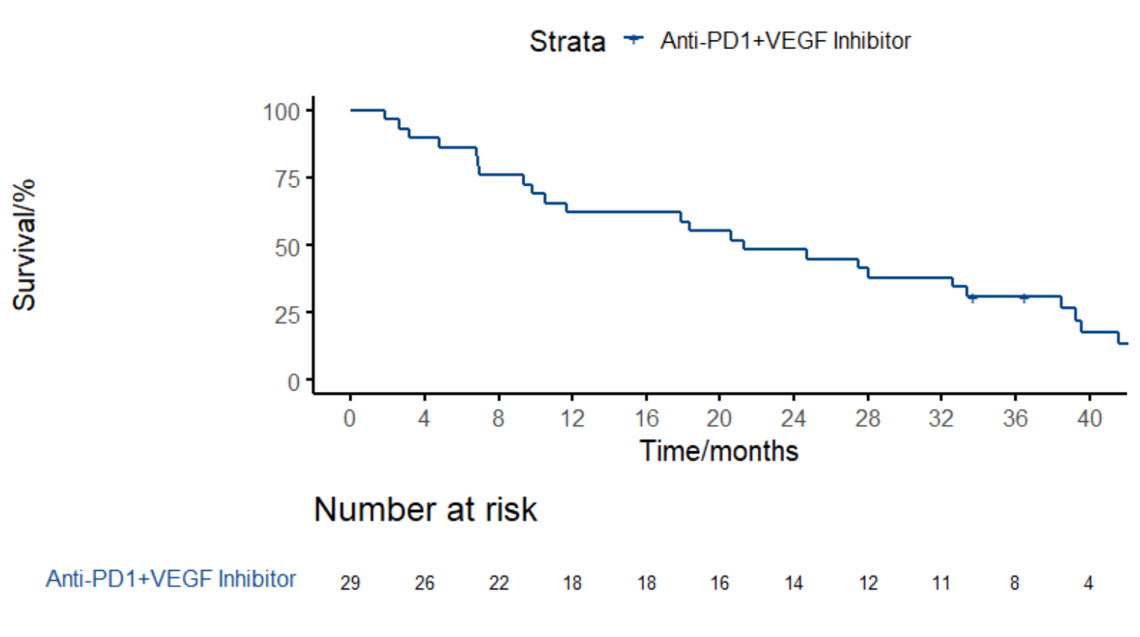 |
| Single-arm | Anti-PD1+ VEGF inhibitors | OS | 1C | Tang et al., 2021 | 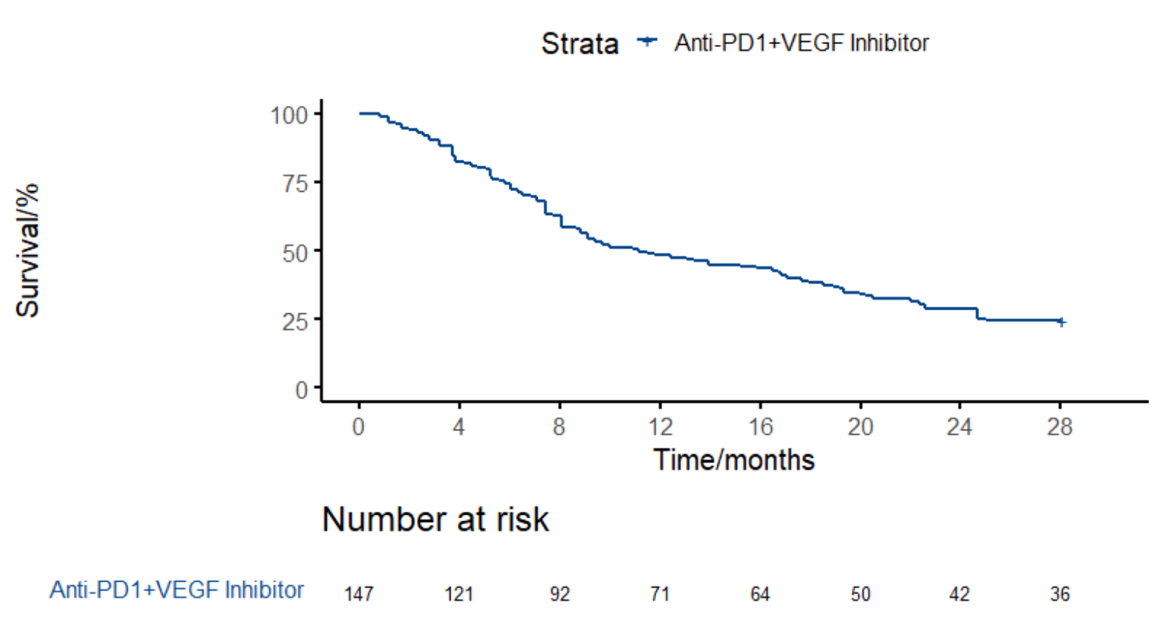 |
| Single-arm | Anti-PD1+ VEGF inhibitors | OS | 2B | Lian et al., 2024 | 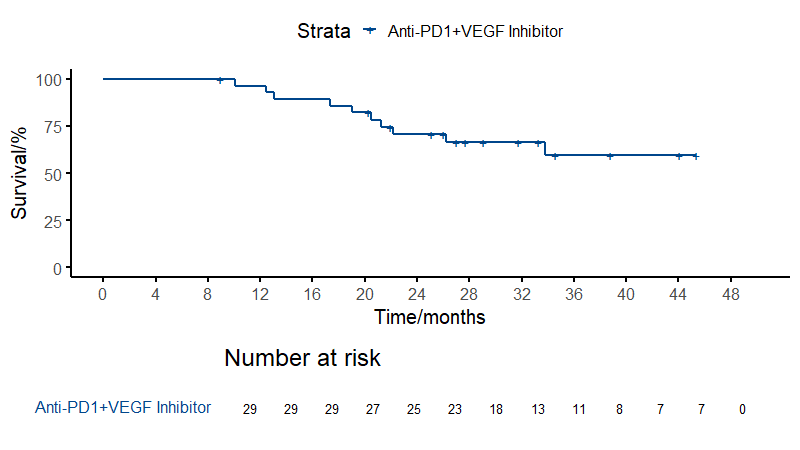 |
| Single-arm | Anti-PD1+ VEGF inhibitors | OS | 3B | Zhao et al., 2024 | 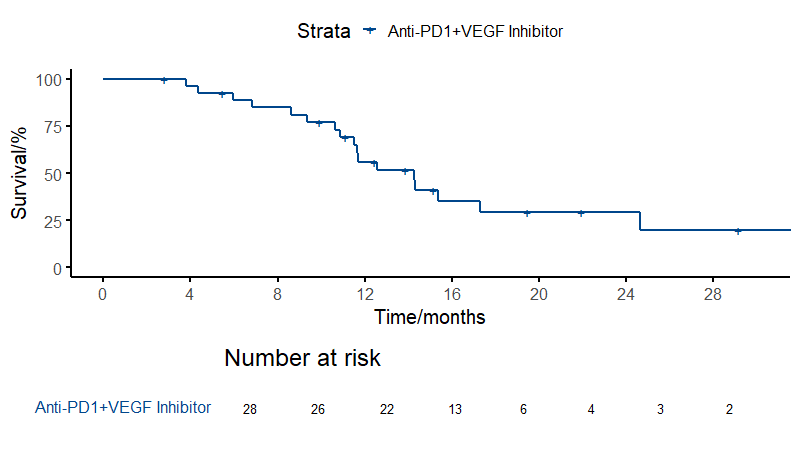 |
| Double-arm | Anti-PD1 vs Anti-PD1+RT | OS | 4B | Kim et al., 2019 | 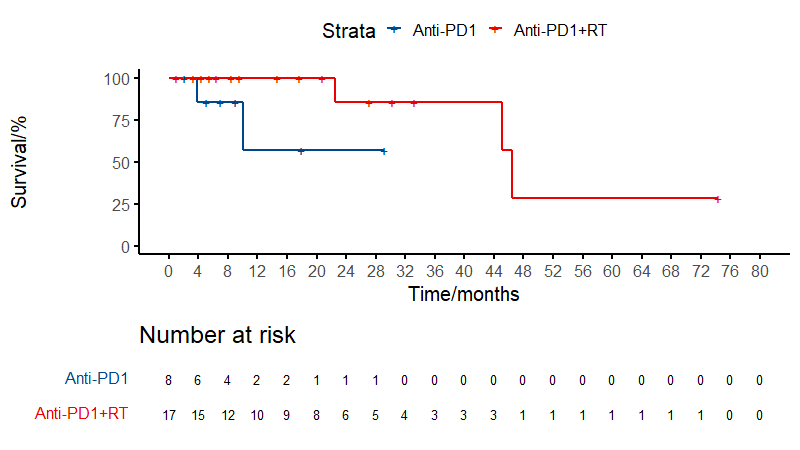 |
| Double-arm | Anti-PD1 vs Anti-PD1+RT | OS | 2B | Umeda et al., 2021 | 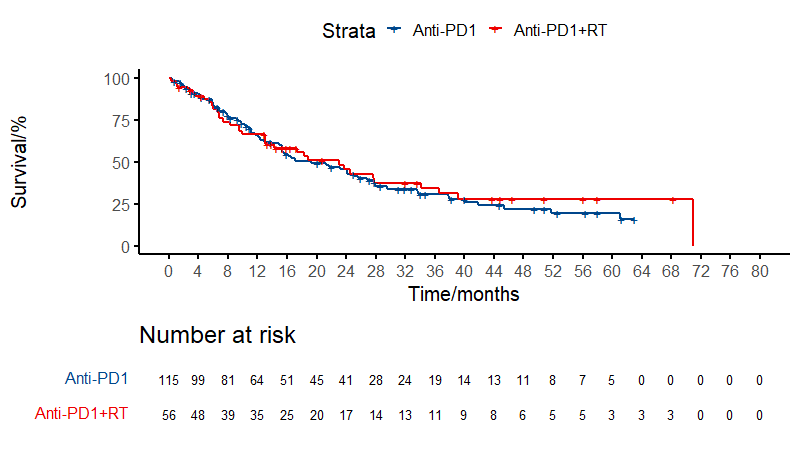 |
| Double-arm | Anti-PD1 vs Anti-PD1+Anti-CTLA4 | OS | 3B | Dimitriou et al., 2022 | 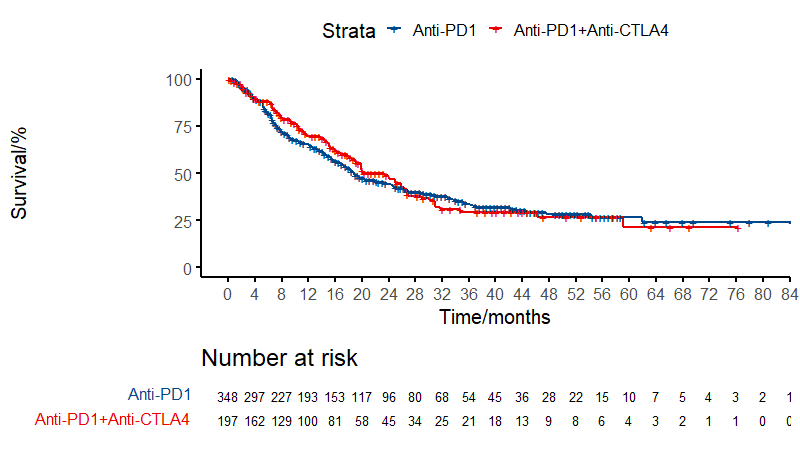 |
| Double-arm | Anti-PD1 vs Anti-PD1+Anti-CTLA4 | OS | 1B | Nakamura et al., 2021 | 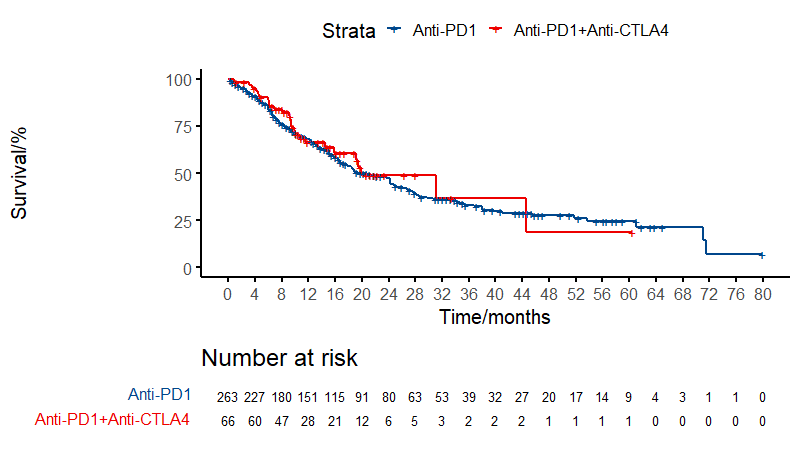 |
| Double-arm | Anti-PD1 vs Anti-PD1+Anti-CTLA4 | OS | 4D | Rose et al., 2021 | 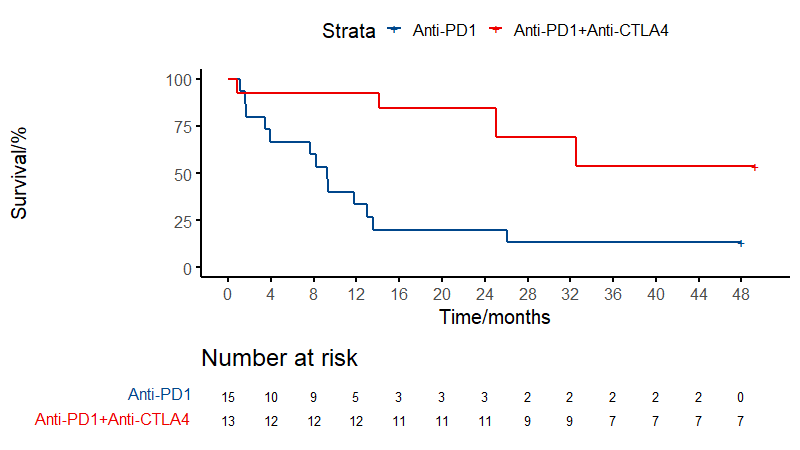 |
| Double-arm | Anti-PD1 vs Anti-PD1+Anti-CTLA4 | OS | 2B, 3B | Umeda et al., 2021 | 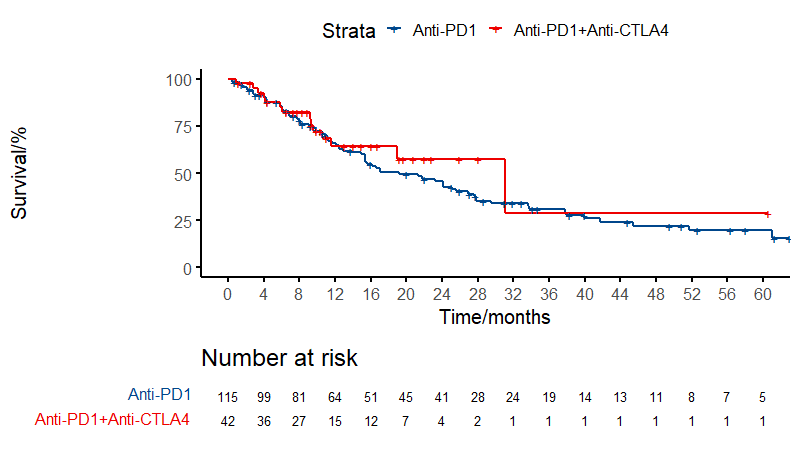 |
| Double-arm | Anti-PD1 vs Anti-PD1+RT | PFS | 4A | Kim et al., 2019 | 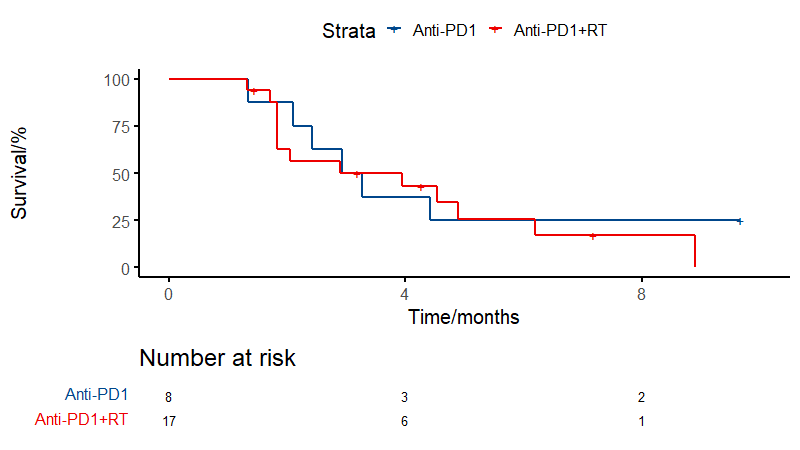 |
| Double-arm | Anti-PD1 vs Anti-PD1+RT | PFS | 2A | Umeda et al., 2021 | 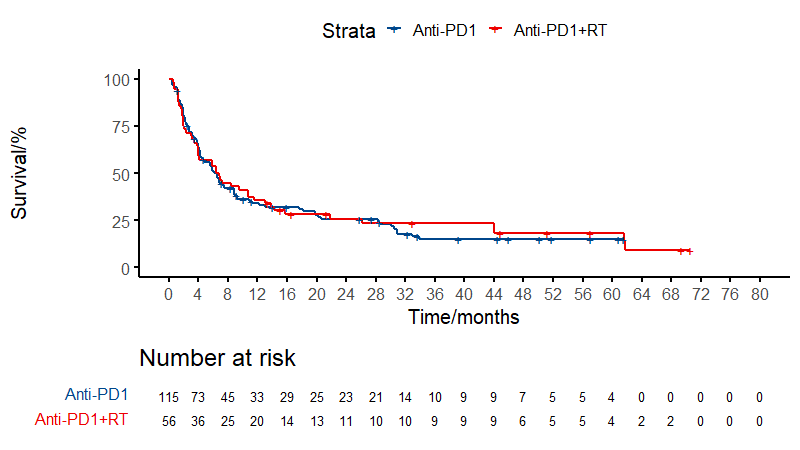 |
| Double-arm | Anti-PD1 vs Anti-PD1+Anti-CTLA4 | PFS | 1B | D’Angelo et al., 2017 | 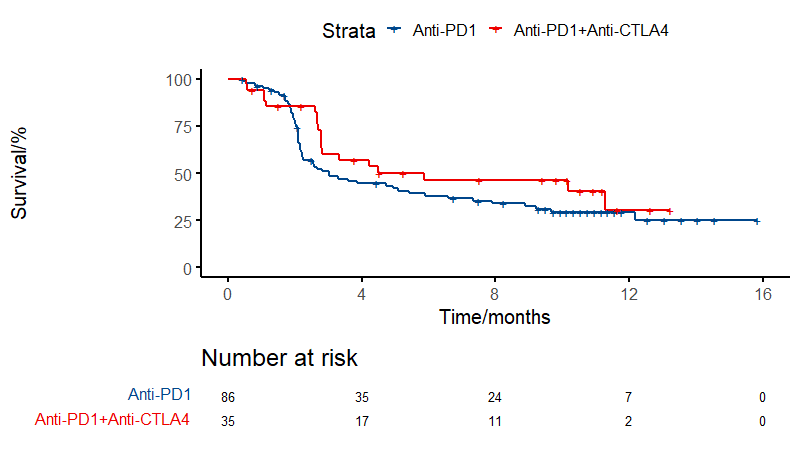 |
| Double-arm | Anti-PD1 vs Anti-PD1+Anti-CTLA4 | PFS | 3A | Dimitriou et al., 2022 | 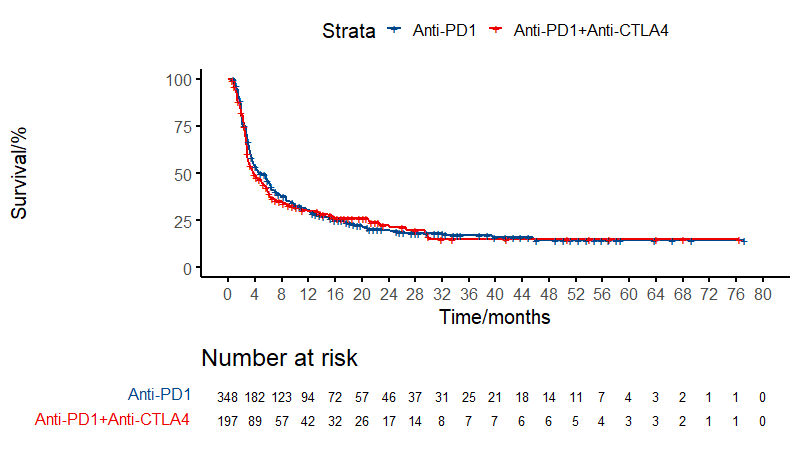 |
| Double-arm | Anti-PD1 vs Anti-PD1+Anti-CTLA4 | PFS | 1A | Nakamura et al., 2021 | 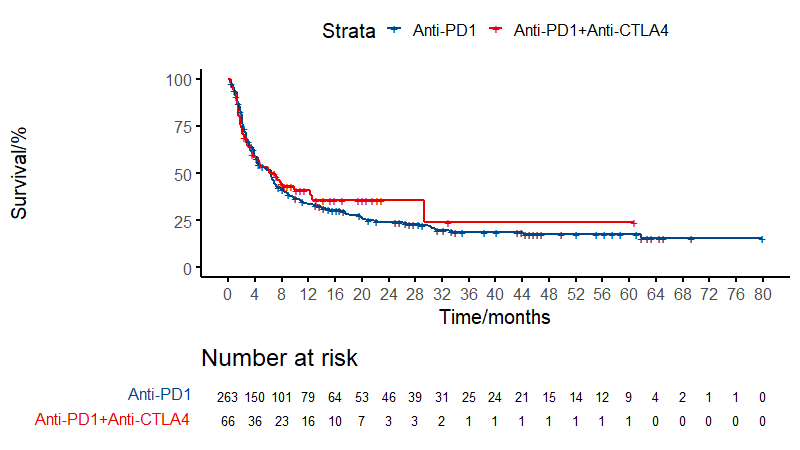 |
| Double-arm | Anti-PD1 vs Anti-PD1+Anti-CTLA4 | PFS | 4C | Rose et al., 2021 | 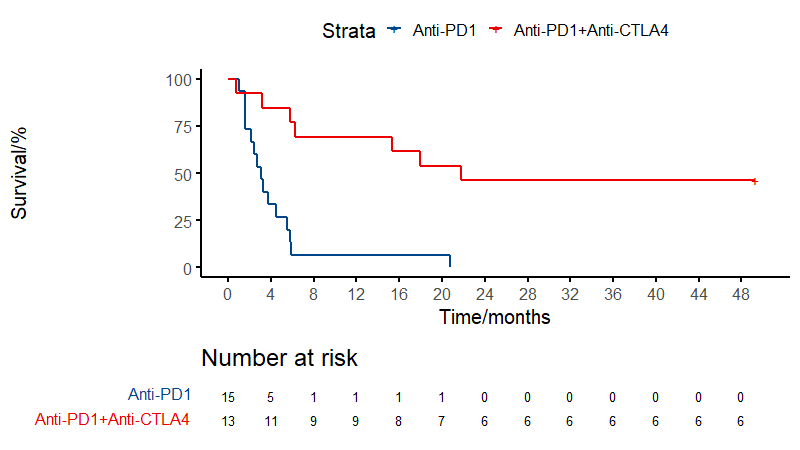 |
| Double-arm | Anti-PD1 vs Anti-PD1+Anti-CTLA4 | PFS | 2A, 3A | Umeda et al., 2021 | 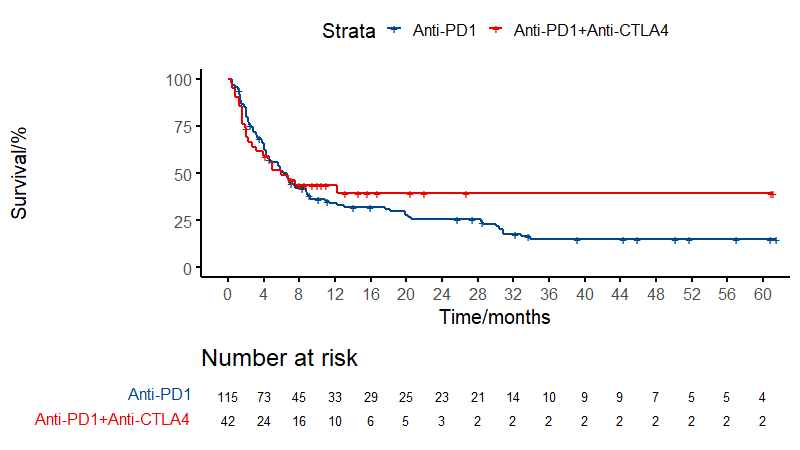 |
| Double-arm | Anti-PD1 vs Anti-CTLA4 | PFS | 1A | D’Angelo et al., 2017 | 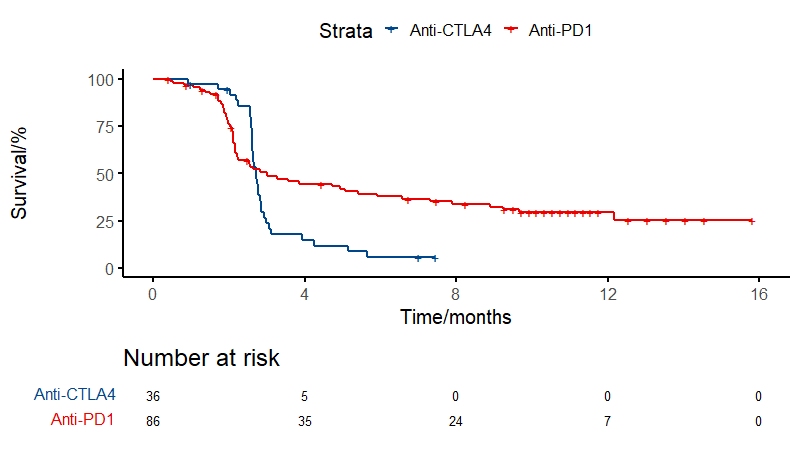 |
| Double-arm | Anti-PD1 vs Anti-CTLA4 | PFS | 2B | Moya-Plana et al., 2019 | 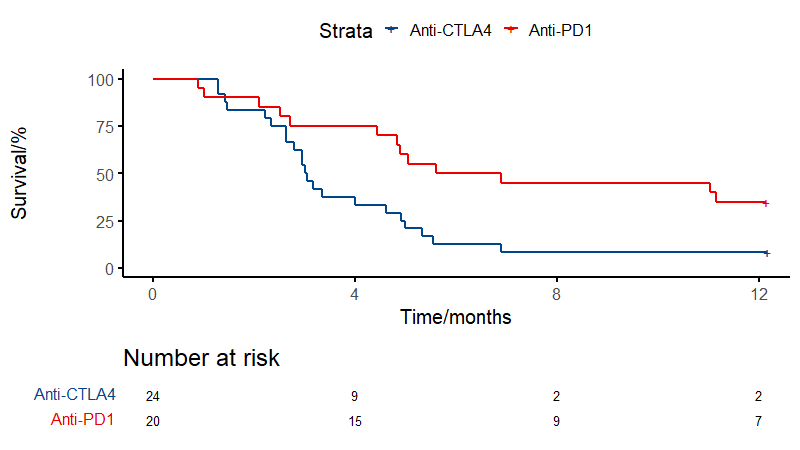 |
| Single-arm | KIT inhibitors | PFS | 2B | Jung et al., 2022 | 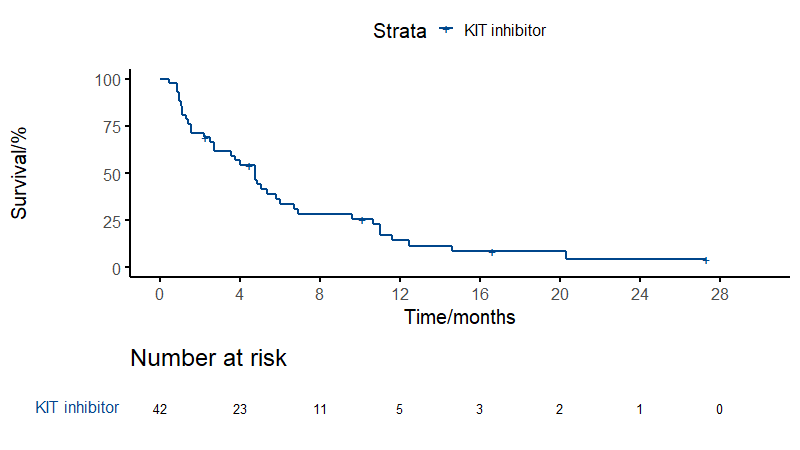 |
| Single-arm | KIT inhibitors | PFS | 2B | Kalinsky et al., 2017 | 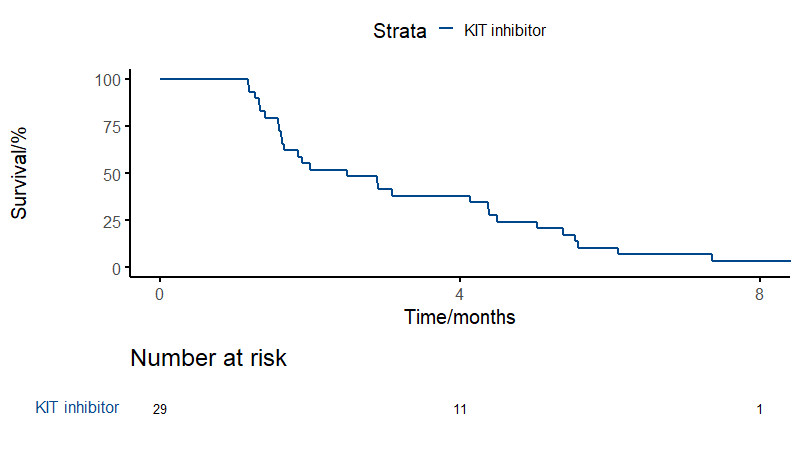 |
| Single-arm | KIT inhibitors | OS | 2E | Jung et al., 2022 | 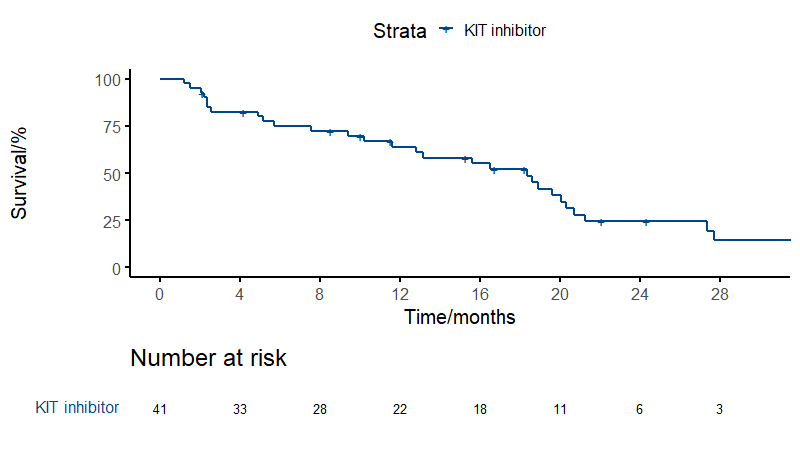 |
| Single-arm | KIT inhibitors | OS | 2D | Kalinsky et al., 2017 | 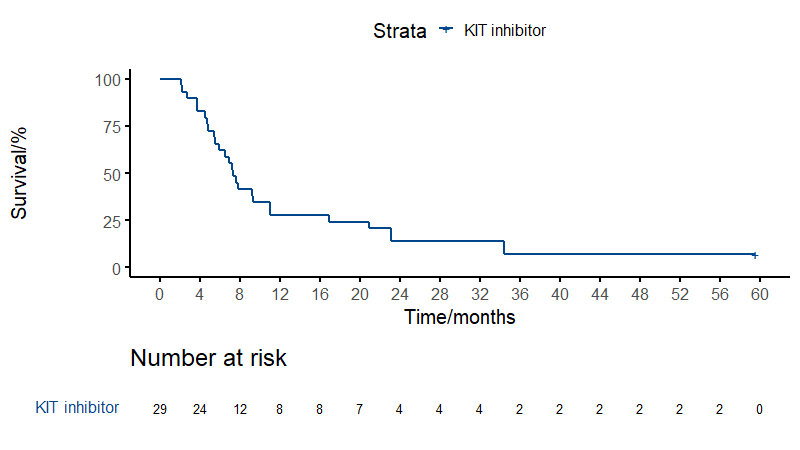 |
